# Recombination events are concentrated in the spike protein region of *Betacoronaviruses*

**DOI:** 10.1371/journal.pgen.1009272

**Published:** 2020-12-17

**Authors:** Louis-Marie Bobay, Angela C. O’Donnell, Howard Ochman

**Affiliations:** 1 Department of Biology, University of North Carolina at Greensboro, Greensboro, North Carolina, United States of America; 2 Department of Integrative Biology, University of Texas at Austin, Austin, Texas, United States of America; Stanford University, UNITED STATES

## Abstract

The *Betacoronaviruses* comprise multiple subgenera whose members have been implicated in human disease. As with SARS, MERS and now SARS-CoV-2, the origin and emergence of new variants are often attributed to events of recombination that alter host tropism or disease severity. In most cases, recombination has been detected by searches for excessively similar genomic regions in divergent strains; however, such analyses are complicated by the high mutation rates of RNA viruses, which can produce sequence similarities in distant strains by convergent mutations. By applying a genome-wide approach that examines the source of individual polymorphisms and that can be tested against null models in which recombination is absent and homoplasies can arise only by convergent mutations, we examine the extent and limits of recombination in *Betacoronaviruses*. We find that recombination accounts for nearly 40% of the polymorphisms circulating in populations and that gene exchange occurs almost exclusively among strains belonging to the same subgenus. Although experimental studies have shown that recombinational exchanges occur at random along the coronaviral genome, in nature, they are vastly overrepresented in regions controlling viral interaction with host cells.

## Author summary

The high mutation rate of RNA viruses makes it problematic to understand and resolve the role of recombination in generating genomic variation. Frequent mutations will (1) increase the likelihood of convergent mutations, particularly in regions subject to strong positive selection, causing sequence similarities that can be mistaken for recombination events, and (2) introduce new changes that accumulate and obscure recognition of past recombination events. We analyzed the patterns of recombination across *Betacoronaviruses* using a dedicated approach to distinguish true recombination from convergent mutations. The *Betacoronaviruses* comprise several populations that could be considered distinct biological species in that they do not engage in gene flow with one another. Moreover, recombination events within the *Sarbecovirus* subgenus, which includes SARS-CoV-2, are highly biased and predominate in the spike protein region, implicating recombination as having a substantial role in host tropism and viral ecology.

## Introduction

Issues surrounding the extent and frequency of recombination in the coronaviruses are of recent concern due to the investigations into the origins of SARS-CoV-2, the etiologic agent of COVID-19. Early analyses of SARS-CoV-2 nucleotide sequences showed that this virus has over 95% sequence similarity to SARS-like bat coronavirus RaTG13, hinting at its possible origins [1]. Although SARS-CoV-2 is most similar to SARS-like strains isolated from bats [1–4], one region of the SARS-CoV-2 genome—the sequence encoding the spike protein that assists in its invasion of host cells—resembles that of a divergent SARS strain isolated from Guangdong pangolins [3]. The receptor-binding domain (RBD) of the SARS-CoV-2 spike protein, a crucial component that enables target cell entry via ACE2 receptor binding, is more similar to the RBD of the strain isolated from pangolins, with 97% sequence identity at the amino-acid level [3,5–7]. In contrast, the RBD regions of SARS-CoV-2 and the bat coronavirus RaTG13 share only 90% amino-acid similarity [1] and overlap in only one of the six amino acids identified as critical residues [3]. The greater similarity of the RBD region to a more distantly related strain raises questions about whether the spike protein of SARS-CoV-2 evolved through a recombination event or, alternatively, by multiple independent mutations followed by selection, causing convergence to the more pangolin-derived sequence.

Since recombination events typically span hundreds of nucleotides, it is usually possible to discriminate between gene exchange and convergent evolution by assessing whether the polymorphisms shared by divergent strains are clumped. However, the rapid rate of coronavirus evolution makes such assessments of sequence ancestry problematic by increasing the likelihood of both convergent mutations (that can result in the false interpretation of recombination events) and diversifying mutations (that can obscure the recognition of past recombination events).

With particular reference to SARS-CoV-2, several studies have tried to circumvent these problems using varied approaches. For example, Lam et al. [8] considered separately the synonymous and non-synonymous substitutions in the region encoding spike protein, reasoning that recombination events would be indifferent to these two classes of substitutions whereas adaptive convergence would mostly be restricted to those mutations causing amino-acid replacements. Subsequently, Wang et al. [9] analyzed the patterns of synonymous substitutions between SARS-CoV-2 and other strains, and identified a rate of substitution more compatible with recombination. The disparity among studies can be traced to difficulties in assessing the high level of variation in this region; however, other analyses have suggested that the receptor-binding motif of SARS-CoV-2—the ~70 amino-acid region within the RBD that directly interacts with the ACE-2 receptor—is a product of recombination with a pangolin-derived coronavirus [7,10].

In this study, we assess the extent to which recombination has affected the evolution of *Betacoronaviruses* as a whole. The *Betacoronaviruses* comprise five major subgenera, of which three, the *Sarbecoviruses* (which encompass the SARS strains), the *Merbecoviruses* (which mostly contain MERS strains) and the *Embevoviruses*, are represented by a sufficient number of sequenced genomes to provide robust analyses of recombination [3,11,12]. Each of these three subgenera forms a monophyletic clade whose members can differ by as much as 25% in sequence, and strains from different subgenera average 50% sequence identity. Despite very high rates of mutation that has led to polymorphisms shared among strains from the same or different subgenera, we find that over a third of the standing variation within *Betacoronaviruses* can be ascribed to recombination. Moreover, recombination in the *Sarbecovirus* subgenus, of which SARS-CoV-2 is a member, is concentrated in genes encoding the spike protein.

## Results

### Recombination within the focal subgenera, *Embevovirus*, *Merbecovirus*, and *Sarbecovirus*

**Fig 1:**
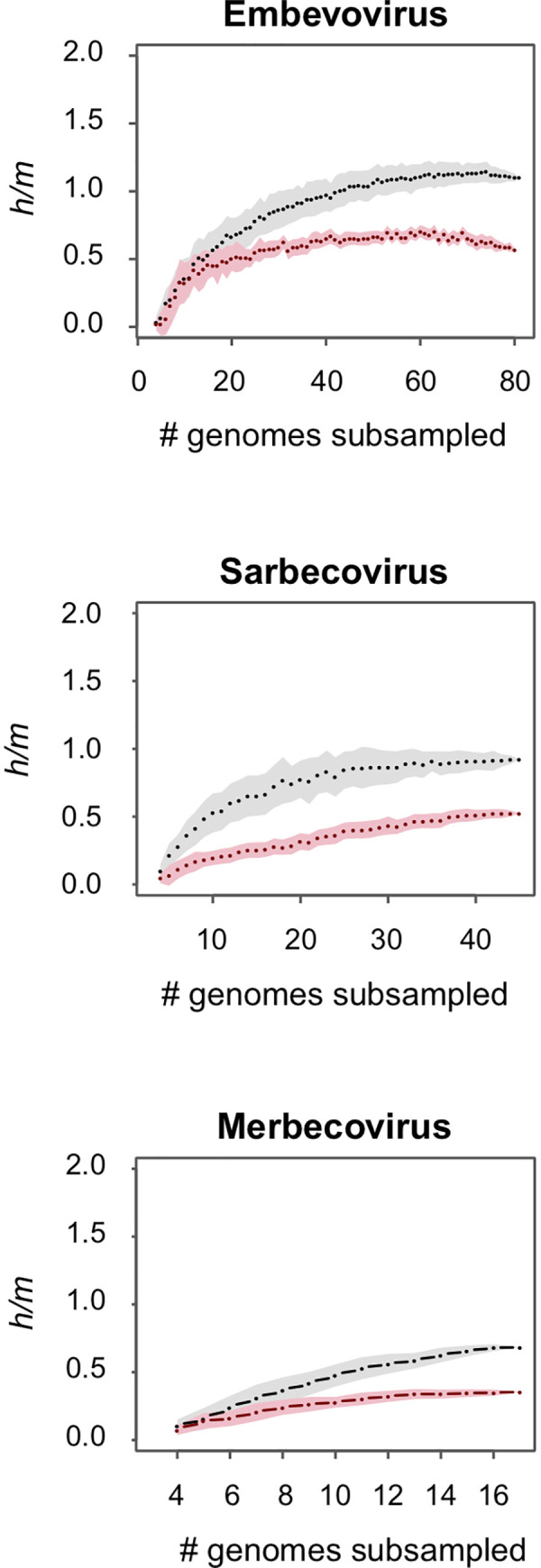
Detecting recombination within *Betacoronavirus* subgenera based on ratios of homoplastic (*h*) to non-homoplastic (*m*) polymorphisms. Within each bivariate plot, black dots and the grey-shaded area denote the median and standard deviation of *h/m* values of the indicated number of subsampled combinations of genomes; and red dots and pink-shaded area denote the median *h/m* values and standard deviation for simulated data in which all homoplasies are introduced by convergent mutations. Differences between the distributions of observed and simulated *h/m* values indicate the extent to which polymorphisms are attributable to recombination. Analyses were performed on the three subgenera for which genomes were sampled to sufficient depths to provide robust results.

We examined the presence of recombination within each of the major subgenera of *Betacoronaviruses* by evaluating the numbers of homoplasies (shared polymorphisms arising from recombination events) relative to the numbers of non-homoplastic polymorphisms (*i*.*e*., mutations). In parallel, we estimated the number of homoplasies expected to result from convergent mutations by simulating genome evolution with mutations and without recombination, while conserving population structure, numbers of polymorphisms, transition/transversion ratio and relative substitution rates across codon positions. Even when considering the mutational process and high level of divergence in *Betacoronaviruses*, we estimate that convergent mutations account for over a third of the homoplasies in each of the subgenera and that there is extensive recombination among their constituent strains. Recombination is highest in the *Embevoviruses* (whose greater sampling depth supplied more polymorphisms), but when considering all polymorphisms in these three subgenera, nearly 40% are introduced by recombination (Fig 1). This metric was estimated from the calculated *h/m* ratio corrected by the number of homoplasies expected to result from convergent mutations, as inferred from simulation.

### Recombination among clades of *Betacoronaviruses*

Prompted by the extent of recombination within each of the *Betacoronavirus* focal subgenera, we sought to determine how readily recombination occurs outside of the boundaries of each of these subgenera. To test for recombination between members of different subgenera, we assessed the effects of incorporating representatives of different subgenera into the analyses, reasoning that the inclusion of a recombining genome would not affect *h/m* whereas inclusion of a non-recombining genome would increase the number of non-homoplastic polymorphisms, thereby lowering *h/m* ratios.

For the three focal subgenera, *Sarbecoviruses*, *Merbecoviruses* and *Embevoviruses*, the addition of a single member from any of the other *Betacoronavirus* subgenera resulted in a substantial reduction in *h/m* ratios, particularly in the *Sarbecoviruses* and *Embevoviruses* (S2 Fig). Despite this reduction, *h/m* values remained much higher than expected if there were a complete absence of recombination between focal subgenera. This pattern could result either (*i*) from lower rates of recombination between subgenera than within subgenera, or (*ii*) from the presence within each subgenus of multiple recombining groups that do not recombine with one another (analogous to having multiple biological species within a subgenus).

To distinguish between these alternatives, we simulated the mutational process—without recombination—by adding similarly diverged sequences to the set of genomes in a focal subgenus. Under these conditions, addition of a sequence simulated exclusively with mutations to each focal subgenus recapitulated the observed pattern of *h/m* reduction (S3 Fig), indicating that none of the three focal subgenera recombines with one another. To test if focal subgenera are composed of multiple subclades that do not recombine with one another, we partitioned the *Embevoviruses* (which had a sufficient number of sequences and phylogenetic structure for this analysis) into nine monophyletic subclades, and examined the extent of recombination between members of these subclades and the largest subclade (subclade 0) within the *Embevoviruses* (S4 Fig). Inclusion of subclades 1, 2 and 3 did not affect the *h/m* ratios, whereas the inclusion of any genome from subgroups 4 to 9 produced a sharp drop in the distribution of *h/m* ratios, as caused by a non-recombining lineage (S4 Fig). This analysis confirms that the *Embevovirus* subgenus comprises several subclades of recombining viruses but that these subclades are not recombining with one another, and they could therefore be considered distinct biological species. (The sample sizes are currently too small to systemically test whether or not each subclade within the *Embevoviruses* is engaging in recombination.)

We then analyzed whether any of the focal subgenera, the *Sarbecoviruses*, *Merbecoviruses* or *Embevoviruses*, displayed signs of recombination with SARS-CoV-2. Supplementing each of these focal clades with the SARS-CoV-2 genome led to a reduction in *h/m* ratios only for the *Embevoviruses*, and the decrease was similar to what was observed above by the inclusion of a non-recombining simulated sequence to this subgroup (S2 Fig). The introduction of the SARS-CoV-2 genome to the *Sarbecoviruses* and *Merbecoviruses* did not substantially modify the *h/m* ratios of these clades (S5 Fig), which can be interpreted as evidence of recombination between SARS-CoV-2 and other *Sarbecoviruses* and/or *Merbecoviruses*. We note though that the results from this analysis alone cannot fully rule-out the possibility that these patterns are due to convergent mutations considering the high levels of sequence divergence, *i*.*e*., saturation.

### Distinguishing between recombination and saturation

The different subgenera of *Betacoronaviruses* do not generally exchange RNA with one another; but because there is only 50% nucleotide sequence identity between the subgenera, the divergence between sequences has reached saturation at many sites producing a high number of homoplasies. Nevertheless, it remains possible that some shared polymorphisms could be due to recombination. At such high levels of sequence divergence, saturation can make it technically challenging to detect true patterns of recombination. However, polymorphisms introduced by recombination produce homoplasies with specific distributional signatures that are not expected for mutations, which occur largely at random. Recombination events are typically short, and the polymorphisms within the transferred RNA segment are expected to be distributed similarly across strains, *i*.*e*., homoplasies generated by a recombination event should have the same (or nearly the same) distribution across genomes, and homoplasies with similar distributions are expected to be found near each other.

To distinguish which homoplasies likely stemmed from recombination rather than from convergent mutations, we searched clusters of homoplasies that were the same across genomes, reasoning that homoplasies that are truly the result of recombination should be located in proximity and distributed across a similar set of genomes. We detected several clusters of homoplastic alleles that displayed the same or similar distributions across genomes and that were located in the same genomic vicinity (compared to random expectations). Of particular interest, we detected an array of 11 clustered homoplasies with similar genome distributions in SARS-CoV-2 and two genomes of bat *Merbecoviruses* isolated from Wuhan China (MG021452 and MG021451). However, simulations revealed that this pattern has ~5% probability of resulting from convergent mutations given the extent of saturation in the dataset. The vast majority of these substitutions did not alter the amino sequence of the encoded NSP12 and NSP13 proteins, indicating that they are unlikely to be adaptive in SARS-CoV-2.

### Gene-by-gene recombination within *Sarbecoviruses*

**Fig 2:**
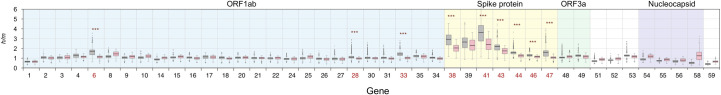
Extent of recombination evident for each gene in *Sarbecovirus* genomes. For each annotated gene (numbered), we calculated *h/m* ratios based on available sequences (grey) and on simulated datasets (pink). Box-and-whiskers plots of each gene show median (black line), interquartile range IQR (box), and 1.5*IQR (whiskers), and asterisks (and gene numbers highlighted in red) mark instances in which observed and simulated datasets differed significantly (*p* < 10^−5^, one-sided Wilcoxon test with Bonferroni correction) due to an excess of polymorphisms introduced by recombination.

The presence of genome-wide recombination in *Sarbecoviruses* and, specifically, the probable recombinant within the NSP12 and NSP13 genes of SARS-CoV-2, prompted us to further investigate how the patterns of recombination vary across *Sarbecovirus* genomes. We quantified the ratios of homoplastic to non-homoplastic polymorphisms for each of the 43 genes conserved among members of this subgroup and detected variable patterns of recombination among the genes. Most genes (79%) showed non-significant or weak increases of *h/m* ratios relative to expectations under mutation only, but nine displayed highly significant increases in *h/m* ratios (Fig 2). Nearly all the genes encoding subunits of the spike protein display highly significant (>1.2-fold, *p* < 10^−5^, Wilcoxon test with Bonferroni correction) increases in *h/m* ratios. Aside from those in the spike protein region, only three CDSs—CDS6, CDS28 and CDS33—which are located within the large ORF1ab polyprotein, show significant, >1.2-fold increases in *h/m* ratios. These results indicate that the pattern of recombination along the *Sarbecovirus* genome predominantly affects genes encoding the spike protein subunits.

### So far, no evidence of recombination among SARS-CoV-2 genomes

**Fig 3:**
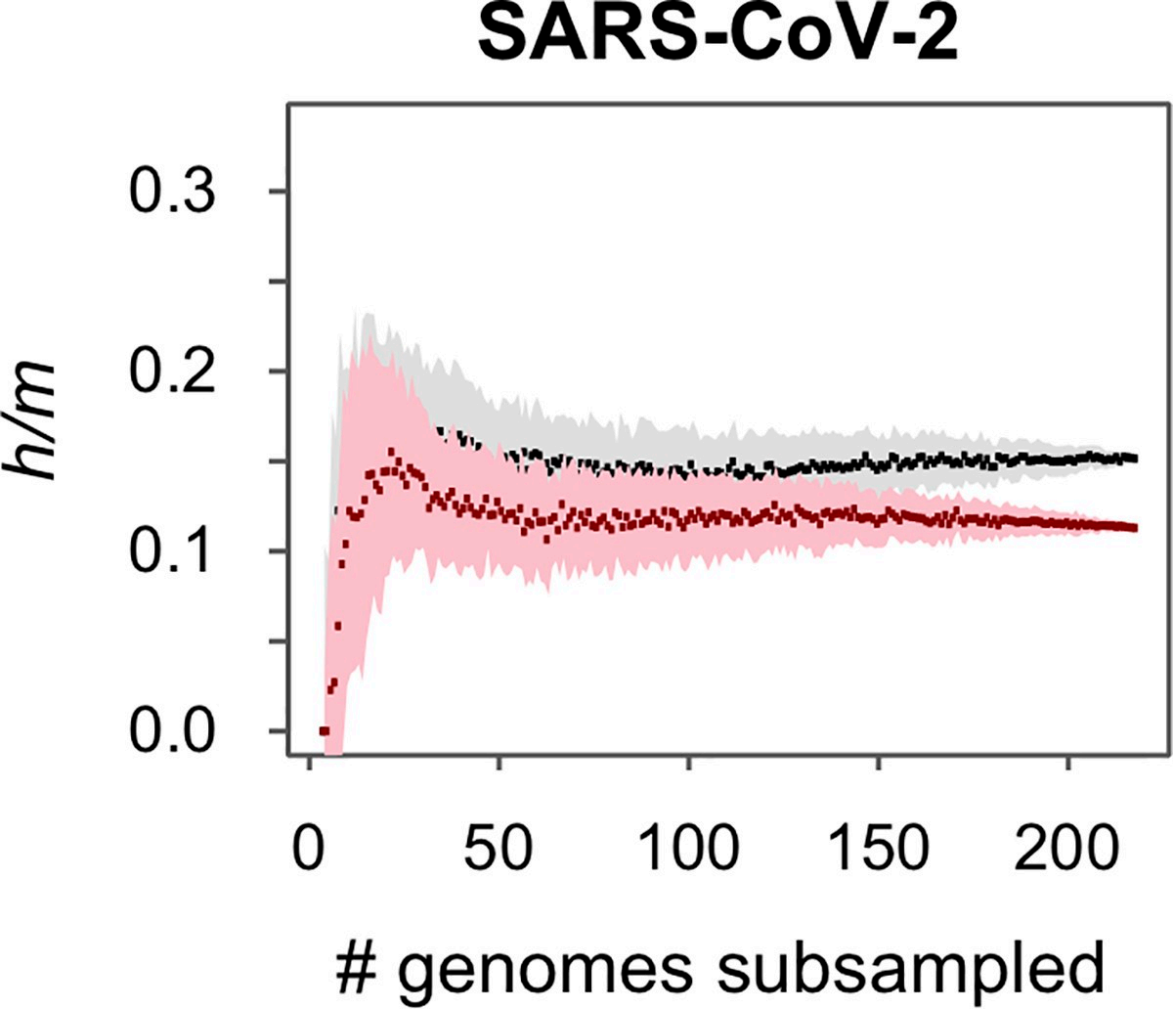
Signal of recombination in strains of SARS-Cov-2. Pattern of gene flow based on *h/m* ratios was analyzed in SARS-CoV-2 using 218 sequenced strains. As in Fig 1, black dots and the grey-shaded area denote the median and standard deviation of *h/m* values of the indicated number of subsampled combinations of genomes; and red dots and pink-shaded area denote the median *h/m* values and standard deviation for simulated data in which all homoplasies are introduced by convergent mutations.

Although the COVID-19 epidemic is recent, the documented evidence of recombination within *Sarbecoviruses*, and the high number of SARS-CoV-2 variants and infected hosts, present a situation where recombination is liable. We searched for evidence of recombination using a set of 218 sequenced genomes of SARS-CoV-2 (S3 Table) and estimated *h/m* ratios. In parallel, we simulated the evolution of these genomes as in previous analyses by exclusively introducing mutations that reflect the transition/transversion ratio of these sequences and by imposing selective constraints across codon positions. Compared to the large number of homoplasies observed when examining variation within *Sarbecoviruses* as a whole, there were few homoplasies unique to the SARS-CoV-2 strains and other *Betacoronaviruses*, and we detected no evidence of recombination events restricted to these genomes (Fig 3).

## Discussion

*Betacoronaviruses* are subject to high rates of mutation and recombination, and both processes have been linked to the emergence of strains causing zoonotic outbreaks. Among the most notable cases involve those viruses responsible for human SARS and MERS. In the case of SARS, recombination between avian and mammalian coronaviruses have been implicated in the switch to human hosts [13–15], and in MERS, a recombinant lineage present in camels gave rise to the outbreaks in humans and increased human-to-human transmission [16,17]. Additionally, there are several other cases in which recombination events are thought to have mediated the changes necessary to make jumps between host species, including feline infectious peritonitis virus type 2 [18] as well as other human coronaviruses, such as HCoV-OC43 [19,20].

Recombination can offer a rapid means of acquiring gene variants that facilitate host switching; however, it is challenging to identify specific recombination events that are responsible for adaptation to new hosts. When inspected for phylogenetic discordant regions, many coronaviruses display evidence of past, sometimes ancient, events of recombination, but it is also possible that the similarity between genomic segments in otherwise divergent viruses is instead caused by convergent mutations [3,21,22]. By applying a site-by-site approach to establish which genomes regularly exchange genes–in effect, defining biological species within the *Betacoronaviruses*–we can recognize the subsets of strains that can be considered conspecifics, thereby allowing us to both ignore sporadic recombination events and account for convergence.

Our approach was developed to disentangle the relative contributions of recombination and mutation events. We corrected our estimates using simulated null models that predict homoplasies derived only from convergent mutations. Additionally, it is expected that homoplasies that are introduced by convergent mutations will manifest mostly at random, whereas homoplasies associated with recombination are typically clustered and distributed identically across divergent strains. Using these features to discriminate events of recombination from convergent mutation will enable future methodologies to detect signals of recombination in highly saturated genomes such as groups of divergent RNA viruses. Taken together, these analyses allow for recombination to be more confidently estimated and by extension, help distinguish between recombination and the saturation levels expected in more divergent comparisons.

Despite experimental demonstration that divergent coronaviruses can recombine [23,24] and reports of recombination events between distantly related coronaviruses [25], most retrospective sequence analyses find that recombination is largely limited to closely related strains [9,11,26,27]. This disparity could arise because events of inter-strain recombination rely on co-infection and more similar strains tend to infect a particular host species, thereby making the likelihood of recombination between divergent lineages less common. By analyzing the diversity within natural populations of *Betacoronaviruses*, we find that each of the major subgenera–the *Embevoviruses* the *Merbecoviruses* and the *Sarbecoviruses*–is reproductively isolated, with gene exchange restricted to members of the same subgroup. We note that in previous comparisons across ranges of viral taxa [28], *Betacoronovirus* was the only viral genus that does not constitute a single biological species, which we ascribed to the presence of multiple sexually isolated subgroups; however, the sampling at that time was too limited to test this possibility.

The rates and patterns of homologous exchange have been assessed experimentally in coronaviruses. During mixed infections of cell cultures with murine coronaviruses, at least 10% of progeny viruses were recombinants showing multiple independent recombination breakpoints [29]. The process itself appears to be unbiased, occurring at random sites across the genome [30]; but in nature, events of recombination are concentrated by natural selection in regions with roles in host interactions, which among coronaviruses is centered in spike proteins [3,5]. Because coronavirus spike proteins mediate entry into host cells, these changes have been cited as possible prompts for virus adaptation to new hosts, possibly triggering zoonoses [10].

In our gene-by-gene analyses of *h/m* ratios in *Sarbecoviruses*, nearly all annotated CDSs encoding subunits of the spike protein showed signs of excessive recombination, consistent with the previous phylogeny-based evidence of both ancient and recent recombination events in the spike protein in coronaviruses [2,15,22,31–34]. By extension, recombination has recently been examined in SARS-CoV-2 and is targeted to regions encoding the spike protein [9,10,35]. Upon the initial sequencing of the SARS-CoV-2 genome, it was noted that its spike protein RBD was most similar to the RBD region of coronaviruses isolated from pangolins, although its closest relative at the whole-genome level was the SARS-like bat coronavirus, RaTG13 [3,7–9,22]. And because the spike protein RBD region of SARS-CoV-2 also shows greater synonymous divergence from the RaTG13 than from the pangolin (GD410721) RBD regions, the collective evidence has been interpreted as supporting the hypothesis that the SARS-CoV-2 spike protein RBD arose via recombination, possibly from a pangolin coronavirus [9,36,37]. But on account of the sequence divergence between the SARS-CoV-2 spike protein RBD and its potential source, this recombination event is not likely to have been responsible for its recent emergence. A more probable scenario is that it resulted from a combination of earlier recombination events and accumulated mutations [8,9,35,37]. The role of the coronavirus spike protein in host tropism and pathogenicity implies that modifications at these sites are likely to modify virulence or expand host range, thereby conferring an advantage to the virus. Accordingly, the higher *h/m* ratios in spike protein regions, as detected in this study, is best explained under a model of recombination followed by positive or diversifying selection.

Due to the high frequency of mutations and the strong selective pressures affecting coronavirus evolution, it is difficult to ascertain whether putative cases of allelic transfer are attributable to recombination or to convergent mutations [3,9,22], and most comparative approaches used to infer recombinant variants are incapable of distinguishing recombining sequences from rapidly evolving sequences [38]. To overcome such problems, we generated null models of sequence evolution by systematically simulating sequence evolution and testing the patterns of homoplasies expected to result from convergent mutations. These analyses revealed that most genes in members of the *Sarbecovirus* subgenus do not display substantial levels of recombination except in those genes encoding the spike protein region.

Our analysis accounted for the contribution of convergent mutations through the use of simulated null models; however, other factors could potentially influence our results. Our null models were designed to capture homoplasies derived entirely from convergent mutations, but such models do not perfectly account for all factors that can affect the accumulation of variants, such as population structure, clonal interference and fluctuations in population sizes. Additionally, inferences about recombination events can be complicated in RNA viruses by their rapid rate of evolution and the potential role of selection among the diverse genes of the genomes. Therefore, we interpreted results derived from these models rather conservatively: for example, when our tests for recombination between the three *Betacoronavirus* subgenera showed only modest increases in *h/m* ratios relative to the null model, we inferred no clear evidence of recombination. Despite the fast rate of sequence evolution in RNA viruses, alleles exchanged among very recently diverged genomes are unlikely to generate homoplasies and remain undetectable, thereby preventing recognition of recombination events. Notwithstanding these shortcomings, our method offers a multifaceted approach that can gauge the contribution of recombination while accounting for the input of convergent mutations.

Though not extensively explored in our analyses, patterns of recombination can be extended to taxonomic classification of viral species. In their recent classification of SARS-CoV-2, the *Coronaviridae* Study Group of the ICTV [39] based the taxonomic placement and naming of SARS-CoV-2 on phylogenetic criteria—a potentially misleading metric in that high rates of recombination and mutation can produce independent and conflicting origins for different portions of the genome [37]. However, the viral species concept described by the *Coronaviridae* Study Group [39] mirrors, in many ways, the system used to organize cellular organisms into biological species (and, in fact, they make repeated reference and comparison to the taxonomic classification of humans). Given the commonalities that they note, we suggest that coronaviruses be defined according to the Biological Species Concept (BSC), which delineates species based on the ability of its constituents to recombine. Application of the BSC to viruses can both account for the genetic variability of strains and eliminate the use of arbitrary and different sequence-identity thresholds or phenotypic characters when classifying viruses into species. Recombination is not the sole driving force behind the emergence of new viruses [11,21,31], but knowledge and detection of its occurrence provides a useful strategy for defining species boundaries [28], and in understanding the origins of new strains.

## Methods

### Genomes analyzed

Full genome sequences from a total of 1,422 *Betacoronaviruses* were downloaded from the Virus Pathogen Database (https://www.viprbrc.org) in March 2020 (S1 Table). A core genome comprising the set of genes common to all strains was built with the *ConSpeciFix* pipeline [40], and a matrix of core genome distances was calculated in RAxML v8 using the GTR + GAMMA model [41]. The dataset was dereplicated through the removal of highly similar genomes, those with *D* < 0.0001, where *D* represents genome distances in substitutions per nucleotide, resulting in a dataset containing 168 genomes (S1 Fig and S2 Table). We built a phylogenetic tree from the core genome of these genomes using RAxML v8 using the GTR + GAMMA model. From this tree and previous classifications, the following clades were defined: *Embevovirus*, *Hibecovirus*, *Merbecovirus*, *Nobecovirus* and *Sarbecovirus* (representing the five recognized subgenera of *Betacoronaviruses*), and HKU4 and HKU5 (each containing seven strains isolated only from bats).

### Recombination analysis

Within the framework of *ConSpeciFix*, we evaluated whether polymorphic sites within and among clades of *Betacoronaviruses* were more likely to be attributable to mutation or to recombination. We used the three largest subgenera, *Embevovirus* (*n* = 81), *Merbecovirus* (*n* = 17) and *Sarbecovirus* (*n* = 45), as focal clades, and used a resampling approach to estimate the ratio of homoplastic (*h*) polymorphisms (sites that could not be parsimoniously ascribed to vertical ancestry; *i*.*e*., recombinant alleles) to non-homoplastic (*m*) polymorphisms (vertically transmitted alleles) for each subgroup.

Each of the three focal clades was then used as reference to individually test a selected SARS-CoV-2 genome (MT106054) or a genome from each of the other clades for recombination with members of the focal clade (S2 Table). Orthology was inferred by applying a 50% nucleotide identity threshold and 80% sequence length conservation, and each group of orthologs was tested for the inclusion of paralogs as described in Bobay & Ochman [28]. We then calculated the *h/m* ratios of each focal clade with the inclusion of the SARS-CoV-2 genome or with the inclusion of a genome from each of the other clades, and compared these *h/m* ratios to those calculated for each focal clade alone.

### Simulations

Because homoplasies can be introduced by convergent mutations as well as by recombination, we conducted genome simulations using *CoreSimul* [42] to establish the frequency of shared polymorphisms expected to occur solely by mutational processes. For each core genome alignment, we built a phylogenetic tree in RAxML v8 using the GTR + GAMMA model, and we estimated the transition to transversion ratio κ. To account for biases in the nucleotide composition of each dataset, a random core-genome concatenate was selected to initiate each iteration of the simulations. For each dataset, the core-genome concatenate was evolved along the tree with *CoreSimul* without recombination and using a substitution model that mirrors the transition/transversion ratio κ of each dataset. To account for negative selection, we further evolved different codon positions at different rates while maintaining a constant substitution rate overall. Relative substitution rates across codon positions were set as follows: 0.1, 0.05 and 0.85 for codon positions one, two and three, respectively. Importantly, phylogenetic trees were rescaled in order to match the number of polymorphisms observed in each dataset, as specified in *CoreSimul* [42]. Simulated alignments were then subjected to *ConSpeciFix* analysis to estimate the *h/m* ratios expected to result solely from convergent mutations.

## Supporting information

S1 Fig Phylogeny of *Betacoronaviruses*. Recognized subgenera are labelled and colored-coded. The phylogenetic tree was built on the concatenate of the core genome, *i*.*e*. the set of genes shared across nearly all genomes, using RAxML v8, with the GTR + GAMMA model. Scale bar represents nucleotide substitutions per site.
(TIF)

S2 Fig Testing for recombination between subgenera of *Betacoronaviruses*. For each focal subgenus, *Embevovirus*, *Merbecovirus* or *Sarbecovirus*, a randomly picked representative from one of the other six *Betacoronavirus* subgenera (denoted in heading of each bivariate plot) is pooled with the genomes of the focal subgenus, and *h/m* ratios are calculated. Black dots and the grey-shaded area denote the median and standard deviation of *h/m* values of the indicated number of subsampled combinations of genomes; and red dots and pink-shaded area denote the median *h/m* values and standard deviation for simulated data in which all homoplasies are introduced by convergent mutations. Red dashed lines indicate the maximum median *h/m* value for the focal subgenus when a member of a different subgenus was not included.
(TIF)

S3 Fig Testing for recombination within the focal subgenera of *Betacoronaviruses* using simulated sequences. Each bivariate plot shows *h/m* ratios calculated on a *Betacoronavirus* subgenus, *Embevovirus*, *Merbecovirus* or *Sarbecovirus*), with black dots and grey shading denoting the median and standard deviation of *h/m* values of the indicated number of subsampled combinations of genomes; and *h/m* ratios calculated on the same subgenus after adding a sequence simulated exclusively with mutations corresponding to the substitutional pattern of that subgenus, shown with black dots and blue shading.
(TIFF)

S4 Fig Phylogenetic substructure and recombination in *Embevoviruses*. **a,** Clades within the subgenus *Embevovirus* are numbered (1–9) along with the focal subclade (0), which, as the most populated subclade, was used as the foundational group to analyze recombination between subclades. Strains enclosed within the blue box (members of subclades 0, 1, 2 and 3) form a recombining group (*i*.*e*., a single biological species) that does not exchange genes with members of other subclades. **b,** Gene exchange between subclades of *Embevoviruses* were tested by first calculating *h/m* ratios on focal subclade 0 (top left panel), and then a randomly picked representative from one of the other nine *Embevovirus* subclades (1–9; denoted by the heading of bivariate plot) was pooled with the genomes of the focal subclade, and *h/m* ratios were re-calculated. Black dots and the grey-shaded area denote the median and standard deviation of *h/m* values of the indicated number of subsampled combinations of genomes. As noted in green-shaded panel, inclusion of members of *Embevovirus* subclade 1, 2, or 3 do not affect *h/m* ratios (indicating that they, along with members of focal subclade 0, are all members of the same species), whereas inclusion of members of *Embevovirus* subclade 4, 5, 6, 7, 8, or 9 cause steep declines in *h/m* ratios (indicating their lack of recombination with focal subclade 0).
(TIF)

S5 Fig Testing for gene exchange between SARS-CoV-2 and members of each focal clade. Each bivariate plot shows *h/m* ratios calculated on a *Betacoronavirus* subgenus (*Embevovirus*, *Merbecovirus* or *Sarbecovirus*) with black dots and grey shading denoting the median and standard deviation of *h/m* values of the indicated number of subsampled combinations of genomes; and *h/m* ratios calculated on the same subgroup after the addition of a SARS-CoV-2 genome, shown with black dots and yellow shading.
(TIFF)

S1 Table List of genomes downloaded. (DOCX)

S2 Table List of analyzed genomes and taxonomic information. (DOCX)

S3 Table List of SARS-CoV-2 genomes analyzed. (DOCX)
