# Supplementary figures and images for: Recombination events are concentrated in the spike protein region of Betacoronaviruses
Source: PLoS Genet. 2020 Dec 17;16(12):e1009272. doi: 10.1371/journal.pgen.1009272 (PMC7775116; doi:10.1371/journal.pgen.1009272)

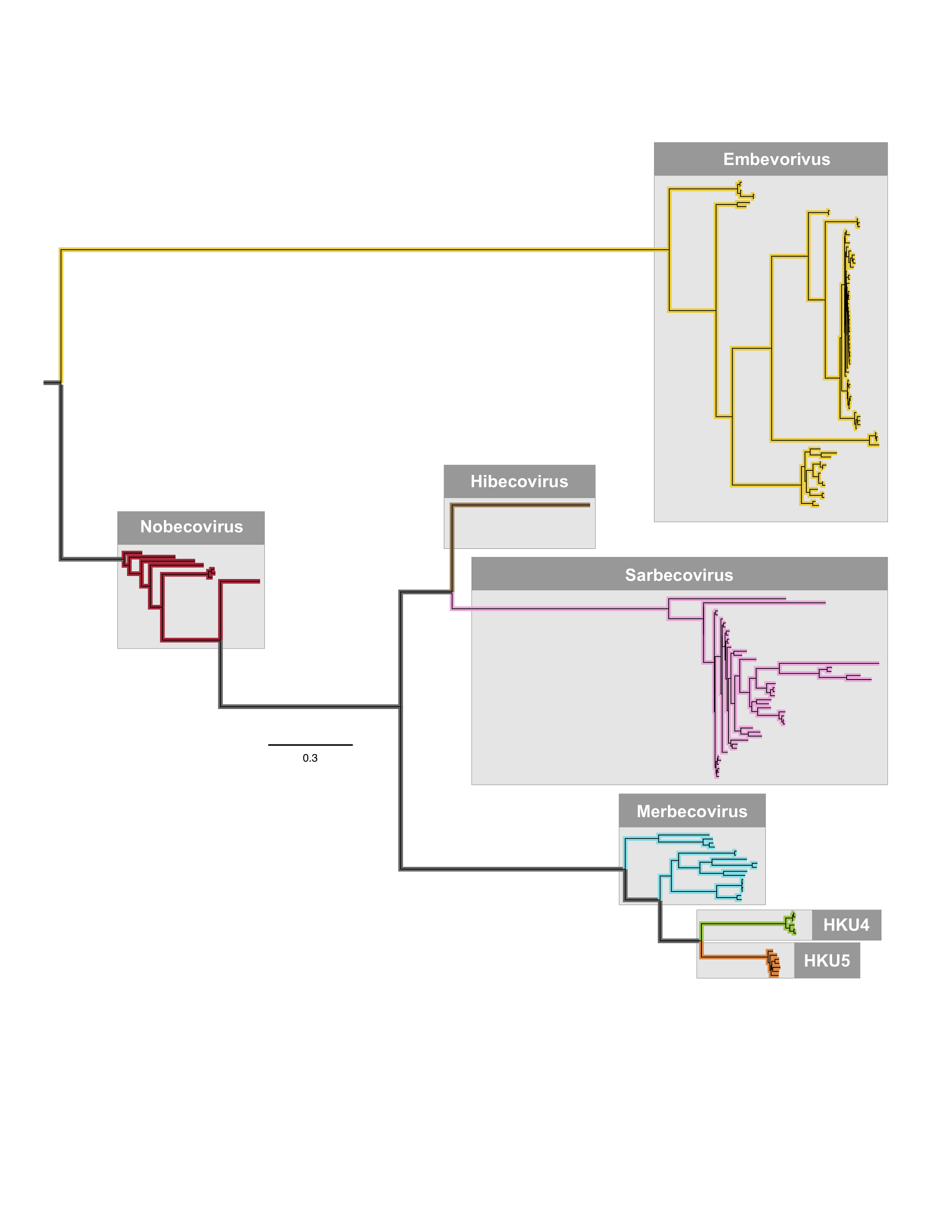

Supplement: S1 Fig — Recognized subgenera are labelled and colored-coded. The phylogenetic tree was built on the concatenate of the core genome, i.e. the set of genes shared across nearly all genomes, using RAxML v8, with the GTR + GAMMA model. Scale bar represents nucleotide substitutions per site. (TIF) [file pgen.1009272.s001.tif]

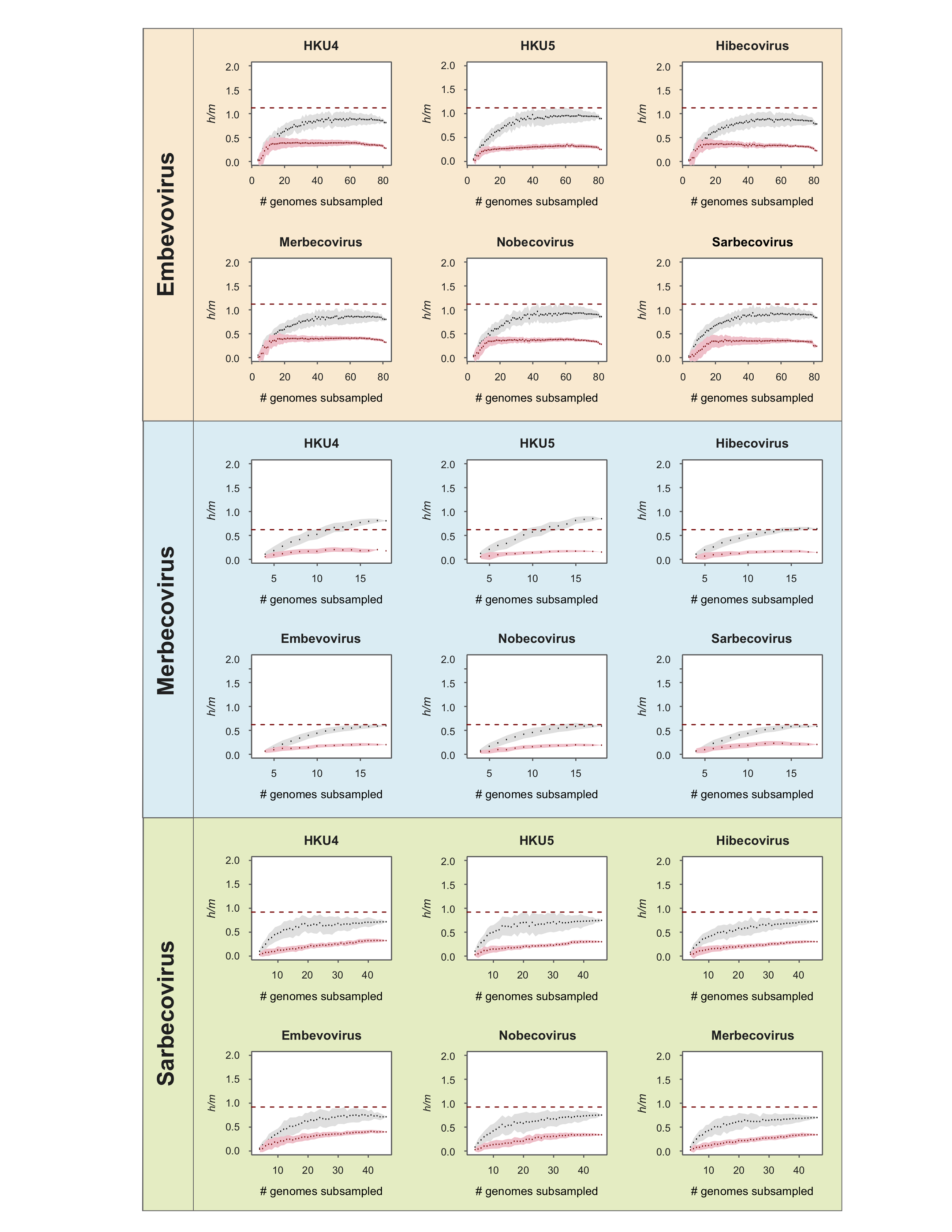

Supplement: S2 Fig — For each focal subgenus, Embevovirus, Merbecovirus or Sarbecovirus, a randomly picked representative from one of the other six Betacoronavirus subgenera (denoted in heading of each bivariate plot) is pooled with the genomes of the focal subgenus, and h/m ratios are calculated. Black dots and the grey-shaded area denote the median and standard deviation of h/m values of the indicated number of subsampled combinations of genomes; and red dots and pink-shaded area denote the median h/m values and standard deviation for simulated data in which all homoplasies are introduced by convergent mutations. Red dashed lines indicate the maximum median h/m value for the focal subgenus when a member of a different subgenus was not included. (TIF) [file pgen.1009272.s002.tif]

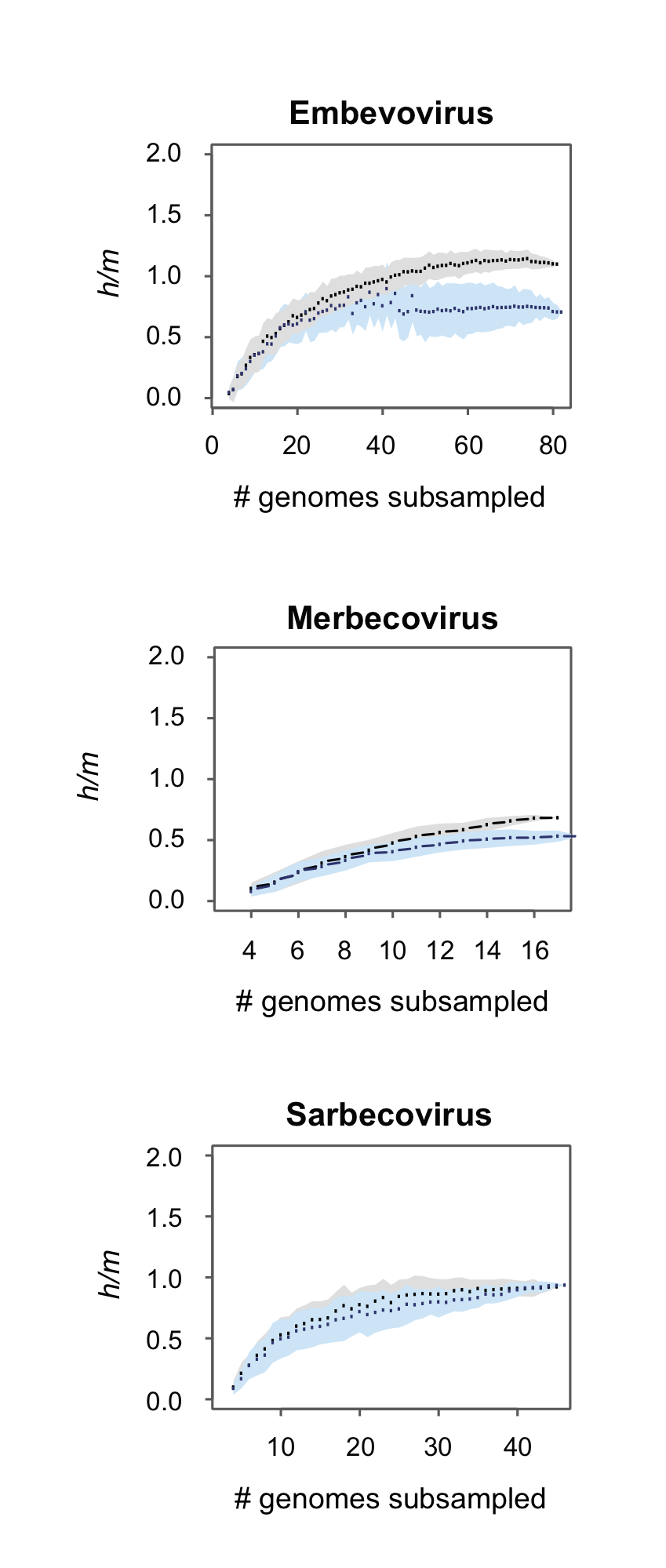

Supplement: S3 Fig — Each bivariate plot shows h/m ratios calculated on a Betacoronavirus subgenus, Embevovirus, Merbecovirus or Sarbecovirus), with black dots and grey shading denoting the median and standard deviation of h/m values of the indicated number of subsampled combinations of genomes; and h/m ratios calculated on the same subgenus after adding a sequence simulated exclusively with mutations corresponding to the substitutional pattern of that subgenus, shown with black dots and blue shading. (TIFF) [file pgen.1009272.s003.tiff]

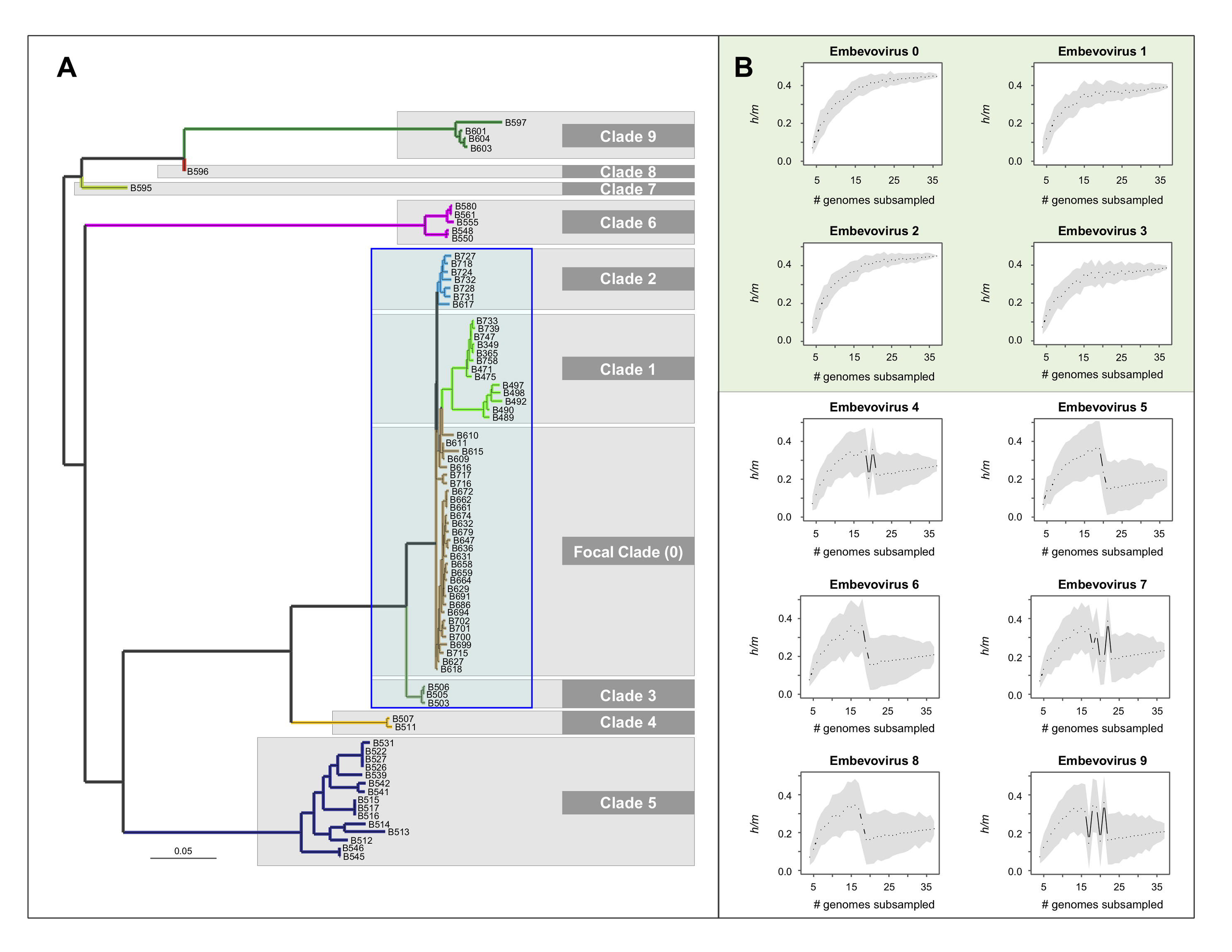

Supplement: S4 Fig — a, Clades within the subgenus Embevovirus are numbered (1–9) along with the focal subclade (0), which, as the most populated subclade, was used as the foundational group to analyze recombination between subclades. Strains enclosed within the blue box (members of subclades 0, 1, 2 and 3) form a recombining group (i.e., a single biological species) that does not exchange genes with members of other subclades. b, Gene exchange between subclades of Embevoviruses were tested by first calculating h/m ratios on focal subclade 0 (top left panel), and then a randomly picked representative from one of the other nine Embevovirus subclades (1–9; denoted by the heading of bivariate plot) was pooled with the genomes of the focal subclade, and h/m ratios were re-calculated. Black dots and the grey-shaded area denote the median and standard deviation of h/m values of the indicated number of subsampled combinations of genomes. As noted in green-shaded panel, inclusion of members of Embevovirus subclade 1, 2, or 3 do not affect h/m ratios (indicating that they, along with members of focal subclade 0, are all members of the same species), whereas inclusion of members of Embevovirus subclade 4, 5, 6, 7, 8, or 9 cause steep declines in h/m ratios (indicating their lack of recombination with focal subclade 0). (TIF) [file pgen.1009272.s004.tif]

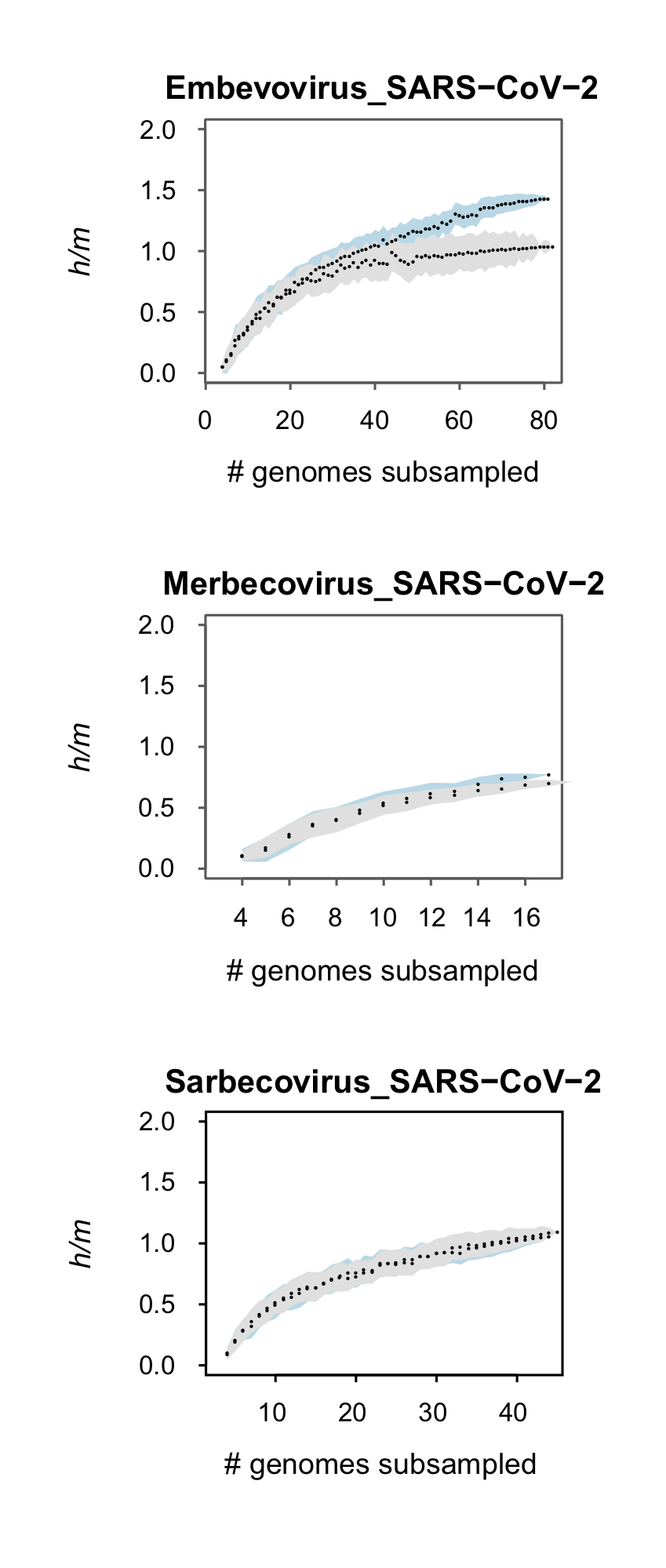

Supplement: S5 Fig — Each bivariate plot shows h/m ratios calculated on a Betacoronavirus subgenus (Embevovirus, Merbecovirus or Sarbecovirus) with black dots and grey shading denoting the median and standard deviation of h/m values of the indicated number of subsampled combinations of genomes; and h/m ratios calculated on the same subgroup after the addition of a SARS-CoV-2 genome, shown with black dots and yellow shading. (TIFF) [file pgen.1009272.s005.tiff]
