# Supplementary material for: Recombination events are concentrated in the spike protein region of Betacoronaviruses
Source: PLoS Genet. 2020 Dec 17;16(12):e1009272. doi: 10.1371/journal.pgen.1009272 (PMC7775116; doi:10.1371/journal.pgen.1009272)
Supplement: S1 Table — (DOCX) [file pgen.1009272.s006.docx]

**S1 Table. List of genomes downloaded.**

| MF598691_OrganismMiddle_East_respiratory_syndrome_related_coronavirus_Strain_Namecamel_UAE_B104_2015_Segmentnull_HostCamel.fa | B1 |
| --- | --- |
| MF598612_OrganismMiddle_East_respiratory_syndrome_related_coronavirus_Strain_Namecamel_UAE_B20_2015_Segmentnull_HostCamel.fa | B2 |
| MF598657_OrganismMiddle_East_respiratory_syndrome_related_coronavirus_Strain_Namecamel_UAE_B67_2015_Segmentnull_HostCamel.fa | B3 |
| MF598684_OrganismMiddle_East_respiratory_syndrome_related_coronavirus_Strain_Namecamel_UAE_B96_2015_Segmentnull_HostCamel.fa | B4 |
| MF598685_OrganismMiddle_East_respiratory_syndrome_related_coronavirus_Strain_Namecamel_UAE_B97_2015_Segmentnull_HostCamel.fa | B5 |
| MF598688_OrganismMiddle_East_respiratory_syndrome_related_coronavirus_Strain_Namecamel_UAE_B101_2015_Segmentnull_HostCamel.fa | B6 |
| KT225476_OrganismMiddle_East_respiratory_syndrome_related_coronavirus_Strain_NameMERS_CoV_THA_CU_17_06_2015_Segmentnull_HostHuman.fa | B7 |
| KT806044_OrganismMiddle_East_respiratory_syndrome_coronavirus_Strain_NameHu_Jeddah_KSA_C20843_2015_Segmentnull_HostHuman.fa | B8 |
| KT806045_OrganismMiddle_East_respiratory_syndrome_coronavirus_Strain_NameHu_Jeddah_KSA_C21271_2015_Segmentnull_HostHuman.fa | B9 |
| KT806054_OrganismMiddle_East_respiratory_syndrome_coronavirus_Strain_NameHu_Najran_KSA_C20915_2015_Segmentnull_HostHuman.fa | B10 |
| KT368878_OrganismMiddle_East_respiratory_syndrome_coronavirus_Strain_Namecamel_Riyadh_Ry79_2015_Segmentnull_HostCamel.fa | B11 |
| KT806047_OrganismMiddle_East_respiratory_syndrome_coronavirus_Strain_NameHu_Hufuf_KSA_9158_2015_Segmentnull_HostHuman.fa | B12 |
| KU851859_OrganismMiddle_East_respiratory_syndrome_coronavirus_Strain_NameHu_Jeddah_KSA_3RS2702_2015_Segmentnull_HostHuman.fa | B13 |
| KT368884_OrganismMiddle_East_respiratory_syndrome_coronavirus_Strain_Namecamel_Taif_T68_2015_Segmentnull_HostCamel.fa | B14 |
| KT368885_OrganismMiddle_East_respiratory_syndrome_coronavirus_Strain_Namecamel_Taif_T89_2015_Segmentnull_HostCamel.fa | B15 |
| KT368886_OrganismMiddle_East_respiratory_syndrome_coronavirus_Strain_Namecamel_Taif_T91 | B16 |
| KT368883_OrganismMiddle_East_respiratory_syndrome_coronavirus_Strain_Namecamel_Taif_T22_2015_Segmentnull_HostCamel.fa | B17 |
| KT368882_OrganismMiddle_East_respiratory_syndrome_coronavirus_Strain_Namecamel_Taif_T16_2015_Segmentnull_HostCamel.fa | B18 |
| KT368880_OrganismMiddle_East_respiratory_syndrome_coronavirus_Strain_Namecamel_Taif_T3_2015_Segmentnull_HostCamel.fa | B19 |
| KT368881_OrganismMiddle_East_respiratory_syndrome_coronavirus_Strain_Namecamel_Taif_T7_2015_Segmentnull_HostCamel.fa | B20 |
| KT368873_OrganismMiddle_East_respiratory_syndrome_coronavirus_Strain_Namecamel_Riyadh_Ry177_2015_Segmentnull_HostCamel.fa | B21 |
| KT368872_OrganismMiddle_East_respiratory_syndrome_coronavirus_Strain_Namecamel_Riyadh_Ry173_2015_Segmentnull_HostCamel.fa | B22 |
| KT368874_OrganismMiddle_East_respiratory_syndrome_coronavirus_Strain_Namecamel_Riyadh_Ry178_2015_Segmentnull_HostCamel.fa | B23 |
| MG757605_OrganismMiddle_East_respiratory_syndrome_related_coronavirus_Strain_NameHu_Riyadh_KSA_036D1N_2016_Segmentnull_HostHuman.fa | B24 |
| MH371127_OrganismMiddle_East_respiratory_syndrome_related_coronavirus_Strain_Name2362_Segmentnull_HostUnknown.fa | B25 |
| MG470650_OrganismMiddle_East_respiratory_syndrome_related_coronavirus_Strain_Name2362_Segmentnull_HostHuman.fa | B26 |
| MG011353_OrganismMiddle_East_respiratory_syndrome_related_coronavirus_Strain_NameHu_Riyadh_KSA_19949_2016_Segmentnull_HostHuman.fa | B27 |
| MH432120_OrganismMiddle_East_respiratory_syndrome_related_coronavirus_Strain_Name2366_Segmentnull_HostHuman.fa | B28 |
| MG011355_OrganismMiddle_East_respiratory_syndrome_related_coronavirus_Strain_NameHu_Riyadh_KSA_21155_2016_Segmentnull_HostHuman.fa | B29 |
| MK462244_OrganismMiddle_East_respiratory_syndrome_related_coronavirus_Strain_NameHu_Aseer_KSA_173RS1288_2017_Segmentnull_HostHuman.fa | B30 |
| MK462246_OrganismMiddle_East_respiratory_syndrome_related_coronavirus_Strain_NameHu_Jeddah_KSA_173RS1570_2017_Segmentnull_HostHuman.fa | B31 |
| MN723542_OrganismMiddle_East_respiratory_syndrome_related_coronavirus_Strain_NameHu_Jeddah_KSA_173RS1101_2017_Segmentnull_HostHuman.fa | B32 |
| MN723543_OrganismMiddle_East_respiratory_syndrome_related_coronavirus_Strain_NameHu_Jeddah_KSA_173RS1570_2017_Segmentnull_HostHuman.fa | B33 |
| MK462243_OrganismMiddle_East_respiratory_syndrome_related_coronavirus_Strain_NameHu_Jeddah_KSA_173RS1101_2017_Segmentnull_HostHuman.fa | B34 |
| MK462245_OrganismMiddle_East_respiratory_syndrome_related_coronavirus_Strain_NameHu_Jeddah_KSA_173RS1512_2017_Segmentnull_HostHuman.fa | B35 |
| MK462251_OrganismMiddle_East_respiratory_syndrome_related_coronavirus_Strain_NameHu_Northern_KSA_1847784_2018_Segmentnull_HostHuman.fa | B36 |
| MK462252_OrganismMiddle_East_respiratory_syndrome_related_coronavirus_Strain_NameHu_Tabuk_KSA_153_2018_Segmentnull_HostHuman.fa | B37 |
| MK462256_OrganismMiddle_East_respiratory_syndrome_related_coronavirus_Strain_NameHu_Riyadh_KSA_18014506_2018_Segmentnull_HostHuman.fa | B38 |
| MK462247_OrganismMiddle_East_respiratory_syndrome_related_coronavirus_Strain_NameHu_Jeddah_KSA_182RS2449_2018_Segmentnull_HostHuman.fa | B39 |
| MH822886_OrganismMiddle_East_respiratory_syndrome_related_coronavirus_Strain_NameMERS_CoV_England_KSA_1_2018_Segmentnull_HostHuman.fa | B40 |
| MK483839_OrganismMiddle_East_respiratory_syndrome_related_coronavirus_Strain_NameHu_Albaha_KSA_0800H_2018_Segmentnull_HostHuman.fa | B41 |
| MK462248_OrganismMiddle_East_respiratory_syndrome_related_coronavirus_Strain_NameHu_Najran_KSA_182RS2567_2018_Segmentnull_HostHuman.fa | B42 |
| MN365232_OrganismMiddle_East_respiratory_syndrome_related_coronavirus_Strain_NameHu_Riyadh_KSA_19001796_2019_Segmentnull_HostHuman.fa | B43 |
| MN365233_OrganismMiddle_East_respiratory_syndrome_related_coronavirus_Strain_NameHu_Riyadh_KSA_19003852_2019_Segmentnull_HostHuman.fa | B44 |
| MK462254_OrganismMiddle_East_respiratory_syndrome_related_coronavirus_Strain_NameHu_Qaseem_KSA_18013897_2018_Segmentnull_HostHuman.fa | B45 |
| MN120513_OrganismMiddle_East_respiratory_syndrome_related_coronavirus_Strain_Name016_Segmentnull_HostHuman.fa | B46 |
| MN120514_OrganismMiddle_East_respiratory_syndrome_related_coronavirus_Strain_Name013_Segmentnull_HostHuman.fa | B47 |
| MK462255_OrganismMiddle_East_respiratory_syndrome_related_coronavirus_Strain_NameHu_Riyadh_KSA_18014504_2018_Segmentnull_HostHuman.fa | B48 |
| MK462253_OrganismMiddle_East_respiratory_syndrome_related_coronavirus_Strain_NameHu_Riyadh_KSA_18013832_2018_Segmentnull_HostHuman.fa | B49 |
| MN723544_OrganismMiddle_East_respiratory_syndrome_related_coronavirus_Strain_NameHu_Riyadh_KSA_18013832_2018_Segmentnull_HostHuman.fa | B50 |
| MK462250_OrganismMiddle_East_respiratory_syndrome_related_coronavirus_Strain_NameHu_Najran_KSA_183RS279_2018_Segmentnull_HostHuman.fa | B51 |
| MK462249_OrganismMiddle_East_respiratory_syndrome_related_coronavirus_Strain_NameHu_Riyadh_KSA_18012493_2018_Segmentnull_HostHuman.fa | B52 |
| MH310912_OrganismMiddle_East_respiratory_syndrome_related_coronavirus_Strain_NameHu_Riyadh_KSA_7344_2017_Segmentnull_HostHuman.fa | B53 |
| MG011341_OrganismMiddle_East_respiratory_syndrome_related_coronavirus_Strain_NameHu_Qasim_KSA_13922_2016_Segmentnull_HostHuman.fa | B54 |
| MG011343_OrganismMiddle_East_respiratory_syndrome_related_coronavirus_Strain_NameHu_Riyadh_KSA_14607_2016_Segmentnull_HostHuman.fa | B55 |
| MG011352_OrganismMiddle_East_respiratory_syndrome_related_coronavirus_Strain_NameHu_Qasim_KSA_893_2016_Segmentnull_HostHuman.fa | B56 |
| KX154686_OrganismMiddle_East_respiratory_syndrome_related_coronavirus_Strain_NameHu_Riyadh_KSA_11898_2016_Segmentnull_HostHuman.fa | B57 |
| KX154692_OrganismMiddle_East_respiratory_syndrome_related_coronavirus_Strain_NameHu_Riyadh_KSA_12969_2016_Segmentnull_HostHuman.fa | B58 |
| KX154693_OrganismMiddle_East_respiratory_syndrome_related_coronavirus_Strain_NameHu_Riyadh_KSA_13127_2016_Segmentnull_HostHuman.fa | B59 |
| MG011354_OrganismMiddle_East_respiratory_syndrome_related_coronavirus_Strain_NameHu_Riyadh_KSA_20570_2016_Segmentnull_HostHuman.fa | B60 |
| MG912608_OrganismMiddle_East_respiratory_syndrome_related_coronavirus_Strain_NameHu_Riyadh_KSA_9730_2017_Segmentnull_HostHuman.fa | B61 |
| MG912607_OrganismMiddle_East_respiratory_syndrome_related_coronavirus_Strain_NameHu_Riyadh_KSA_9522_2017_Segmentnull_HostHuman.fa | B62 |
| MG366880_OrganismMiddle_East_respiratory_syndrome_related_coronavirus_Strain_NameHu_Riyadh_KSA_7436_2017_Segmentnull_HostHuman.fa | B63 |
| MK129253_OrganismMiddle_East_respiratory_syndrome_related_coronavirus_Strain_NameMERS_CoV_KOR_KCDC_001_2018_TSVi_Segmentnull_HostHuman.fa | B64 |
| MG011362_OrganismMiddle_East_respiratory_syndrome_related_coronavirus_Strain_NameHu_Riyadh_KSA_K37029157_2016_Segmentnull_HostHuman.fa | B65 |
| MG011361_OrganismMiddle_East_respiratory_syndrome_related_coronavirus_Strain_NameHu_Riyadh_KSA_K17000405_2017_Segmentnull_HostHuman.fa | B66 |
| MG011360_OrganismMiddle_East_respiratory_syndrome_related_coronavirus_Strain_NameHu_Riyadh_KSA_K17000887_2017_Segmentnull_HostHuman.fa | B67 |
| KX154684_OrganismMiddle_East_respiratory_syndrome_related_coronavirus_Strain_NameHu_Riyadh_KSA_11739_2016_Segmentnull_HostHuman.fa | B68 |
| MG011359_OrganismMiddle_East_respiratory_syndrome_related_coronavirus_Strain_NameHu_Riyadh_KSA_16559_2016_Segmentnull_HostHuman.fa | B69 |
| MH454272_OrganismMiddle_East_respiratory_syndrome_related_coronavirus_Strain_NameHCoV_EMC_Segmentnull_HostHuman.fa | B70 |
| MH395139_OrganismMiddle_East_respiratory_syndrome_related_coronavirus_Strain_Name2363_Segmentnull_HostHuman.fa | B71 |
| KX154694_OrganismMiddle_East_respiratory_syndrome_related_coronavirus_Strain_NameHu_Artawiyah_KSA_13328_2016_Segmentnull_HostHuman.fa | B72 |
| MH306207_OrganismMiddle_East_respiratory_syndrome_related_coronavirus_Strain_NameHCoV_EMC_Segmentnull_HostHuman.fa | B73 |
| MF000460_OrganismMiddle_East_respiratory_syndrome_related_coronavirus_Strain_NameHu_Amman_Jordan_12716_2015_Segmentnull_HostHuman.fa | B74 |
| MF000459_OrganismMiddle_East_respiratory_syndrome_related_coronavirus_Strain_NameHu_Amman_Jordan_12918_2015_Segmentnull_HostHuman.fa | B75 |
| MF000458_OrganismMiddle_East_respiratory_syndrome_related_coronavirus_Strain_NameHu_Amman_Jordan_13030_2015_Segmentnull_HostHuman.fa | B76 |
| MF000457_OrganismMiddle_East_respiratory_syndrome_related_coronavirus_Strain_NameHu_Amman_Jordan_12641_2015_Segmentnull_HostHuman.fa | B77 |
| MG757596_OrganismMiddle_East_respiratory_syndrome_related_coronavirus_Strain_NameHu_Riyadh_KSA_006D1S_2015_Segmentnull_HostHuman.fa | B78 |
| MG757594_OrganismMiddle_East_respiratory_syndrome_related_coronavirus_Strain_NameHu_Riyadh_KSA_004D1S_2015_Segmentnull_HostHuman.fa | B79 |
| KU851864_OrganismMiddle_East_respiratory_syndrome_coronavirus_Strain_NameHu_Riyadh_KSA_16098_2015_Segmentnull_HostHuman.fa | B80 |
| MG520076_OrganismMiddle_East_respiratory_syndrome_related_coronavirus_Strain_NameHu_Riyadh_KSA_023D3N_2015_Segmentnull_HostHuman.fa | B81 |
| MG520075_OrganismMiddle_East_respiratory_syndrome_related_coronavirus_Strain_NameHu_Riyadh_KSA_023D1N_2015_Segmentnull_HostHuman.fa | B82 |
| MG546331_OrganismMiddle_East_respiratory_syndrome_related_coronavirus_Strain_NameHu_Riyadh_KSA_023D3N_2015_Segmentnull_HostHuman.fa | B83 |
| MG546330_OrganismMiddle_East_respiratory_syndrome_related_coronavirus_Strain_NameHu_Riyadh_KSA_023D1N_2015_Segmentnull_HostHuman.fa | B84 |
| MG757595_OrganismMiddle_East_respiratory_syndrome_related_coronavirus_Strain_NameHu_Riyadh_KSA_005D1S_2015_Segmentnull_HostHuman.fa | B85 |
| MG757599_OrganismMiddle_East_respiratory_syndrome_related_coronavirus_Strain_NameHu_Riyadh_KSA_009D1N_2015_Segmentnull_HostHuman.fa | B86 |
| MG757602_OrganismMiddle_East_respiratory_syndrome_related_coronavirus_Strain_NameHu_Riyadh_KSA_015D1P_2015_Segmentnull_HostHuman.fa | B87 |
| KU851862_OrganismMiddle_East_respiratory_syndrome_coronavirus_Strain_NameHu_Riyadh_KSA_16117_2015_Segmentnull_HostHuman.fa | B88 |
| KU851861_OrganismMiddle_East_respiratory_syndrome_coronavirus_Strain_NameHu_Riyadh_KSA_16120_2015_Segmentnull_HostHuman.fa | B89 |
| MG757600_OrganismMiddle_East_respiratory_syndrome_related_coronavirus_Strain_NameHu_Riyadh_KSA_012D1S_2015_Segmentnull_HostHuman.fa | B90 |
| MG757603_OrganismMiddle_East_respiratory_syndrome_related_coronavirus_Strain_NameHu_Riyadh_KSA_031D1N_2015_Segmentnull_HostHuman.fa | B91 |
| MH029552_OrganismMiddle_East_respiratory_syndrome_related_coronavirus_Strain_NameHu_Riyadh_KSA_008D3N_2015_Segmentnull_HostHuman.fa | B92 |
| MG757598_OrganismMiddle_East_respiratory_syndrome_related_coronavirus_Strain_NameHu_Riyadh_KSA_008D3N_2015_Segmentnull_HostHuman.fa | B93 |
| KU851860_OrganismMiddle_East_respiratory_syndrome_coronavirus_Strain_NameHu_Riyadh_KSA_16121_2015_Segmentnull_HostHuman.fa | B94 |
| MG757593_OrganismMiddle_East_respiratory_syndrome_related_coronavirus_Strain_NameHu_Riyadh_KSA_002D1N_2015_Segmentnull_HostHuman.fa | B95 |
| KT368870_OrganismMiddle_East_respiratory_syndrome_coronavirus_Strain_Namecamel_Riyadh_Ry159 | B96 |
| KT806046_OrganismMiddle_East_respiratory_syndrome_coronavirus_Strain_NameHu_Hufuf_KSA_11002_2015_Segmentnull_HostHuman.fa | B97 |
| KY688121_OrganismMiddle_East_respiratory_syndrome_related_coronavirus_Strain_NameHu_Hufuf_KSA_11442_2015_Segmentnull_HostHuman.fa | B98 |
| KY688122_OrganismMiddle_East_respiratory_syndrome_related_coronavirus_Strain_NameHu_Hufuf_KSA_11767_2015_Segmentnull_HostHuman.fa | B99 |
| KY688120_OrganismMiddle_East_respiratory_syndrome_related_coronavirus_Strain_NameHu_Hufuf_KSA_11002_2015_Segmentnull_HostHuman.fa | B100 |
| KY688124_OrganismMiddle_East_respiratory_syndrome_related_coronavirus_Strain_NameHu_Hufuf_KSA_11401_2015_Segmentnull_HostHuman.fa | B101 |
| KX034096_OrganismMiddle_East_respiratory_syndrome_coronavirus_Strain_NameMERS_CoV_KOR_Seoul_077_2_2015_Segmentnull_HostHuman.fa | B102 |
| KX034100_OrganismMiddle_East_respiratory_syndrome_coronavirus_Strain_NameMERS_CoV_KOR_Seoul_177_3_2015_Segmentnull_HostHuman.fa | B103 |
| KT374055_OrganismMiddle_East_respiratory_syndrome_coronavirus_Strain_NameKOREA_Seoul_035_2_2015_Segmentnull_HostHuman.fa | B104 |
| KT374054_OrganismMiddle_East_respiratory_syndrome_coronavirus_Strain_NameKOREA_Seoul_035_1_2015_Segmentnull_HostHuman.fa | B105 |
| KU308549_OrganismMiddle_East_respiratory_syndrome_coronavirus_Strain_NameKorea_Seoul_SNU1_035_2015_Segmentnull_HostHuman.fa | B106 |
| KT374052_OrganismMiddle_East_respiratory_syndrome_coronavirus_Strain_NameKOREA_Seoul_014_1_2015_Segmentnull_HostHuman.fa | B107 |
| KT374053_OrganismMiddle_East_respiratory_syndrome_coronavirus_Strain_NameKOREA_Seoul_014_2_2015_Segmentnull_HostHuman.fa | B108 |
| KT374050_OrganismMiddle_East_respiratory_syndrome_coronavirus_Strain_NameKOREA_Seoul_163_2_2015_Segmentnull_HostHuman.fa | B109 |
| KT374051_OrganismMiddle_East_respiratory_syndrome_coronavirus_Strain_NameKOREA_Seoul_163_1_2015_Segmentnull_HostHuman.fa | B110 |
| KX034095_OrganismMiddle_East_respiratory_syndrome_coronavirus_Strain_NameMERS_CoV_KOR_Seoul_066_2015_Segmentnull_HostHuman.fa | B111 |
| KX034097_OrganismMiddle_East_respiratory_syndrome_coronavirus_Strain_NameMERS_CoV_KOR_Seoul_080_3_2015_Segmentnull_HostHuman.fa | B112 |
| KX034094_OrganismMiddle_East_respiratory_syndrome_coronavirus_Strain_NameMERS_CoV_KOR_Seoul_050_1_2015_Segmentnull_HostHuman.fa | B113 |
| KT374056_OrganismMiddle_East_respiratory_syndrome_coronavirus_Strain_NameKOREA_Seoul_168_1_2015_Segmentnull_HostHuman.fa | B114 |
| KT374057_OrganismMiddle_East_respiratory_syndrome_coronavirus_Strain_NameKOREA_Seoul_168_2_2015_Segmentnull_HostHuman.fa | B115 |
| KX034099_OrganismMiddle_East_respiratory_syndrome_coronavirus_Strain_NameMERS_CoV_KOR_Seoul_169_2015_Segmentnull_HostHuman.fa | B116 |
| KY688123_OrganismMiddle_East_respiratory_syndrome_related_coronavirus_Strain_NameHu_Hufuf_KSA_11150_2015_Segmentnull_HostHuman.fa | B117 |
| KT006149_OrganismMiddle_East_respiratory_syndrome_coronavirus_Strain_NameChinaGD01_Segmentnull_HostHuman.fa | B118 |
| KX034098_OrganismMiddle_East_respiratory_syndrome_coronavirus_Strain_NameMERS_CoV_KOR_Seoul_162_1_2015_Segmentnull_HostHuman.fa | B119 |
| MK796425_OrganismMiddle_East_respiratory_syndrome_related_coronavirus_Strain_NameKNIH_002_05_2015_Segmentnull_HostHuman.fa | B120 |
| KT029139_OrganismMiddle_East_respiratory_syndrome_coronavirus_Strain_NameMERS_CoV_KOR_KNIH_002_05_2015_Segmentnull_HostHuman.fa | B121 |
| KT326819_OrganismMiddle_East_respiratory_syndrome_related_coronavirus_Strain_NameMERS_CoV_KOR_KNIH_001_05_2015_Segmentnull_HostHuman.fa | B122 |
| KT368860_OrganismMiddle_East_respiratory_syndrome_coronavirus_Strain_Namecamel_Jeddah_Jd6 | B123 |
| KT368861_OrganismMiddle_East_respiratory_syndrome_coronavirus_Strain_Namecamel_Jeddah_Jd7_2015_Segmentnull_HostCamel.fa | B124 |
| KT368858_OrganismMiddle_East_respiratory_syndrome_coronavirus_Strain_Namecamel_Jeddah_Jd1 | B125 |
| KT368859_OrganismMiddle_East_respiratory_syndrome_coronavirus_Strain_Namecamel_Jeddah_Jd4_2015_Segmentnull_HostCamel.fa | B126 |
| KT368864_OrganismMiddle_East_respiratory_syndrome_coronavirus_Strain_Namecamel_Jeddah_Jd87_2015_Segmentnull_HostCamel.fa | B127 |
| KT368865_OrganismMiddle_East_respiratory_syndrome_coronavirus_Strain_Namecamel_Jeddah_Jd90_2015_Segmentnull_HostCamel.fa | B128 |
| KT368862_OrganismMiddle_East_respiratory_syndrome_coronavirus_Strain_Namecamel_Jeddah_Jd85_2015_Segmentnull_HostCamel.fa | B129 |
| KT368863_OrganismMiddle_East_respiratory_syndrome_coronavirus_Strain_Namecamel_Jeddah_Jd86_2015_Segmentnull_HostCamel.fa | B130 |
| KT368848_OrganismMiddle_East_respiratory_syndrome_coronavirus_Strain_Namecamel_Jeddah_N68 | B131 |
| KT368846_OrganismMiddle_East_respiratory_syndrome_coronavirus_Strain_Namecamel_Jeddah_N51_2014_Segmentnull_HostCamel.fa | B132 |
| KT368835_OrganismMiddle_East_respiratory_syndrome_coronavirus_Strain_Namecamel_Jeddah_D42_2014_Segmentnull_HostCamel.fa | B133 |
| KT368836_OrganismMiddle_East_respiratory_syndrome_coronavirus_Strain_Namecamel_Jeddah_D43 | B134 |
| KT368838_OrganismMiddle_East_respiratory_syndrome_coronavirus_Strain_Namecamel_Jeddah_D46 | B135 |
| KT368842_OrganismMiddle_East_respiratory_syndrome_coronavirus_Strain_Namecamel_Jeddah_D50 | B136 |
| KT368839_OrganismMiddle_East_respiratory_syndrome_coronavirus_Strain_Namecamel_Jeddah_D47_2014_Segmentnull_HostCamel.fa | B137 |
| KT368837_OrganismMiddle_East_respiratory_syndrome_coronavirus_Strain_Namecamel_Jeddah_D45_2014_Segmentnull_HostCamel.fa | B138 |
| KT368840_OrganismMiddle_East_respiratory_syndrome_coronavirus_Strain_Namecamel_Jeddah_D48_2014_Segmentnull_HostCamel.fa | B139 |
| KT368847_OrganismMiddle_East_respiratory_syndrome_coronavirus_Strain_Namecamel_Jeddah_N62 | B140 |
| KT368841_OrganismMiddle_East_respiratory_syndrome_coronavirus_Strain_Namecamel_Jeddah_D49_2014_Segmentnull_HostCamel.fa | B141 |
| KT806052_OrganismMiddle_East_respiratory_syndrome_coronavirus_Strain_NameHu_Kharj_KSA_2599_2015_Segmentnull_HostHuman.fa | B142 |
| KT026454_OrganismMiddle_East_respiratory_syndrome_coronavirus_Strain_NameHu_Riyadh_KSA_4050_2015_Segmentnull_HostHuman.fa | B143 |
| KT026456_OrganismMiddle_East_respiratory_syndrome_coronavirus_Strain_NameHu_Riyadh_KSA_4050_2015_Segmentnull_HostHuman.fa | B144 |
| KT368877_OrganismMiddle_East_respiratory_syndrome_coronavirus_Strain_Namecamel_Riyadh_Ry64_2015_Segmentnull_HostCamel.fa | B145 |
| KT368868_OrganismMiddle_East_respiratory_syndrome_coronavirus_Strain_Namecamel_Riyadh_Ry136_2015_Segmentnull_HostCamel.fa | B146 |
| KT368876_OrganismMiddle_East_respiratory_syndrome_coronavirus_Strain_Namecamel_Riyadh_Ry63_2015_Segmentnull_HostCamel.fa | B147 |
| KT368871_OrganismMiddle_East_respiratory_syndrome_coronavirus_Strain_Namecamel_Riyadh_Ry162_2015_Segmentnull_HostCamel.fa | B148 |
| MG011351_OrganismMiddle_East_respiratory_syndrome_related_coronavirus_Strain_NameHu_Madinah_KSA_390_2016_Segmentnull_HostHuman.fa | B149 |
| MG011357_OrganismMiddle_East_respiratory_syndrome_related_coronavirus_Strain_NameHu_Riyadh_KSA_24244_2016_Segmentnull_HostHuman.fa | B150 |
| MG011356_OrganismMiddle_East_respiratory_syndrome_related_coronavirus_Strain_NameHu_Riyadh_KSA_21891_2016_Segmentnull_HostHuman.fa | B151 |
| KX154687_OrganismMiddle_East_respiratory_syndrome_related_coronavirus_Strain_NameHu_Riyadh_KSA_11958_2016_Segmentnull_HostHuman.fa | B152 |
| MG011342_OrganismMiddle_East_respiratory_syndrome_related_coronavirus_Strain_NameHu_Riyadh_KSA_13984_2016_Segmentnull_HostHuman.fa | B153 |
| KU851863_OrganismMiddle_East_respiratory_syndrome_coronavirus_Strain_NameHu_Riyadh_KSA_16077_2015_Segmentnull_HostHuman.fa | B154 |
| MG757601_OrganismMiddle_East_respiratory_syndrome_related_coronavirus_Strain_NameHu_Riyadh_KSA_014D1N_2015_Segmentnull_HostHuman.fa | B155 |
| KT368833_OrganismMiddle_East_respiratory_syndrome_coronavirus_Strain_Namecamel_Jeddah_D38 | B156 |
| KT368889_OrganismMiddle_East_respiratory_syndrome_coronavirus_Strain_Namecamel_Taif_T150_2015_Segmentnull_HostCamel.fa | B157 |
| KT368834_OrganismMiddle_East_respiratory_syndrome_coronavirus_Strain_Namecamel_Jeddah_D40_2014_Segmentnull_HostCamel.fa | B158 |
| MF598686_OrganismMiddle_East_respiratory_syndrome_related_coronavirus_Strain_Namecamel_UAE_B99_2015_Segmentnull_HostCamel.fa | B159 |
| KT368826_OrganismMiddle_East_respiratory_syndrome_coronavirus_Strain_Namecamel_Riyadh_Ry84N_2014_Segmentnull_HostCamel.fa | B160 |
| KT806053_OrganismMiddle_East_respiratory_syndrome_coronavirus_Strain_NameHu_Kharj_KSA_2598_2015_Segmentnull_HostHuman.fa | B161 |
| KT806051_OrganismMiddle_East_respiratory_syndrome_coronavirus_Strain_NameHu_Riyadh_KSA_2716_2015_Segmentnull_HostHuman.fa | B162 |
| MF598617_OrganismMiddle_East_respiratory_syndrome_related_coronavirus_Strain_Namecamel_UAE_B25_2015_Segmentnull_HostCamel.fa | B163 |
| MG757604_OrganismMiddle_East_respiratory_syndrome_related_coronavirus_Strain_NameHu_Riyadh_KSA_039D3T_2016_Segmentnull_HostHuman.fa | B164 |
| KX154689_OrganismMiddle_East_respiratory_syndrome_related_coronavirus_Strain_NameHu_Riyadh_KSA_10208_2016_Segmentnull_HostHuman.fa | B165 |
| MG011348_OrganismMiddle_East_respiratory_syndrome_related_coronavirus_Strain_NameHu_Riyadh_KSA_17756_2016_Segmentnull_HostHuman.fa | B166 |
| MG011349_OrganismMiddle_East_respiratory_syndrome_related_coronavirus_Strain_NameHu_Riyadh_KSA_17382_2016_Segmentnull_HostHuman.fa | B167 |
| MG011350_OrganismMiddle_East_respiratory_syndrome_related_coronavirus_Strain_NameHu_Riyadh_KSA_16849_2016_Segmentnull_HostHuman.fa | B168 |
| KT026455_OrganismMiddle_East_respiratory_syndrome_coronavirus_Strain_NameHu_Riyadh_KSA_2959_2015_Segmentnull_HostHuman.fa | B169 |
| KT026453_OrganismMiddle_East_respiratory_syndrome_coronavirus_Strain_NameHu_Riyadh_KSA_2959_2015_Segmentnull_HostHuman.fa | B170 |
| MG011340_OrganismMiddle_East_respiratory_syndrome_related_coronavirus_Strain_NameHu_Riyadh_KSA_13798_2016_Segmentnull_HostHuman.fa | B171 |
| KX154691_OrganismMiddle_East_respiratory_syndrome_related_coronavirus_Strain_NameHu_Riyadh_KSA_12832_2016_Segmentnull_HostHuman.fa | B172 |
| KX154688_OrganismMiddle_East_respiratory_syndrome_related_coronavirus_Strain_NameHu_Riyadh_KSA_12160_2016_Segmentnull_HostHuman.fa | B173 |
| KX154685_OrganismMiddle_East_respiratory_syndrome_related_coronavirus_Strain_NameHu_Riyadh_KSA_11740_2016_Segmentnull_HostHuman.fa | B174 |
| MG011346_OrganismMiddle_East_respiratory_syndrome_related_coronavirus_Strain_NameHu_Riyadh_KSA_14949_2016_Segmentnull_HostHuman.fa | B175 |
| MG011344_OrganismMiddle_East_respiratory_syndrome_related_coronavirus_Strain_NameHu_Riyadh_KSA_14675_2016_Segmentnull_HostHuman.fa | B176 |
| KT806049_OrganismMiddle_East_respiratory_syndrome_coronavirus_Strain_NameHu_Riyadh_KSA_3181_2015_Segmentnull_HostHuman.fa | B177 |
| MG011347_OrganismMiddle_East_respiratory_syndrome_related_coronavirus_Strain_NameHu_Riyadh_KSA_14670_2016_Segmentnull_HostHuman.fa | B178 |
| KX154690_OrganismMiddle_East_respiratory_syndrome_related_coronavirus_Strain_NameHu_Jeddah_KSA_161RS1146_2016_Segmentnull_HostHuman.fa | B179 |
| MG011345_OrganismMiddle_East_respiratory_syndrome_related_coronavirus_Strain_NameHu_Riyadh_KSA_15385_2016_Segmentnull_HostHuman.fa | B180 |
| KT368879_OrganismMiddle_East_respiratory_syndrome_coronavirus_Strain_Namecamel_Riyadh_Ry86_2015_Segmentnull_HostCamel.fa | B181 |
| KT368869_OrganismMiddle_East_respiratory_syndrome_coronavirus_Strain_Namecamel_Riyadh_Ry137_2015_Segmentnull_HostCamel.fa | B182 |
| MG366881_OrganismMiddle_East_respiratory_syndrome_related_coronavirus_Strain_NameHu_Riyadh_KSA_8677_2017_Segmentnull_HostHuman.fa | B183 |
| MG912606_OrganismMiddle_East_respiratory_syndrome_related_coronavirus_Strain_NameHu_Riyadh_KSA_9852_2017_Segmentnull_HostHuman.fa | B184 |
| MG912605_OrganismMiddle_East_respiratory_syndrome_related_coronavirus_Strain_NameHu_Riyadh_KSA_9835_2017_Segmentnull_HostHuman.fa | B185 |
| MG912597_OrganismMiddle_East_respiratory_syndrome_related_coronavirus_Strain_NameHu_Riyadh_KSA_7423_2017_Segmentnull_HostHuman.fa | B186 |
| MG912595_OrganismMiddle_East_respiratory_syndrome_related_coronavirus_Strain_NameHu_Riyadh_KSA_7178_2017_Segmentnull_HostHuman.fa | B187 |
| MH310910_OrganismMiddle_East_respiratory_syndrome_related_coronavirus_Strain_NameHu_Riyadh_KSA_7696_2017_Segmentnull_HostHuman.fa | B188 |
| MH310911_OrganismMiddle_East_respiratory_syndrome_related_coronavirus_Strain_NameHu_Riyadh_KSA_10717_2017_Segmentnull_HostHuman.fa | B189 |
| MG912600_OrganismMiddle_East_respiratory_syndrome_related_coronavirus_Strain_NameHu_Riyadh_KSA_8882_2017_Segmentnull_HostHuman.fa | B190 |
| MG912598_OrganismMiddle_East_respiratory_syndrome_related_coronavirus_Strain_NameHu_Riyadh_KSA_7680_2017_Segmentnull_HostHuman.fa | B191 |
| MG912596_OrganismMiddle_East_respiratory_syndrome_related_coronavirus_Strain_NameHu_Riyadh_KSA_7373_2017_Segmentnull_HostHuman.fa | B192 |
| MG366483_OrganismMiddle_East_respiratory_syndrome_related_coronavirus_Strain_NameHu_Riyadh_KSA_7413_2017_Segmentnull_HostHuman.fa | B193 |
| MG912601_OrganismMiddle_East_respiratory_syndrome_related_coronavirus_Strain_NameHu_Riyadh_KSA_10308_2017_Segmentnull_HostHuman.fa | B194 |
| MG366883_OrganismMiddle_East_respiratory_syndrome_related_coronavirus_Strain_NameHu_Riyadh_KSA_10024_2017_Segmentnull_HostHuman.fa | B195 |
| MG912599_OrganismMiddle_East_respiratory_syndrome_related_coronavirus_Strain_NameHu_Riyadh_KSA_8667_2017_Segmentnull_HostHuman.fa | B196 |
| MG912602_OrganismMiddle_East_respiratory_syndrome_related_coronavirus_Strain_NameHu_Riyadh_KSA_9614_2017_Segmentnull_HostHuman.fa | B197 |
| MG912604_OrganismMiddle_East_respiratory_syndrome_related_coronavirus_Strain_NameHu_Riyadh_KSA_9689_2017_Segmentnull_HostHuman.fa | B198 |
| MG366882_OrganismMiddle_East_respiratory_syndrome_related_coronavirus_Strain_NameHu_Riyadh_KSA_8683_2017_Segmentnull_HostHuman.fa | B199 |
| MG912603_OrganismMiddle_East_respiratory_syndrome_related_coronavirus_Strain_NameHu_Riyadh_KSA_9693_2017_Segmentnull_HostHuman.fa | B200 |
| MH310909_OrganismMiddle_East_respiratory_syndrome_related_coronavirus_Strain_NameHu_Riyadh_KSA_5767_2017_Segmentnull_HostHuman.fa | B201 |
| MG011358_OrganismMiddle_East_respiratory_syndrome_related_coronavirus_Strain_NameHu_Riyadh_KSA_24241_2016_Segmentnull_HostHuman.fa | B202 |
| MG757597_OrganismMiddle_East_respiratory_syndrome_related_coronavirus_Strain_NameHu_Riyadh_KSA_007D1N_2015_Segmentnull_HostHuman.fa | B203 |
| MF598680_OrganismMiddle_East_respiratory_syndrome_related_coronavirus_Strain_Namecamel_UAE_B91_2015_Segmentnull_HostCamel.fa | B204 |
| MF598604_OrganismMiddle_East_respiratory_syndrome_related_coronavirus_Strain_Namecamel_UAE_B12_2015_Segmentnull_HostCamel.fa | B205 |
| KX108946_OrganismMiddle_East_respiratory_syndrome_coronavirus_Strain_NameD1189.1_15_Segmentnull_HostCamel.fa | B206 |
| MF598602_OrganismMiddle_East_respiratory_syndrome_related_coronavirus_Strain_Namecamel_UAE_B10_2015_Segmentnull_HostCamel.fa | B207 |
| MF598618_OrganismMiddle_East_respiratory_syndrome_related_coronavirus_Strain_Namecamel_UAE_B26_2015_Segmentnull_HostCamel.fa | B208 |
| MF598595_OrganismMiddle_East_respiratory_syndrome_related_coronavirus_Strain_Namecamel_UAE_B2_2015_Segmentnull_HostCamel.fa | B209 |
| MF598660_OrganismMiddle_East_respiratory_syndrome_related_coronavirus_Strain_Namecamel_UAE_B70_2015_Segmentnull_HostCamel.fa | B210 |
| MF598643_OrganismMiddle_East_respiratory_syndrome_related_coronavirus_Strain_Namecamel_UAE_B52_2015_Segmentnull_HostCamel.fa | B211 |
| MF598627_OrganismMiddle_East_respiratory_syndrome_related_coronavirus_Strain_Namecamel_UAE_B35_2015_Segmentnull_HostCamel.fa | B212 |
| MF598625_OrganismMiddle_East_respiratory_syndrome_related_coronavirus_Strain_Namecamel_UAE_B33_2015_Segmentnull_HostCamel.fa | B213 |
| MF598615_OrganismMiddle_East_respiratory_syndrome_related_coronavirus_Strain_Namecamel_UAE_B23_2015_Segmentnull_HostCamel.fa | B214 |
| MF598701_OrganismMiddle_East_respiratory_syndrome_related_coronavirus_Strain_Namecamel_UAE_414498_W3_2015_Segmentnull_HostCamel.fa | B215 |
| MF598622_OrganismMiddle_East_respiratory_syndrome_related_coronavirus_Strain_Namecamel_UAE_B30_2015_Segmentnull_HostCamel.fa | B216 |
| MF598613_OrganismMiddle_East_respiratory_syndrome_related_coronavirus_Strain_Namecamel_UAE_B21_2015_Segmentnull_HostCamel.fa | B217 |
| MF598607_OrganismMiddle_East_respiratory_syndrome_related_coronavirus_Strain_Namecamel_UAE_B15_2015_Segmentnull_HostCamel.fa | B218 |
| MF598611_OrganismMiddle_East_respiratory_syndrome_related_coronavirus_Strain_Namecamel_UAE_B19_2015_Segmentnull_HostCamel.fa | B219 |
| KT806048_OrganismMiddle_East_respiratory_syndrome_coronavirus_Strain_NameHu_Khobar_KSA_6736_2015_Segmentnull_HostHuman.fa | B220 |
| KY688118_OrganismMiddle_East_respiratory_syndrome_related_coronavirus_Strain_NameHu_Khobar_KSA_6736_2015_Segmentnull_HostHuman.fa | B221 |
| MF598666_OrganismMiddle_East_respiratory_syndrome_related_coronavirus_Strain_Namecamel_UAE_B76_2015_Segmentnull_HostCamel.fa | B222 |
| MF598631_OrganismMiddle_East_respiratory_syndrome_related_coronavirus_Strain_Namecamel_UAE_B39_2015_Segmentnull_HostCamel.fa | B223 |
| MF598632_OrganismMiddle_East_respiratory_syndrome_related_coronavirus_Strain_Namecamel_UAE_B40_2015_Segmentnull_HostCamel.fa | B224 |
| MF598682_OrganismMiddle_East_respiratory_syndrome_related_coronavirus_Strain_Namecamel_UAE_B93_2015_Segmentnull_HostCamel.fa | B225 |
| MF598606_OrganismMiddle_East_respiratory_syndrome_related_coronavirus_Strain_Namecamel_UAE_B14_2015_Segmentnull_HostCamel.fa | B226 |
| MF598646_OrganismMiddle_East_respiratory_syndrome_related_coronavirus_Strain_Namecamel_UAE_B55_2015_Segmentnull_HostCamel.fa | B227 |
| MF598664_OrganismMiddle_East_respiratory_syndrome_related_coronavirus_Strain_Namecamel_UAE_B74_2015_Segmentnull_HostCamel.fa | B228 |
| MF598616_OrganismMiddle_East_respiratory_syndrome_related_coronavirus_Strain_Namecamel_UAE_B24_2015_Segmentnull_HostCamel.fa | B229 |
| MF598620_OrganismMiddle_East_respiratory_syndrome_related_coronavirus_Strain_Namecamel_UAE_B28_2015_Segmentnull_HostCamel.fa | B230 |
| MF598674_OrganismMiddle_East_respiratory_syndrome_related_coronavirus_Strain_Namecamel_UAE_B84_2015_Segmentnull_HostCamel.fa | B231 |
| MF598683_OrganismMiddle_East_respiratory_syndrome_related_coronavirus_Strain_Namecamel_UAE_B95_2015_Segmentnull_HostCamel.fa | B232 |
| MF598707_OrganismMiddle_East_respiratory_syndrome_related_coronavirus_Strain_Namecamel_UAE_415915_W3_2015_Segmentnull_HostCamel.fa | B233 |
| MF598708_OrganismMiddle_East_respiratory_syndrome_related_coronavirus_Strain_Namecamel_UAE_414492_W3_2015_Segmentnull_HostCamel.fa | B234 |
| MF598702_OrganismMiddle_East_respiratory_syndrome_related_coronavirus_Strain_Namecamel_UAE_414377_W3_2015_Segmentnull_HostCamel.fa | B235 |
| MF598712_OrganismMiddle_East_respiratory_syndrome_related_coronavirus_Strain_Namecamel_UAE_414492_W4_2015_Segmentnull_HostCamel.fa | B236 |
| MF598609_OrganismMiddle_East_respiratory_syndrome_related_coronavirus_Strain_Namecamel_UAE_B17_2015_Segmentnull_HostCamel.fa | B237 |
| MF598714_OrganismMiddle_East_respiratory_syndrome_related_coronavirus_Strain_Namecamel_UAE_414500_W4_2015_Segmentnull_HostCamel.fa | B238 |
| MF598709_OrganismMiddle_East_respiratory_syndrome_related_coronavirus_Strain_Namecamel_UAE_414485_W4_2015_Segmentnull_HostCamel.fa | B239 |
| MF598711_OrganismMiddle_East_respiratory_syndrome_related_coronavirus_Strain_Namecamel_UAE_414481_W4_2015_Segmentnull_HostCamel.fa | B240 |
| MF598671_OrganismMiddle_East_respiratory_syndrome_related_coronavirus_Strain_Namecamel_UAE_B81_2015_Segmentnull_HostCamel.fa | B241 |
| MF598690_OrganismMiddle_East_respiratory_syndrome_related_coronavirus_Strain_Namecamel_UAE_B103_2015_Segmentnull_HostCamel.fa | B242 |
| MF598659_OrganismMiddle_East_respiratory_syndrome_related_coronavirus_Strain_Namecamel_UAE_B69_2015_Segmentnull_HostCamel.fa | B243 |
| MF598634_OrganismMiddle_East_respiratory_syndrome_related_coronavirus_Strain_Namecamel_UAE_B42_2015_Segmentnull_HostCamel.fa | B244 |
| MF598623_OrganismMiddle_East_respiratory_syndrome_related_coronavirus_Strain_Namecamel_UAE_B31_2015_Segmentnull_HostCamel.fa | B245 |
| MF598614_OrganismMiddle_East_respiratory_syndrome_related_coronavirus_Strain_Namecamel_UAE_B22_2015_Segmentnull_HostCamel.fa | B246 |
| KT751244_OrganismMiddle_East_respiratory_syndrome_coronavirus_Strain_NameD2731.3_14_Segmentnull_HostCamel.fa | B247 |
| MF598673_OrganismMiddle_East_respiratory_syndrome_related_coronavirus_Strain_Namecamel_UAE_B83_2015_Segmentnull_HostCamel.fa | B248 |
| MF598626_OrganismMiddle_East_respiratory_syndrome_related_coronavirus_Strain_Namecamel_UAE_B34_2015_Segmentnull_HostCamel.fa | B249 |
| MF598598_OrganismMiddle_East_respiratory_syndrome_related_coronavirus_Strain_Namecamel_UAE_B6_2015_Segmentnull_HostCamel.fa | B250 |
| MF598655_OrganismMiddle_East_respiratory_syndrome_related_coronavirus_Strain_Namecamel_UAE_B65_2015_Segmentnull_HostCamel.fa | B251 |
| MF598605_OrganismMiddle_East_respiratory_syndrome_related_coronavirus_Strain_Namecamel_UAE_B13_2015_Segmentnull_HostCamel.fa | B252 |
| MF598722_OrganismMiddle_East_respiratory_syndrome_related_coronavirus_Strain_Namecamel_UAE_415915_W6_2015_Segmentnull_HostCamel.fa | B253 |
| MF598721_OrganismMiddle_East_respiratory_syndrome_related_coronavirus_Strain_Namecamel_UAE_416452_W6_2015_Segmentnull_HostCamel.fa | B254 |
| MF598720_OrganismMiddle_East_respiratory_syndrome_related_coronavirus_Strain_Namecamel_UAE_416452_W5_2015_Segmentnull_HostCamel.fa | B255 |
| MF598715_OrganismMiddle_East_respiratory_syndrome_related_coronavirus_Strain_Namecamel_UAE_416452_W4_2015_Segmentnull_HostCamel.fa | B256 |
| MF598719_OrganismMiddle_East_respiratory_syndrome_related_coronavirus_Strain_Namecamel_UAE_415911_W4_2015_Segmentnull_HostCamel.fa | B257 |
| MF598600_OrganismMiddle_East_respiratory_syndrome_related_coronavirus_Strain_Namecamel_UAE_B8_2015_Segmentnull_HostCamel.fa | B258 |
| MF598610_OrganismMiddle_East_respiratory_syndrome_related_coronavirus_Strain_Namecamel_UAE_B18_2015_Segmentnull_HostCamel.fa | B259 |
| MF598599_OrganismMiddle_East_respiratory_syndrome_related_coronavirus_Strain_Namecamel_UAE_B7_2015_Segmentnull_HostCamel.fa | B260 |
| MF598596_OrganismMiddle_East_respiratory_syndrome_related_coronavirus_Strain_Namecamel_UAE_B4_2015_Segmentnull_HostCamel.fa | B261 |
| MF598601_OrganismMiddle_East_respiratory_syndrome_related_coronavirus_Strain_Namecamel_UAE_B9_2015_Segmentnull_HostCamel.fa | B262 |
| MF598717_OrganismMiddle_East_respiratory_syndrome_related_coronavirus_Strain_Namecamel_UAE_417163_W4_2015_Segmentnull_HostCamel.fa | B263 |
| MF598704_OrganismMiddle_East_respiratory_syndrome_related_coronavirus_Strain_Namecamel_UAE_414500_W3_2015_Segmentnull_HostCamel.fa | B264 |
| MF598703_OrganismMiddle_East_respiratory_syndrome_related_coronavirus_Strain_Namecamel_UAE_414480_W3_2015_Segmentnull_HostCamel.fa | B265 |
| MF598706_OrganismMiddle_East_respiratory_syndrome_related_coronavirus_Strain_Namecamel_UAE_415911_W3_2015_Segmentnull_HostCamel.fa | B266 |
| MF598713_OrganismMiddle_East_respiratory_syndrome_related_coronavirus_Strain_Namecamel_UAE_414377_W4_2015_Segmentnull_HostCamel.fa | B267 |
| MF598699_OrganismMiddle_East_respiratory_syndrome_related_coronavirus_Strain_Namecamel_UAE_414485_W3_2015_Segmentnull_HostCamel.fa | B268 |
| MF598716_OrganismMiddle_East_respiratory_syndrome_related_coronavirus_Strain_Namecamel_UAE_414486_W4_2015_Segmentnull_HostCamel.fa | B269 |
| MF598710_OrganismMiddle_East_respiratory_syndrome_related_coronavirus_Strain_Namecamel_UAE_417162_W4_2015_Segmentnull_HostCamel.fa | B270 |
| MF598705_OrganismMiddle_East_respiratory_syndrome_related_coronavirus_Strain_Namecamel_UAE_414486_W3_2015_Segmentnull_HostCamel.fa | B271 |
| MF598630_OrganismMiddle_East_respiratory_syndrome_related_coronavirus_Strain_Namecamel_UAE_B38_2015_Segmentnull_HostCamel.fa | B272 |
| MF598640_OrganismMiddle_East_respiratory_syndrome_related_coronavirus_Strain_Namecamel_UAE_B49_2015_Segmentnull_HostCamel.fa | B273 |
| MF598700_OrganismMiddle_East_respiratory_syndrome_related_coronavirus_Strain_Namecamel_UAE_414379_W3_2015_Segmentnull_HostCamel.fa | B274 |
| MF598653_OrganismMiddle_East_respiratory_syndrome_related_coronavirus_Strain_Namecamel_UAE_B63_2015_Segmentnull_HostCamel.fa | B275 |
| MF598629_OrganismMiddle_East_respiratory_syndrome_related_coronavirus_Strain_Namecamel_UAE_B37_2015_Segmentnull_HostCamel.fa | B276 |
| MF598689_OrganismMiddle_East_respiratory_syndrome_related_coronavirus_Strain_Namecamel_UAE_B102_2015_Segmentnull_HostCamel.fa | B277 |
| MF598608_OrganismMiddle_East_respiratory_syndrome_related_coronavirus_Strain_Namecamel_UAE_B16_2015_Segmentnull_HostCamel.fa | B278 |
| KT368887_OrganismMiddle_East_respiratory_syndrome_coronavirus_Strain_Namecamel_Taif_T92_2015_Segmentnull_HostCamel.fa | B279 |
| KT368825_OrganismMiddle_East_respiratory_syndrome_coronavirus_Strain_Namecamel_Riyadh_Ry23N_2014_Segmentnull_HostCamel.fa | B280 |
| KT368855_OrganismMiddle_East_respiratory_syndrome_coronavirus_Strain_Namecamel_Jeddah_S93_2014_Segmentnull_HostCamel.fa | B281 |
| KT368854_OrganismMiddle_East_respiratory_syndrome_coronavirus_Strain_Namecamel_Jeddah_S73_2014_Segmentnull_HostCamel.fa | B282 |
| KT368853_OrganismMiddle_East_respiratory_syndrome_coronavirus_Strain_Namecamel_Jeddah_S100_2014_Segmentnull_HostCamel.fa | B283 |
| KT368851_OrganismMiddle_East_respiratory_syndrome_coronavirus_Strain_Namecamel_Jeddah_O30_2014_Segmentnull_HostCamel.fa | B284 |
| KT368850_OrganismMiddle_East_respiratory_syndrome_coronavirus_Strain_Namecamel_Jeddah_O24_2014_Segmentnull_HostCamel.fa | B285 |
| KT368849_OrganismMiddle_East_respiratory_syndrome_coronavirus_Strain_Namecamel_Jeddah_O23 | B286 |
| KT368843_OrganismMiddle_East_respiratory_syndrome_coronavirus_Strain_Namecamel_Jeddah_D88_2014_Segmentnull_HostCamel.fa | B287 |
| KT368845_OrganismMiddle_East_respiratory_syndrome_coronavirus_Strain_Namecamel_Jeddah_D92_2014_Segmentnull_HostCamel.fa | B288 |
| KT368828_OrganismMiddle_East_respiratory_syndrome_coronavirus_Strain_Namecamel_Jeddah_D100_2014_Segmentnull_HostCamel.fa | B289 |
| KT368844_OrganismMiddle_East_respiratory_syndrome_coronavirus_Strain_Namecamel_Jeddah_D90_2014_Segmentnull_HostCamel.fa | B290 |
| KT368852_OrganismMiddle_East_respiratory_syndrome_coronavirus_Strain_Namecamel_Jeddah_O47 | B291 |
| KT368857_OrganismMiddle_East_respiratory_syndrome_coronavirus_Strain_Namecamel_Jeddah_S99_2014_Segmentnull_HostCamel.fa | B292 |
| KT368856_OrganismMiddle_East_respiratory_syndrome_coronavirus_Strain_Namecamel_Jeddah_S94_2014_Segmentnull_HostCamel.fa | B293 |
| KT368827_OrganismMiddle_East_respiratory_syndrome_coronavirus_Strain_Namecamel_Jeddah_401_2014_Segmentnull_HostCamel.fa | B294 |
| KT368890_OrganismMiddle_East_respiratory_syndrome_coronavirus_Strain_Namecamel_Taif_T157 | B295 |
| KT806055_OrganismMiddle_East_respiratory_syndrome_coronavirus_Strain_NameHu_Jeddah_KSA_C20860_2015_Segmentnull_HostHuman.fa | B296 |
| KT368866_OrganismMiddle_East_respiratory_syndrome_coronavirus_Strain_Namecamel_Jeddah_Jd175_2015_Segmentnull_HostCamel.fa | B297 |
| KT368867_OrganismMiddle_East_respiratory_syndrome_coronavirus_Strain_Namecamel_Jeddah_Jd199_2015_Segmentnull_HostCamel.fa | B298 |
| KU710264_OrganismMiddle_East_respiratory_syndrome_coronavirus_Strain_NameHu_Taif_KSA_7032_2014_Segmentnull_HostHuman.fa | B299 |
| KU710265_OrganismMiddle_East_respiratory_syndrome_coronavirus_Strain_NameHu_Taif_KSA_7032_2014_S530del_Segmentnull_HostHuman.fa | B300 |
| KJ156934_OrganismMiddle_East_respiratory_syndrome_coronavirus_Strain_NameRiyadh_14_2013_Segmentnull_HostHuman.fa | B301 |
| KF600628_OrganismMiddle_East_respiratory_syndrome_coronavirus_Strain_NameHafr_Al_Batin_1_2013_Segmentnull_HostHuman.fa | B302 |
| KF961221_OrganismMiddle_East_respiratory_syndrome_coronavirus_Strain_NameQatar3_Segmentnull_HostHuman.fa | B303 |
| KF961222_OrganismMiddle_East_respiratory_syndrome_coronavirus_Strain_NameQatar4_Segmentnull_HostHuman.fa | B304 |
| KT368888_OrganismMiddle_East_respiratory_syndrome_coronavirus_Strain_Namecamel_Taif_T98_2015_Segmentnull_HostCamel.fa | B305 |
| MF598668_OrganismMiddle_East_respiratory_syndrome_related_coronavirus_Strain_Namecamel_UAE_B78_2015_Segmentnull_HostCamel.fa | B306 |
| MF598670_OrganismMiddle_East_respiratory_syndrome_related_coronavirus_Strain_Namecamel_UAE_B80_2015_Segmentnull_HostCamel.fa | B307 |
| MF598677_OrganismMiddle_East_respiratory_syndrome_related_coronavirus_Strain_Namecamel_UAE_B87_2015_Segmentnull_HostCamel.fa | B308 |
| MF598652_OrganismMiddle_East_respiratory_syndrome_related_coronavirus_Strain_Namecamel_UAE_B62_2015_Segmentnull_HostCamel.fa | B309 |
| MF598650_OrganismMiddle_East_respiratory_syndrome_related_coronavirus_Strain_Namecamel_UAE_B60_2015_Segmentnull_HostCamel.fa | B310 |
| MF598649_OrganismMiddle_East_respiratory_syndrome_related_coronavirus_Strain_Namecamel_UAE_B59_2015_Segmentnull_HostCamel.fa | B311 |
| MF598656_OrganismMiddle_East_respiratory_syndrome_related_coronavirus_Strain_Namecamel_UAE_B66_2015_Segmentnull_HostCamel.fa | B312 |
| MF598651_OrganismMiddle_East_respiratory_syndrome_related_coronavirus_Strain_Namecamel_UAE_B61_2015_Segmentnull_HostCamel.fa | B313 |
| MF598696_OrganismMiddle_East_respiratory_syndrome_related_coronavirus_Strain_Namecamel_UAE_B109_2015_Segmentnull_HostCamel.fa | B314 |
| MF598638_OrganismMiddle_East_respiratory_syndrome_related_coronavirus_Strain_Namecamel_UAE_B47_2015_Segmentnull_HostCamel.fa | B315 |
| MF598597_OrganismMiddle_East_respiratory_syndrome_related_coronavirus_Strain_Namecamel_UAE_B5_2015_Segmentnull_HostCamel.fa | B316 |
| MF598645_OrganismMiddle_East_respiratory_syndrome_related_coronavirus_Strain_Namecamel_UAE_B54_2015_Segmentnull_HostCamel.fa | B317 |
| MF598641_OrganismMiddle_East_respiratory_syndrome_related_coronavirus_Strain_Namecamel_UAE_B50_2015_Segmentnull_HostCamel.fa | B318 |
| KJ713298_OrganismMiddle_East_respiratory_syndrome_coronavirus_Strain_NameKSA_CAMEL_363_Segmentnull_HostCamel.fa | B319 |
| KF192507_OrganismMiddle_East_respiratory_syndrome_coronavirus_Strain_NameMunich_Segmentnull_HostHuman.fa | B320 |
| NC_009020_OrganismPipistrellus_bat_coronavirus_HKU5_Strain_NameHKU5_1_LMH03f_Segmentnull_HostBat.fa | B321 |
| EF065509_OrganismBat_coronavirus_HKU5_1_Strain_NameLMH03f_Segmentnull_HostBat.fa | B322 |
| MH002342_OrganismPipistrellus_bat_coronavirus_HKU5_Strain_NameYD13403_Segmentnull_HostUnknown.fa | B323 |
| KJ473820_OrganismBtPa_BetaCoV_GD2013_Strain_NameBtPa_GD2013_Segmentnull_HostBat.fa | B324 |
| MH002341_OrganismPipistrellus_bat_coronavirus_HKU5_Strain_NameBY140562_Segmentnull_HostUnknown.fa | B325 |
| EF065510_OrganismBat_coronavirus_HKU5_2_Strain_NameTT03f_Segmentnull_HostBat.fa | B326 |
| EF065511_OrganismBat_coronavirus_HKU5_3_Strain_NameTT06f_Segmentnull_HostBat.fa | B327 |
| EF065512_OrganismBat_coronavirus_HKU5_5_Strain_NameTT07f_Segmentnull_HostBat.fa | B328 |
| MN611520_OrganismPipistrellus_abramus_bat_coronavirus_HKU5_related_Strain_NameBY140568_Segmentnull_HostBat.fa | B329 |
| MH002340_OrganismPipistrellus_bat_coronavirus_HKU5_Strain_NameBY140535_Segmentnull_HostUnknown.fa | B330 |
| DQ648794_OrganismBat_coronavirus_ | B331 |
| MN611519_OrganismTylonycteris_pachypus_bat_coronavirus_HKU4_related_Strain_NameGZ131656_Segmentnull_HostBat.fa | B332 |
| MH002339_OrganismTylonycteris_bat_coronavirus_HKU4_Strain_NameSZ140324_Segmentnull_HostUnknown.fa | B333 |
| EF065508_OrganismBat_coronavirus_HKU4_4_Strain_NameLMH1f_Segmentnull_HostBat.fa | B334 |
| EF065506_OrganismBat_coronavirus_HKU4_2_Strain_NameB05f_Segmentnull_HostBat.fa | B335 |
| NC_009019_OrganismTylonycteris_bat_coronavirus_HKU4_Strain_NameHKU4_1_B04f_Segmentnull_HostBat.fa | B336 |
| EF065505_OrganismBat_coronavirus_HKU4_1_Strain_NameB04f_Segmentnull_HostBat.fa | B337 |
| EF065507_OrganismBat_coronavirus_HKU4_3_Strain_NameB07f_Segmentnull_HostBat.fa | B338 |
| MH002337_OrganismTylonycteris_bat_coronavirus_HKU4_Strain_NameCZ01_Segmentnull_HostUnknown.fa | B339 |
| MH002338_OrganismTylonycteris_bat_coronavirus_HKU4_Strain_NameCZ07_Segmentnull_HostUnknown.fa | B340 |
| KJ473822_OrganismBtTp_BetaCoV_GX2012_Strain_NameBtTp_GX2012_Segmentnull_HostBat.fa | B341 |
| MK907287_OrganismErinaceus_hedgehog_coronavirus_HKU31_Strain_NameRs13_Segmentnull_HostUnknown.fa | B342 |
| MK907286_OrganismErinaceus_hedgehog_coronavirus_HKU31_Strain_NameF6_Segmentnull_HostUnknown.fa | B343 |
| MK679660_OrganismHedgehog_coronavirus_1_Strain_NameUNKNOWN_MK679660_Segmentnull_HostHedgehog.fa | B344 |
| KC545386_OrganismBetacoronavirus_Erinaceus_VMC_DEU_2012_Strain_NameErinaceusCoV_2012_216_GER_2012_Segmentnull_HostHedgehog.fa | B345 |
| NC_022643_OrganismBetacoronavirus_Erinaceus_VMC_DEU_2012_Strain_NameErinaceusCoV_2012_216_GER_2012_Segmentnull_HostHedgehog.fa | B346 |
| NC_039207_OrganismBetacoronavirus_Erinaceus_VMC_DEU_2012_Strain_NameErinaceusCoV_2012_174_GER_2012_Segmentnull_HostHedgehog.fa | B347 |
| KC545383_OrganismBetacoronavirus_Erinaceus_VMC_DEU_2012_Strain_NameErinaceusCoV_2012_174_GER_2012_Segmentnull_HostHedgehog.fa | B348 |
| KF923898_OrganismHuman_coronavirus_OC43_Strain_Name3184A_2012_Segmentnull_HostHuman.fa | B349 |
| MG197719_OrganismHuman_coronavirus_OC43_Strain_NameYC_67_Segmentnull_HostUnknown.fa | B350 |
| MG197714_OrganismHuman_coronavirus_OC43_Strain_NameCC_23_Segmentnull_HostUnknown.fa | B351 |
| MN306043_OrganismHuman_coronavirus_OC43_Strain_NameHCoV_OC43_Seattle_USA_SC0841_2019_Segmentnull_HostHuman.fa | B352 |
| MN310476_OrganismHuman_coronavirus_OC43_Strain_NameHCoV_OC43_Seattle_USA_SC9428_2018_Segmentnull_HostHuman.fa | B353 |
| MK303625_OrganismHuman_coronavirus_OC43_Strain_NameMDS16_Segmentnull_HostUnknown.fa | B354 |
| MG197709_OrganismHuman_coronavirus_OC43_Strain_NameBJ_112_Segmentnull_HostUnknown.fa | B355 |
| MG197711_OrganismHuman_coronavirus_OC43_Strain_NameBJ_164_Segmentnull_HostUnknown.fa | B356 |
| MG197720_OrganismHuman_coronavirus_OC43_Strain_NameYC_68_Segmentnull_HostUnknown.fa | B357 |
| MG197718_OrganismHuman_coronavirus_OC43_Strain_NameYC_55_Segmentnull_HostUnknown.fa | B358 |
| MG197721_OrganismHuman_coronavirus_OC43_Strain_NameYC_72_Segmentnull_HostUnknown.fa | B359 |
| KF923886_OrganismHuman_coronavirus_OC43_Strain_Name1908A_2010_Segmentnull_HostHuman.fa | B360 |
| AY903459_OrganismHuman_coronavirus_OC43_Strain_Name87309_Belgium_2003_Segmentnull_HostUnknown.fa | B361 |
| KF923889_OrganismHuman_coronavirus_OC43_Strain_Name1926_2006_Segmentnull_HostHuman.fa | B362 |
| KF923888_OrganismHuman_coronavirus_OC43_Strain_Name2145A_2010_Segmentnull_HostHuman.fa | B363 |
| KF923887_OrganismHuman_coronavirus_OC43_Strain_Name1997A_2010_Segmentnull_HostHuman.fa | B364 |
| AY903460_OrganismHuman_coronavirus_OC43_Strain_Name19572_Belgium_2004_Segmentnull_HostUnknown.fa | B365 |
| JN129835_OrganismHuman_coronavirus_OC43_Strain_NameHK04_02_Segmentnull_SubtypeD_HostHuman.fa | B366 |
| KY674918_OrganismHuman_coronavirus_OC43_Strain_NameN07_1647B_Segmentnull_HostHuman.fa | B367 |
| KY674917_OrganismHuman_coronavirus_OC43_Strain_NameN07_1609B_Segmentnull_HostHuman.fa | B368 |
| KY554972_OrganismHuman_coronavirus_OC43_Strain_NameN07_1541B_433X_Segmentnull_HostHuman.fa | B369 |
| KY554973_OrganismHuman_coronavirus_OC43_Strain_NameN07_1689B_116X_Segmentnull_HostHuman.fa | B370 |
| KF923891_OrganismHuman_coronavirus_OC43_Strain_Name5240_2007_Segmentnull_HostHuman.fa | B371 |
| KF923894_OrganismHuman_coronavirus_OC43_Strain_Name5352_2007_Segmentnull_HostHuman.fa | B372 |
| KF923921_OrganismHuman_coronavirus_OC43_Strain_Name69A_2007_Segmentnull_HostHuman.fa | B373 |
| KF923923_OrganismHuman_coronavirus_OC43_Strain_Name892A_2008_Segmentnull_HostHuman.fa | B374 |
| KF923910_OrganismHuman_coronavirus_OC43_Strain_Name5445_2007_Segmentnull_HostHuman.fa | B375 |
| KF923925_OrganismHuman_coronavirus_OC43_Strain_Name10574_2010_Segmentnull_HostHuman.fa | B376 |
| KJ958218_OrganismHuman_coronavirus_OC43_Strain_NameLY341_Segmentnull_HostHuman.fa | B377 |
| KJ958219_OrganismHuman_coronavirus_OC43_Strain_NameLY342_Segmentnull_HostHuman.fa | B378 |
| KF923922_OrganismHuman_coronavirus_OC43_Strain_Name8164_2009_Segmentnull_HostHuman.fa | B379 |
| KF923918_OrganismHuman_coronavirus_OC43_Strain_Name10108_2010_Segmentnull_HostHuman.fa | B380 |
| KF923893_OrganismHuman_coronavirus_OC43_Strain_Name2151A_2010_Segmentnull_HostHuman.fa | B381 |
| KF923895_OrganismHuman_coronavirus_OC43_Strain_Name10285_2010_Segmentnull_HostHuman.fa | B382 |
| KF923924_OrganismHuman_coronavirus_OC43_Strain_Name10290_2010_Segmentnull_HostHuman.fa | B383 |
| KF923919_OrganismHuman_coronavirus_OC43_Strain_Name5595_2007_Segmentnull_HostHuman.fa | B384 |
| KF923908_OrganismHuman_coronavirus_OC43_Strain_Name5414_2007_Segmentnull_HostHuman.fa | B385 |
| KF923892_OrganismHuman_coronavirus_OC43_Strain_Name5345_2007_Segmentnull_HostHuman.fa | B386 |
| KF923909_OrganismHuman_coronavirus_OC43_Strain_Name5442_2007_Segmentnull_HostHuman.fa | B387 |
| KF923916_OrganismHuman_coronavirus_OC43_Strain_Name5519_2007_Segmentnull_HostHuman.fa | B388 |
| KF923907_OrganismHuman_coronavirus_OC43_Strain_Name5370_2007_Segmentnull_HostHuman.fa | B389 |
| KF923912_OrganismHuman_coronavirus_OC43_Strain_Name5484_2007_Segmentnull_HostHuman.fa | B390 |
| KF923901_OrganismHuman_coronavirus_OC43_Strain_Name5472_2007_Segmentnull_HostHuman.fa | B391 |
| KF923917_OrganismHuman_coronavirus_OC43_Strain_Name5566_2007_Segmentnull_HostHuman.fa | B392 |
| KF923920_OrganismHuman_coronavirus_OC43_Strain_Name5617_2007_Segmentnull_HostHuman.fa | B393 |
| KF923913_OrganismHuman_coronavirus_OC43_Strain_Name5485_2007_Segmentnull_HostHuman.fa | B394 |
| KF923915_OrganismHuman_coronavirus_OC43_Strain_Name5517_2007_Segmentnull_HostHuman.fa | B395 |
| KF923914_OrganismHuman_coronavirus_OC43_Strain_Name5508_2007_Segmentnull_HostHuman.fa | B396 |
| KF923911_OrganismHuman_coronavirus_OC43_Strain_Name5479_2007_Segmentnull_HostHuman.fa | B397 |
| KY554974_OrganismHuman_coronavirus_OC43_Strain_NameN08_33B_360X_Segmentnull_HostHuman.fa | B398 |
| KY674920_OrganismHuman_coronavirus_OC43_Strain_NameN09_595B_Segmentnull_HostHuman.fa | B399 |
| KY554975_OrganismHuman_coronavirus_OC43_Strain_NameN09_382B_Segmentnull_HostHuman.fa | B400 |
| KF923890_OrganismHuman_coronavirus_OC43_Strain_Name39A_2007_Segmentnull_HostHuman.fa | B401 |
| KX344031_OrganismHuman_coronavirus_OC43_Strain_NameOC43_human_Mex_LRTI_238_2011_Segmentnull_HostHuman.fa | B402 |
| KX538968_OrganismHuman_coronavirus_OC43_Strain_NameMY_U464_12_Segmentnull_HostHuman.fa | B403 |
| KX538971_OrganismHuman_coronavirus_OC43_Strain_NameMY_U732_12_Segmentnull_HostHuman.fa | B404 |
| KX538974_OrganismHuman_coronavirus_OC43_Strain_NameMY_U945_12_Segmentnull_HostHuman.fa | B405 |
| KX538967_OrganismHuman_coronavirus_OC43_Strain_NameMY_U413_12_Segmentnull_HostHuman.fa | B406 |
| KX538965_OrganismHuman_coronavirus_OC43_Strain_NameMY_U208_12_Segmentnull_HostHuman.fa | B407 |
| KX538973_OrganismHuman_coronavirus_OC43_Strain_NameMY_U868_12_Segmentnull_HostHuman.fa | B408 |
| KF923903_OrganismHuman_coronavirus_OC43_Strain_Name12691_2012_Segmentnull_HostHuman.fa | B409 |
| KX538977_OrganismHuman_coronavirus_OC43_Strain_NameMY_U1140_12_Segmentnull_HostHuman.fa | B410 |
| KX538969_OrganismHuman_coronavirus_OC43_Strain_NameMY_U523_12_Segmentnull_HostHuman.fa | B411 |
| KX538975_OrganismHuman_coronavirus_OC43_Strain_NameMY_U1024_12_Segmentnull_HostHuman.fa | B412 |
| KF923897_OrganismHuman_coronavirus_OC43_Strain_Name3269A_2012_Segmentnull_HostHuman.fa | B413 |
| KX538970_OrganismHuman_coronavirus_OC43_Strain_NameMY_U710_12_Segmentnull_HostHuman.fa | B414 |
| MN026164_OrganismHuman_coronavirus_OC43_Strain_NameOC43_KLF_01_2018_Segmentnull_HostHuman.fa | B415 |
| MG977449_OrganismHuman_coronavirus_OC43_Strain_NameTNP_F1834_2_Segmentnull_HostChimpanzee.fa | B416 |
| MG977447_OrganismHuman_coronavirus_OC43_Strain_NameTNP_F1832_2_Segmentnull_HostChimpanzee.fa | B417 |
| MG977444_OrganismHuman_coronavirus_OC43_Strain_NameTNP_F1778_2_Segmentnull_HostChimpanzee.fa | B418 |
| MG977445_OrganismHuman_coronavirus_OC43_Strain_NameTNP_F1790_2_Segmentnull_HostChimpanzee.fa | B419 |
| MG977452_OrganismHuman_coronavirus_OC43_Strain_NameTNP_12643_Segmentnull_HostHuman.fa | B420 |
| MG977451_OrganismHuman_coronavirus_OC43_Strain_NameTNP_12636_Segmentnull_HostHuman.fa | B421 |
| MK303620_OrganismHuman_coronavirus_OC43_Strain_NameMDS2_Segmentnull_HostUnknown.fa | B422 |
| MG197713_OrganismHuman_coronavirus_OC43_Strain_NameBJ_221_Segmentnull_HostUnknown.fa | B423 |
| MG197722_OrganismHuman_coronavirus_OC43_Strain_NameYC_207_Segmentnull_HostUnknown.fa | B424 |
| MG197717_OrganismHuman_coronavirus_OC43_Strain_NameWZ_522_Segmentnull_HostUnknown.fa | B425 |
| MG197716_OrganismHuman_coronavirus_OC43_Strain_NameWZ_303_Segmentnull_HostUnknown.fa | B426 |
| MG197715_OrganismHuman_coronavirus_OC43_Strain_NameGZYF_26_Segmentnull_HostUnknown.fa | B427 |
| MG197710_OrganismHuman_coronavirus_OC43_Strain_NameBJ_124_Segmentnull_HostUnknown.fa | B428 |
| MG197712_OrganismHuman_coronavirus_OC43_Strain_NameBJ_165_Segmentnull_HostUnknown.fa | B429 |
| KF923902_OrganismHuman_coronavirus_OC43_Strain_Name12689_2012_Segmentnull_HostHuman.fa | B430 |
| KY967356_OrganismHuman_coronavirus_OC43_Strain_NameHCoV_OC43_Seattle_USA_SC2924_2015_Segmentnull_HostHuman.fa | B431 |
| KX538978_OrganismHuman_coronavirus_OC43_Strain_NameMY_U1758_13_Segmentnull_HostHuman.fa | B432 |
| KX538966_OrganismHuman_coronavirus_OC43_Strain_NameMY_U236_12_Segmentnull_HostHuman.fa | B433 |
| KX538979_OrganismHuman_coronavirus_OC43_Strain_NameMY_U1975_13_Segmentnull_HostHuman.fa | B434 |
| MK303622_OrganismHuman_coronavirus_OC43_Strain_NameMDS11_Segmentnull_HostUnknown.fa | B435 |
| KX538972_OrganismHuman_coronavirus_OC43_Strain_NameMY_U774_12_Segmentnull_HostHuman.fa | B436 |
| KF923904_OrganismHuman_coronavirus_OC43_Strain_Name12694_2012_Segmentnull_HostHuman.fa | B437 |
| KX538964_OrganismHuman_coronavirus_OC43_Strain_NameMY_U002_12_Segmentnull_HostHuman.fa | B438 |
| KX538976_OrganismHuman_coronavirus_OC43_Strain_NameMY_U1057_12_Segmentnull_HostHuman.fa | B439 |
| MF374983_OrganismHuman_coronavirus_OC43_Strain_NameHCoV_OC43_USA_TCNP_0070_2016_Segmentnull_HostHuman.fa | B440 |
| MN306042_OrganismHuman_coronavirus_OC43_Strain_NameHCoV_OC43_Seattle_USA_SC0839_2019_Segmentnull_HostHuman.fa | B441 |
| MN306053_OrganismHuman_coronavirus_OC43_Strain_NameHCoV_OC43_Seattle_USA_SC9430_2018_Segmentnull_HostHuman.fa | B442 |
| MN306036_OrganismHuman_coronavirus_OC43_Strain_NameHCoV_OC43_Seattle_USA_SC0682_2019_Segmentnull_HostHuman.fa | B443 |
| MN306041_OrganismHuman_coronavirus_OC43_Strain_NameHCoV_OC43_Seattle_USA_SC0810_2019_Segmentnull_HostHuman.fa | B444 |
| MN310478_OrganismHuman_coronavirus_OC43_Strain_NameHCoV_OC43_Seattle_USA_SC0776_2019_Segmentnull_HostHuman.fa | B445 |
| KY983585_OrganismHuman_coronavirus_OC43_Strain_NameHCoV_OC43_Seattle_USA_SC2854_2015_Segmentnull_HostHuman.fa | B446 |
| KY967361_OrganismHuman_coronavirus_OC43_Strain_NameHCoV_OC43_Seattle_USA_SC2345_2015_Segmentnull_HostHuman.fa | B447 |
| KY967359_OrganismHuman_coronavirus_OC43_Strain_NameHCoV_OC43_Seattle_USA_SC2730_2015_Segmentnull_HostHuman.fa | B448 |
| MG197723_OrganismHuman_coronavirus_OC43_Strain_NameHZ_459_Segmentnull_HostUnknown.fa | B449 |
| KY369906_OrganismHuman_coronavirus_OC43_Strain_NameHCoV_OC43_Seattle_USA_SC622_2016_Segmentnull_HostHuman.fa | B450 |
| KY369905_OrganismHuman_coronavirus_OC43_Strain_NameHCoV_OC43_Seattle_USA_SC831_2016_Segmentnull_HostHuman.fa | B451 |
| KY983583_OrganismHuman_coronavirus_OC43_Strain_NameHCoV_OC43_Seattle_USA_SC2481_2015_Segmentnull_HostHuman.fa | B452 |
| KY684759_OrganismHuman_coronavirus_OC43_Strain_NameHCoV_OC43_Seattle_USA_SC2269_2016_Segmentnull_HostHuman.fa | B453 |
| KY967358_OrganismHuman_coronavirus_OC43_Strain_NameHCoV_OC43_Seattle_USA_SC2770_2015_Segmentnull_HostHuman.fa | B454 |
| MF374984_OrganismHuman_coronavirus_OC43_Strain_NameHCoV_OC43_USA_TCNP_00204_2017_Segmentnull_HostHuman.fa | B455 |
| KY983588_OrganismHuman_coronavirus_OC43_Strain_NameHCoV_OC43_Seattle_USA_SC3118_2015_Segmentnull_HostHuman.fa | B456 |
| MF374985_OrganismHuman_coronavirus_OC43_Strain_NameHCoV_OC43_USA_TCNP_00212_2017_Segmentnull_HostHuman.fa | B457 |
| MH121121_OrganismHuman_coronavirus_OC43_Strain_NameHCoV_OC43_USA_ACRI_0213_2016_Segmentnull_HostHuman.fa | B458 |
| KY369907_OrganismHuman_coronavirus_OC43_Strain_NameHCoV_OC43_Seattle_USA_SC9741_2016_Segmentnull_HostHuman.fa | B459 |
| MK303621_OrganismHuman_coronavirus_OC43_Strain_NameMDS4_Segmentnull_HostUnknown.fa | B460 |
| MK303623_OrganismHuman_coronavirus_OC43_Strain_NameMDS12_Segmentnull_HostUnknown.fa | B461 |
| MK303619_OrganismHuman_coronavirus_OC43_Strain_NameMDS6_Segmentnull_HostUnknown.fa | B462 |
| MK303624_OrganismHuman_coronavirus_OC43_Strain_NameMDS14_Segmentnull_HostUnknown.fa | B463 |
| JN129834_OrganismHuman_coronavirus_OC43_Strain_NameHK04_01_Segmentnull_SubtypeC_HostHuman.fa | B464 |
| KF923905_OrganismHuman_coronavirus_OC43_Strain_Name229_2005_Segmentnull_HostHuman.fa | B465 |
| KF923900_OrganismHuman_coronavirus_OC43_Strain_Name3647_2006_Segmentnull_HostHuman.fa | B466 |
| KF923899_OrganismHuman_coronavirus_OC43_Strain_Name3582_2006_Segmentnull_HostHuman.fa | B467 |
| KF530070_OrganismHuman_coronavirus_OC43_Strain_NameOC43_human_USA_991_19_1999_Segmentnull_HostHuman.fa | B468 |
| KF530081_OrganismHuman_coronavirus_OC43_Strain_NameOC43_human_USA_991_5_1999_Segmentnull_HostHuman.fa | B469 |
| KF530068_OrganismHuman_coronavirus_OC43_Strain_NameOC43_human_USA_007_11_2000_Segmentnull_HostHuman.fa | B470 |
| AY391777_OrganismHuman_coronavirus_OC43_Strain_NameATCC_VR_759_Segmentnull_HostUnknown.fa | B471 |
| NC_006213_OrganismHuman_coronavirus_OC43_Strain_NameATCC_VR_759_Segmentnull_SubtypeOC43_HostUnknown.fa | B472 |
| AY585228_OrganismHuman_coronavirus_OC43_Strain_NameATCC_VR_759_Segmentnull_SubtypeOC43_HostUnknown.fa | B473 |
| AY585229_OrganismHuman_coronavirus_OC43_Strain_NameUNKNOWN_AY585229_Segmentnull_SubtypeOC43_Paris_HostUnknown.fa | B474 |
| KF530085_OrganismHuman_coronavirus_OC43_Strain_NameOC43_human_USA_871_25_1987_Segmentnull_HostHuman.fa | B475 |
| KF530066_OrganismHuman_coronavirus_OC43_Strain_NameOC43_human_USA_901_33_1990_Segmentnull_HostHuman.fa | B476 |
| KF530061_OrganismHuman_coronavirus_OC43_Strain_NameOC43_human_USA_901_43_1990_Segmentnull_HostHuman.fa | B477 |
| KF530073_OrganismHuman_coronavirus_OC43_Strain_NameOC43_human_USA_8912_37_1989_Segmentnull_HostHuman.fa | B478 |
| KF530065_OrganismHuman_coronavirus_OC43_Strain_NameOC43_human_USA_901_41_1990_Segmentnull_HostHuman.fa | B479 |
| KF530075_OrganismHuman_coronavirus_OC43_Strain_NameOC43_human_USA_953_23_1995_Segmentnull_HostHuman.fa | B480 |
| KF530090_OrganismHuman_coronavirus_OC43_Strain_NameOC43_human_USA_931_85_1993_Segmentnull_HostHuman.fa | B481 |
| KF530097_OrganismHuman_coronavirus_OC43_Strain_NameOC43_human_USA_9211_43_1992_Segmentnull_HostHuman.fa | B482 |
| KF530074_OrganismHuman_coronavirus_OC43_Strain_NameOC43_human_USA_9212_33_1992_Segmentnull_HostHuman.fa | B483 |
| KF530087_OrganismHuman_coronavirus_OC43_Strain_NameOC43_human_USA_873_6_1987_Segmentnull_HostHuman.fa | B484 |
| KF530060_OrganismHuman_coronavirus_OC43_Strain_NameOC43_human_USA_851_15_1985_Segmentnull_HostHuman.fa | B485 |
| KF530086_OrganismHuman_coronavirus_OC43_Strain_NameOC43_human_USA_872_5_1987_Segmentnull_HostHuman.fa | B486 |
| KF530077_OrganismHuman_coronavirus_OC43_Strain_NameOC43_human_USA_873_16_1987_Segmentnull_HostHuman.fa | B487 |
| KF530083_OrganismHuman_coronavirus_OC43_Strain_NameOC43_human_USA_873_19_1987_Segmentnull_HostHuman.fa | B488 |
| DQ011855_OrganismPorcine_hemagglutinating_encephalomyelitis_virus_Strain_NameVW572_Segmentnull_HostUnknown.fa | B489 |
| KY994645_OrganismPorcine_hemagglutinating_encephalomyelitis_virus_Strain_NameJL_2008_Segmentnull_HostSwine.fa | B490 |
| MF083115_OrganismPorcine_hemagglutinating_encephalomyelitis_virus_Strain_NameCC14_Segmentnull_HostSwine.fa | B491 |
| KY419106_OrganismPorcine_hemagglutinating_encephalomyelitis_virus_Strain_NamePHEV_CoV_USA_15TOSU1785_Segmentnull_HostSwine.fa | B492 |
| KY419113_OrganismPorcine_hemagglutinating_encephalomyelitis_virus_Strain_NamePHEV_CoV_USA_15TOSU1582_Segmentnull_HostSwine.fa | B493 |
| KY419107_OrganismPorcine_hemagglutinating_encephalomyelitis_virus_Strain_NamePHEV_CoV_USA_15TOSU1209_Segmentnull_HostSwine.fa | B494 |
| KY419111_OrganismPorcine_hemagglutinating_encephalomyelitis_virus_Strain_NamePHEV_CoV_USA_15TOSU1727_Segmentnull_HostSwine.fa | B495 |
| KY419109_OrganismPorcine_hemagglutinating_encephalomyelitis_virus_Strain_NamePHEV_CoV_USA_15TOSU1655_Segmentnull_HostSwine.fa | B496 |
| KY419103_OrganismPorcine_hemagglutinating_encephalomyelitis_virus_Strain_NamePHEV_CoV_USA_15TOSU25049_Segmentnull_HostSwine.fa | B497 |
| KY419104_OrganismPorcine_hemagglutinating_encephalomyelitis_virus_Strain_NamePHEV_CoV_USA_15TOSU0331_Segmentnull_HostSwine.fa | B498 |
| KY419112_OrganismPorcine_hemagglutinating_encephalomyelitis_virus_Strain_NamePHEV_CoV_USA_15TOSU1765_Segmentnull_HostSwine.fa | B499 |
| KY419108_OrganismPorcine_hemagglutinating_encephalomyelitis_virus_Strain_NamePHEV_CoV_USA_15TOSU24992_Segmentnull_HostSwine.fa | B500 |
| KY419110_OrganismPorcine_hemagglutinating_encephalomyelitis_virus_Strain_NamePHEV_CoV_USA_15TOSU1362_Segmentnull_HostSwine.fa | B501 |
| KY419105_OrganismPorcine_hemagglutinating_encephalomyelitis_virus_Strain_NamePHEV_CoV_USA_15TOSU0582_Segmentnull_HostSwine.fa | B502 |
| LC061274_OrganismEquine_coronavirus_Strain_NameObihiro12_2_Segmentnull_HostHorse.fa | B503 |
| LC061273_OrganismEquine_coronavirus_Strain_NameObihiro12_1_Segmentnull_HostHorse.fa | B504 |
| EF446615_OrganismEquine_coronavirus_Strain_NameNC99_Segmentnull_HostUnknown.fa | B505 |
| LC061272_OrganismEquine_coronavirus_Strain_NameTokachi09_Segmentnull_HostHorse.fa | B506 |
| JN874561_OrganismRabbit_coronavirus_HKU14_Strain_NameHKU14_8_Segmentnull_HostRabbit.fa | B507 |
| JN874560_OrganismRabbit_coronavirus_HKU14_Strain_NameHKU14_3_Segmentnull_HostRabbit.fa | B508 |
| JN874559_OrganismRabbit_coronavirus_HKU14_Strain_NameHKU14_1_Segmentnull_HostRabbit.fa | B509 |
| NC_017083_OrganismRabbit_coronavirus_HKU14_Strain_NameHKU14_1_Segmentnull_HostRabbit.fa | B510 |
| JN874562_OrganismRabbit_coronavirus_HKU14_Strain_NameHKU14_10_Segmentnull_HostRabbit.fa | B511 |
| FJ647223_OrganismMurine_coronavirus_MHV_1_Strain_NameMHV_1_Segmentnull_HostMouse.fa | B512 |
| AB551247_OrganismMurine_hepatitis_virus_Strain_NameMHV_MI_Segmentnull_HostMouse.fa | B513 |
| FJ647224_OrganismMurine_coronavirus_MHV_3_Strain_NameMHV_3_Segmentnull_HostMouse.fa | B514 |
| FJ647227_OrganismMurine_coronavirus_repJHM_RA59_Strain_NamerepJHM_RA59_Segmentnull_HostMouse.fa | B515 |
| FJ647222_OrganismMurine_coronavirus_SA59_RJHM_Strain_NameSA59_RJHM_Segmentnull_HostMouse.fa | B516 |
| FJ647219_OrganismMurine_coronavirus_RJHM_A_Strain_NameRJHM_A_Segmentnull_HostMouse.fa | B517 |
| FJ647226_OrganismMurine_coronavirus_MHV_JHM.IA_Strain_NameMHV_JHM.IA_Segmentnull_HostMouse.fa | B518 |
| AC_000192_OrganismMurine_hepatitis_virus_strain_JHM_Strain_NameUNKNOWN_AC_000192_Segmentnull_HostUnknown.fa | B519 |
| JX169867_OrganismMurine_coronavirus_Strain_NameJHM.WU_Segmentnull_HostUnknown.fa | B520 |
| JX169866_OrganismMurine_coronavirus_Strain_NameJHM_WU_Dns2_Segmentnull_HostUnknown.fa | B521 |
| NC_001846_OrganismMurine_hepatitis_virus_Strain_NameMHV_A59_Segmentnull_HostUnknown.fa | B522 |
| AF029248_OrganismMurine_hepatitis_virus_Strain_NameMHV_A59_Segmentnull_HostUnknown.fa | B523 |
| FJ884687_OrganismMurine_hepatitis_virus_strain_A59_Strain_NameA59_Segmentnull_HostMouse.fa | B524 |
| FJ884686_OrganismMurine_hepatitis_virus_strain_A59_Strain_NameA59_Segmentnull_HostMouse.fa | B525 |
| FJ647220_OrganismMurine_coronavirus_RA59_SJHM_Strain_NameRA59_SJHM_Segmentnull_HostMouse.fa | B526 |
| FJ647221_OrganismMurine_coronavirus_repA59_RJHM_Strain_NamerepA59_RJHM_Segmentnull_HostMouse.fa | B527 |
| FJ647218_OrganismMurine_coronavirus_RA59_R13_Strain_NameRA59_R13_Segmentnull_HostMouse.fa | B528 |
| KF268338_OrganismMurine_coronavirus_Strain_NameMHV_BHKR_lab_USA_icA59_L94P_2012_Segmentnull_HostMouse.fa | B529 |
| MF618252_OrganismMurine_hepatitis_virus_Strain_NameA59_Segmentnull_HostMouse.fa | B530 |
| MF618253_OrganismMurine_hepatitis_virus_Strain_NameA59_Segmentnull_HostMouse.fa | B531 |
| KP887098_OrganismMurine_coronavirus_Strain_NameAM2_Segmentnull_HostUnknown.fa | B532 |
| KF268339_OrganismMurine_coronavirus_Strain_NameMHV_BHKR_lab_USA_icA59_ns2M_2012_Segmentnull_HostMouse.fa | B533 |
| KF268337_OrganismMurine_coronavirus_Strain_NameMHV_BHKR_lab_USA_icA59_17Cl1_2012_Segmentnull_HostMouse.fa | B534 |
| AF208067_OrganismMurine_hepatitis_virus_Strain_NameML_10_Segmentnull_HostUnknown.fa | B535 |
| AY700211_OrganismMurine_hepatitis_virus_Strain_NameA59_Segmentnull_HostUnknown.fa | B536 |
| KF268336_OrganismMurine_coronavirus_Strain_NameMHV_BHKR_lab_USA_infA59_H126A_2012_Segmentnull_HostMouse.fa | B537 |
| FJ647225_OrganismMurine_coronavirus_inf_MHV_A59_Strain_Nameinf_MHV_A59_Segmentnull_HostMouse.fa | B538 |
| GU593319_OrganismMurine_hepatitis_virus_Strain_NameS_Segmentnull_HostMouse.fa | B539 |
| JQ173883_OrganismMurine_hepatitis_virus_strain_S_3239_17_Strain_NameMHV_S_3239_17_Segmentnull_HostUnknown.fa | B540 |
| JF792616_OrganismRat_coronavirus_Strain_Name681_Segmentnull_HostRat.fa | B541 |
| JF792617_OrganismRat_coronavirus_Strain_Name8190_Segmentnull_HostRat.fa | B542 |
| FJ938068_OrganismRat_coronavirus_Parker_Strain_NameParker_Segmentnull_HostRat.fa | B543 |
| NC_012936_OrganismRat_coronavirus_Parker_Strain_NameParker_Segmentnull_HostRat.fa | B544 |
| AF208066_OrganismMurine_hepatitis_virus_Strain_NamePenn_97_1_Segmentnull_HostUnknown.fa | B545 |
| AF207902_OrganismMurine_hepatitis_virus_strain_ML_11_Strain_NameML_11_Segmentnull_HostUnknown.fa | B546 |
| AF201929_OrganismMurine_hepatitis_virus_strain_2_Strain_NameMHV_2_Segmentnull_HostUnknown.fa | B547 |
| DQ415902_OrganismHuman_coronavirus_HKU1_Strain_NameN25_Segmentnull_HostUnknown.fa | B548 |
| DQ415911_OrganismHuman_coronavirus_HKU1_Strain_NameN15_Segmentnull_HostUnknown.fa | B549 |
| MK167038_OrganismHuman_coronavirus_HKU1_Strain_NameSC2521_Segmentnull_HostHuman.fa | B550 |
| KF686338_OrganismHuman_coronavirus_HKU1_Strain_NameHKU1_human_USA_HKU1_1_2005_Segmentnull_HostHuman.fa | B551 |
| KY674921_OrganismHuman_coronavirus_HKU1_Strain_NameN08_87_Segmentnull_HostHuman.fa | B552 |
| MH940245_OrganismHuman_coronavirus_HKU1_Strain_NameSI17244_Segmentnull_HostHuman.fa | B553 |
| AY884001_OrganismHuman_coronavirus_HKU1_Strain_NameUNKNOWN_AY884001_Segmentnull_HostUnknown.fa | B554 |
| DQ415898_OrganismHuman_coronavirus_HKU1_Strain_NameN21_Segmentnull_HostUnknown.fa | B555 |
| DQ415899_OrganismHuman_coronavirus_HKU1_Strain_NameN22_Segmentnull_HostUnknown.fa | B556 |
| DQ415913_OrganismHuman_coronavirus_HKU1_Strain_NameN17_Segmentnull_HostUnknown.fa | B557 |
| DQ415912_OrganismHuman_coronavirus_HKU1_Strain_NameN16_Segmentnull_HostUnknown.fa | B558 |
| DQ415897_OrganismHuman_coronavirus_HKU1_Strain_NameN20_Segmentnull_HostUnknown.fa | B559 |
| DQ339101_OrganismHuman_coronavirus_HKU1_Strain_NameN5P8_Segmentnull_HostUnknown.fa | B560 |
| KF686343_OrganismHuman_coronavirus_HKU1_Strain_NameHKU1_human_USA_HKU1_13_2010_Segmentnull_HostHuman.fa | B561 |
| KF686342_OrganismHuman_coronavirus_HKU1_Strain_NameHKU1_human_USA_HKU1_11_2009_Segmentnull_HostHuman.fa | B562 |
| KF686346_OrganismHuman_coronavirus_HKU1_Strain_NameHKU1_human_USA_HKU1_12_2010_Segmentnull_HostHuman.fa | B563 |
| KY674941_OrganismHuman_coronavirus_HKU1_Strain_NameN09_1663B_Segmentnull_HostHuman.fa | B564 |
| KY674943_OrganismHuman_coronavirus_HKU1_Strain_NameN09_1605B_Segmentnull_HostHuman.fa | B565 |
| KY674942_OrganismHuman_coronavirus_HKU1_Strain_NameN09_1627B_Segmentnull_HostHuman.fa | B566 |
| KF686339_OrganismHuman_coronavirus_HKU1_Strain_NameHKU1_human_USA_HKU1_3_2009_Segmentnull_HostHuman.fa | B567 |
| KF686340_OrganismHuman_coronavirus_HKU1_Strain_NameHKU1_human_USA_HKU1_5_2009_Segmentnull_HostHuman.fa | B568 |
| KF686345_OrganismHuman_coronavirus_HKU1_Strain_NameHKU1_human_USA_HKU1_20_2010_Segmentnull_HostHuman.fa | B569 |
| KF430200_OrganismHuman_coronavirus_HKU1_Strain_NameHKU1_human_USA_HKU1_16_2010_Segmentnull_HostHuman.fa | B570 |
| KF430201_OrganismHuman_coronavirus_HKU1_Strain_NameHKU1_human_USA_HKU1_18_2010_Segmentnull_HostHuman.fa | B571 |
| KF686341_OrganismHuman_coronavirus_HKU1_Strain_NameHKU1_human_USA_HKU1_10_2010_Segmentnull_HostHuman.fa | B572 |
| KF430199_OrganismHuman_coronavirus_HKU1_Strain_NameHKU1_human_USA_HKU1_14_2009_Segmentnull_HostHuman.fa | B573 |
| KF430202_OrganismHuman_coronavirus_HKU1_Strain_NameHKU1_human_USA_HKU1_7_2010_Segmentnull_HostHuman.fa | B574 |
| KF430196_OrganismHuman_coronavirus_HKU1_Strain_NameHKU1_human_USA_HKU1_4_2005_Segmentnull_HostHuman.fa | B575 |
| KF850450_OrganismHuman_coronavirus_HKU1_Strain_NameHKU1_human_USA_1102_2005_Segmentnull_HostHuman.fa | B576 |
| HM034837_OrganismHuman_coronavirus_HKU1_Strain_NameCaen1_Segmentnull_HostHuman.fa | B577 |
| DQ415896_OrganismHuman_coronavirus_HKU1_Strain_NameN19_Segmentnull_HostUnknown.fa | B578 |
| DQ415909_OrganismHuman_coronavirus_HKU1_Strain_NameN13_Segmentnull_HostUnknown.fa | B579 |
| KF686344_OrganismHuman_coronavirus_HKU1_Strain_NameHKU1_human_USA_HKU1_15_2009_Segmentnull_HostHuman.fa | B580 |
| KT779555_OrganismHuman_coronavirus_HKU1_Strain_NameBJ01_p3_Segmentnull_HostHuman.fa | B581 |
| KT779556_OrganismHuman_coronavirus_HKU1_Strain_NameBJ01_p9_Segmentnull_HostHuman.fa | B582 |
| DQ415901_OrganismHuman_coronavirus_HKU1_Strain_NameN24_Segmentnull_HostUnknown.fa | B583 |
| DQ415900_OrganismHuman_coronavirus_HKU1_Strain_NameN23_Segmentnull_HostUnknown.fa | B584 |
| DQ415906_OrganismHuman_coronavirus_HKU1_Strain_NameN9_Segmentnull_HostUnknown.fa | B585 |
| DQ415908_OrganismHuman_coronavirus_HKU1_Strain_NameN11_Segmentnull_HostUnknown.fa | B586 |
| DQ415910_OrganismHuman_coronavirus_HKU1_Strain_NameN14_Segmentnull_HostUnknown.fa | B587 |
| DQ415905_OrganismHuman_coronavirus_HKU1_Strain_NameN7_Segmentnull_HostUnknown.fa | B588 |
| DQ415904_OrganismHuman_coronavirus_HKU1_Strain_NameN6_Segmentnull_HostUnknown.fa | B589 |
| DQ415907_OrganismHuman_coronavirus_HKU1_Strain_NameN10_Segmentnull_HostUnknown.fa | B590 |
| DQ415914_OrganismHuman_coronavirus_HKU1_Strain_NameN18_Segmentnull_HostUnknown.fa | B591 |
| AY597011_OrganismHuman_coronavirus_HKU1_Strain_NameHKU1_Segmentnull_HostHuman.fa | B592 |
| NC_006577_OrganismHuman_coronavirus_HKU1_Strain_NameHKU1_Segmentnull_HostHuman.fa | B593 |
| DQ415903_OrganismHuman_coronavirus_HKU1_Strain_NameN3_Segmentnull_HostUnknown.fa | B594 |
| MH687968_OrganismBetacoronavirus_sp._Strain_NameVZ_BetaCoV_16715_52_Segmentnull_HostRat.fa | B595 |
| MH687970_OrganismBetacoronavirus_sp._Strain_NameVZ_BetaCoV_20724_34_c12_Segmentnull_HostRat.fa | B596 |
| KM349743_OrganismBetacoronavirus_HKU24_Strain_NameHKU24_R05009I_Segmentnull_HostRat.fa | B597 |
| NC_026011_OrganismBetacoronavirus_HKU24_Strain_NameHKU24_R05005I_Segmentnull_HostRat.fa | B598 |
| KM349742_OrganismBetacoronavirus_HKU24_Strain_NameHKU24_R05005I_Segmentnull_HostRat.fa | B599 |
| KM349744_OrganismBetacoronavirus_HKU24_Strain_NameHKU24_R05010I_Segmentnull_HostRat.fa | B600 |
| MH687974_OrganismBetacoronavirus_sp._Strain_NameVZ_BetaCoV_20724_43_Segmentnull_HostRat.fa | B601 |
| MH687977_OrganismBetacoronavirus_sp._Strain_NameVZ_BetaCoV_22084_10_Segmentnull_HostRat.fa | B602 |
| MH687969_OrganismBetacoronavirus_sp._Strain_NameVZ_BetaCoV_20724_33_Segmentnull_HostRat.fa | B603 |
| MH687971_OrganismBetacoronavirus_sp._Strain_NameVZ_BetaCoV_20724_34_c13_Segmentnull_HostRat.fa | B604 |
| MH687976_OrganismBetacoronavirus_sp._Strain_NameVZ_BetaCoV_22084_1_Segmentnull_HostRat.fa | B605 |
| MH687972_OrganismBetacoronavirus_sp._Strain_NameVZ_BetaCoV_20724_38_Segmentnull_HostRat.fa | B606 |
| MH687973_OrganismBetacoronavirus_sp._Strain_NameVZ_BetaCoV_20724_39_Segmentnull_HostRat.fa | B607 |
| MH687978_OrganismBetacoronavirus_sp._Strain_NameVZ_BetaCoV_22084_6_Segmentnull_HostRat.fa | B608 |
| JX860640_OrganismCanine_respiratory_coronavirus_Strain_NameK37_Segmentnull_HostDog.fa | B609 |
| KX432213_OrganismCanine_respiratory_coronavirus_Strain_NameBJ232_Segmentnull_HostDog.fa | B610 |
| AB354579_OrganismBovine_coronavirus_Strain_NameKakegawa_Segmentnull_HostUnknown.fa | B611 |
| LQ289146_OrganismBovine_coronavirus_Strain_NameUNKNOWN_LQ289146_Segmentnull_HostUnknown.fa | B612 |
| AF220295_OrganismBovine_coronavirus_Strain_NameQuebec_Segmentnull_HostUnknown.fa | B613 |
| U00735_OrganismBovine_coronavirus_Strain_NameMebus_Segmentnull_HostUnknown.fa | B614 |
| MH810163_OrganismYak_coronavirus_Strain_NameYAK_HY24_CH_2017_Segmentnull_HostYak.fa | B615 |
| FJ415324_OrganismHuman_enteric_coronavirus_4408_Strain_Name4408_Segmentnull_HostHuman.fa | B616 |
| MG518518_OrganismWater_deer_coronavirus_Strain_NameW17_18_Segmentnull_HostUnknown.fa | B617 |
| DQ811784_OrganismBovine_coronavirus_DB2_Strain_NameDB2_Segmentnull_HostCattle.fa | B618 |
| FJ938063_OrganismBovine_coronavirus_E_DB2_TC_Strain_NameE_DB2_TC_Segmentnull_HostCattle.fa | B619 |
| FJ425185_OrganismWaterbuck_coronavirus_US_OH_WD358_GnC_1994_Strain_NameUNKNOWN_FJ425185_Segmentnull_HostAntelope.fa | B620 |
| FJ425184_OrganismWaterbuck_coronavirus_US_OH_WD358_TC_1994_Strain_NameUNKNOWN_FJ425184_Segmentnull_HostAntelope.fa | B621 |
| FJ425186_OrganismWaterbuck_coronavirus_US_OH_WD358_1994_Strain_NameUNKNOWN_FJ425186_Segmentnull_HostAntelope.fa | B622 |
| FJ425190_OrganismSambar_deer_coronavirus_US_OH_WD388_TC_1994_Strain_NameUNKNOWN_FJ425190_Segmentnull_HostDeer.fa | B623 |
| FJ425188_OrganismSambar_deer_coronavirus_US_OH_WD388_TC_1994_Strain_NameUNKNOWN_FJ425188_Segmentnull_HostDeer.fa | B624 |
| FJ425189_OrganismSambar_deer_coronavirus_US_OH_WD388_1994_Strain_NameUNKNOWN_FJ425189_Segmentnull_HostDeer.fa | B625 |
| EF424619_OrganismBovine_coronavirus_E_AH187_Strain_NameE_AH187_Segmentnull_HostCattle.fa | B626 |
| FJ425187_OrganismWhite_tailed_deer_coronavirus_US_OH_WD470_1994_Strain_NameUNKNOWN_FJ425187_Segmentnull_HostDeer.fa | B627 |
| FJ938066_OrganismBovine_respiratory_coronavirus_bovine_US_OH_440_TC_1996_Strain_Namebovine_US_OH_440_TC_1996_Segmentnull_HostCattle.fa | B628 |
| LC494161_OrganismBovine_coronavirus_Strain_NameTCG_5_Segmentnull_HostUnknown.fa | B629 |
| LC494172_OrganismBovine_coronavirus_Strain_NameTCG_6_Segmentnull_HostUnknown.fa | B630 |
| LC494134_OrganismBovine_coronavirus_Strain_NameIWT_6_Segmentnull_HostUnknown.fa | B631 |
| LC494154_OrganismBovine_coronavirus_Strain_NameSHG_1_Segmentnull_HostUnknown.fa | B632 |
| LC494155_OrganismBovine_coronavirus_Strain_NameSHG_2_Segmentnull_HostUnknown.fa | B633 |
| LC494136_OrganismBovine_coronavirus_Strain_NameIWT_8_Segmentnull_HostUnknown.fa | B634 |
| LC494135_OrganismBovine_coronavirus_Strain_NameIWT_7_Segmentnull_HostUnknown.fa | B635 |
| LC494185_OrganismBovine_coronavirus_Strain_NameTCG_26_Segmentnull_HostUnknown.fa | B636 |
| LC494184_OrganismBovine_coronavirus_Strain_NameTCG_25_Segmentnull_HostUnknown.fa | B637 |
| LC494188_OrganismBovine_coronavirus_Strain_NameTCG_29_Segmentnull_HostUnknown.fa | B638 |
| LC494190_OrganismBovine_coronavirus_Strain_NameTCG_31_Segmentnull_HostUnknown.fa | B639 |
| LC494191_OrganismBovine_coronavirus_Strain_NameTCG_32_Segmentnull_HostUnknown.fa | B640 |
| LC494183_OrganismBovine_coronavirus_Strain_NameTCG_24_Segmentnull_HostUnknown.fa | B641 |
| LC494187_OrganismBovine_coronavirus_Strain_NameTCG_28_Segmentnull_HostUnknown.fa | B642 |
| LC494192_OrganismBovine_coronavirus_Strain_NameTCG_33_Segmentnull_HostUnknown.fa | B643 |
| LC494186_OrganismBovine_coronavirus_Strain_NameTCG_27_Segmentnull_HostUnknown.fa | B644 |
| LC494189_OrganismBovine_coronavirus_Strain_NameTCG_30_Segmentnull_HostUnknown.fa | B645 |
| LC494153_OrganismBovine_coronavirus_Strain_NameIWT_27_Segmentnull_HostUnknown.fa | B646 |
| LC494180_OrganismBovine_coronavirus_Strain_NameTCG_21_Segmentnull_HostUnknown.fa | B647 |
| LC494182_OrganismBovine_coronavirus_Strain_NameTCG_23_Segmentnull_HostUnknown.fa | B648 |
| LC494181_OrganismBovine_coronavirus_Strain_NameTCG_22_Segmentnull_HostUnknown.fa | B649 |
| LC494150_OrganismBovine_coronavirus_Strain_NameIWT_24_Segmentnull_HostUnknown.fa | B650 |
| LC494148_OrganismBovine_coronavirus_Strain_NameIWT_22_Segmentnull_HostUnknown.fa | B651 |
| LC494152_OrganismBovine_coronavirus_Strain_NameIWT_26_Segmentnull_HostUnknown.fa | B652 |
| LC494151_OrganismBovine_coronavirus_Strain_NameIWT_25_Segmentnull_HostUnknown.fa | B653 |
| LC494147_OrganismBovine_coronavirus_Strain_NameIWT_17_Segmentnull_HostUnknown.fa | B654 |
| LC494149_OrganismBovine_coronavirus_Strain_NameIWT_23_Segmentnull_HostUnknown.fa | B655 |
| LC494179_OrganismBovine_coronavirus_Strain_NameTCG_20_Segmentnull_HostUnknown.fa | B656 |
| LC494178_OrganismBovine_coronavirus_Strain_NameTCG_19_Segmentnull_HostUnknown.fa | B657 |
| LC494126_OrganismBovine_coronavirus_Strain_NameGIF_1_Segmentnull_HostUnknown.fa | B658 |
| LC494158_OrganismBovine_coronavirus_Strain_NameSHG_5_Segmentnull_HostUnknown.fa | B659 |
| LC494128_OrganismBovine_coronavirus_Strain_NameIWT_10_Segmentnull_HostUnknown.fa | B660 |
| LC494177_OrganismBovine_coronavirus_Strain_NameTCG_9_Segmentnull_HostUnknown.fa | B661 |
| LC494127_OrganismBovine_coronavirus_Strain_NameIWT_9_Segmentnull_HostUnknown.fa | B662 |
| LC494157_OrganismBovine_coronavirus_Strain_NameSHG_4_Segmentnull_HostUnknown.fa | B663 |
| LC494142_OrganismBovine_coronavirus_Strain_NameIWT_18_Segmentnull_HostUnknown.fa | B664 |
| LC494143_OrganismBovine_coronavirus_Strain_NameIWT_19_Segmentnull_HostUnknown.fa | B665 |
| LC494144_OrganismBovine_coronavirus_Strain_NameIWT_20_Segmentnull_HostUnknown.fa | B666 |
| LC494145_OrganismBovine_coronavirus_Strain_NameIWT_21_Segmentnull_HostUnknown.fa | B667 |
| LC494146_OrganismBovine_coronavirus_Strain_NameIWT_16_Segmentnull_HostUnknown.fa | B668 |
| LC494141_OrganismBovine_coronavirus_Strain_NameIWT_15_Segmentnull_HostUnknown.fa | B669 |
| LC494176_OrganismBovine_coronavirus_Strain_NameTCG_17_Segmentnull_HostUnknown.fa | B670 |
| LC494175_OrganismBovine_coronavirus_Strain_NameTCG_18_Segmentnull_HostUnknown.fa | B671 |
| LC494159_OrganismBovine_coronavirus_Strain_NameSHG_6_Segmentnull_HostUnknown.fa | B672 |
| LC494137_OrganismBovine_coronavirus_Strain_NameIWT_11_Segmentnull_HostUnknown.fa | B673 |
| LC494129_OrganismBovine_coronavirus_Strain_NameIWT_1_Segmentnull_HostUnknown.fa | B674 |
| LC494165_OrganismBovine_coronavirus_Strain_NameTCG_10_Segmentnull_HostUnknown.fa | B675 |
| LC494168_OrganismBovine_coronavirus_Strain_NameTCG_13_Segmentnull_HostUnknown.fa | B676 |
| LC494171_OrganismBovine_coronavirus_Strain_NameTCG_16_Segmentnull_HostUnknown.fa | B677 |
| LC494170_OrganismBovine_coronavirus_Strain_NameTCG_15_Segmentnull_HostUnknown.fa | B678 |
| LC494132_OrganismBovine_coronavirus_Strain_NameIWT_4_Segmentnull_HostUnknown.fa | B679 |
| LC494131_OrganismBovine_coronavirus_Strain_NameIWT_3_Segmentnull_HostUnknown.fa | B680 |
| LC494164_OrganismBovine_coronavirus_Strain_NameTCG_1_Segmentnull_HostUnknown.fa | B681 |
| LC494174_OrganismBovine_coronavirus_Strain_NameTCG_8_Segmentnull_HostUnknown.fa | B682 |
| LC494139_OrganismBovine_coronavirus_Strain_NameIWT_13_Segmentnull_HostUnknown.fa | B683 |
| LC494162_OrganismBovine_coronavirus_Strain_NameTCG_3_Segmentnull_HostUnknown.fa | B684 |
| LC494163_OrganismBovine_coronavirus_Strain_NameTCG_2_Segmentnull_HostUnknown.fa | B685 |
| LC494133_OrganismBovine_coronavirus_Strain_NameIWT_5_Segmentnull_HostUnknown.fa | B686 |
| LC494130_OrganismBovine_coronavirus_Strain_NameIWT_2_Segmentnull_HostUnknown.fa | B687 |
| LC494167_OrganismBovine_coronavirus_Strain_NameTCG_12_Segmentnull_HostUnknown.fa | B688 |
| LC494166_OrganismBovine_coronavirus_Strain_NameTCG_11_Segmentnull_HostUnknown.fa | B689 |
| LC494169_OrganismBovine_coronavirus_Strain_NameTCG_14_Segmentnull_HostUnknown.fa | B690 |
| LC494156_OrganismBovine_coronavirus_Strain_NameSHG_3_Segmentnull_HostUnknown.fa | B691 |
| LC494160_OrganismBovine_coronavirus_Strain_NameTCG_4_Segmentnull_HostUnknown.fa | B692 |
| LC494173_OrganismBovine_coronavirus_Strain_NameTCG_7_Segmentnull_HostUnknown.fa | B693 |
| LC494140_OrganismBovine_coronavirus_Strain_NameIWT_14_Segmentnull_HostUnknown.fa | B694 |
| LC494138_OrganismBovine_coronavirus_Strain_NameIWT_12_Segmentnull_HostUnknown.fa | B695 |
| EF424616_OrganismBovine_coronavirus_E_AH65_TC_Strain_NameE_AH65_TC_Segmentnull_HostCattle.fa | B696 |
| EF424617_OrganismBovine_coronavirus_R_AH65_Strain_NameR_AH65_Segmentnull_HostCattle.fa | B697 |
| EF424618_OrganismBovine_coronavirus_R_AH65_TC_Strain_NameR_AH65_TC_Segmentnull_HostCattle.fa | B698 |
| KU886219_OrganismBovine_coronavirus_Strain_NameBCV_AKS_01_Segmentnull_HostCattle.fa | B699 |
| MH043953_OrganismBovine_coronavirus_Strain_Name4_17_25_Segmentnull_HostCattle.fa | B700 |
| MH043952_OrganismBovine_coronavirus_Strain_Name4_17_03_Segmentnull_HostCattle.fa | B701 |
| MH043955_OrganismBovine_coronavirus_Strain_Name7_16_23_Segmentnull_HostCattle.fa | B702 |
| MH043954_OrganismBovine_coronavirus_Strain_Name4_17_08_Segmentnull_HostCattle.fa | B703 |
| EF424621_OrganismSable_antelope_coronavirus_US_OH1_2003_Strain_NameUS_OH1_2003_Segmentnull_HostAntelope.fa | B704 |
| EF424623_OrganismGiraffe_coronavirus_US_OH3_2003_Strain_NameUS_OH3_2003_Segmentnull_HostGiraffe.fa | B705 |
| EF424624_OrganismCalf_giraffe_coronavirus_US_OH3_2006_Strain_NameUS_OH3_2006_Segmentnull_HostGiraffe.fa | B706 |
| EF424622_OrganismGiraffe_coronavirus_US_OH3_TC_2006_Strain_NameUS_OH3_TC_2006_Segmentnull_HostGiraffe.fa | B707 |
| EF424615_OrganismBovine_coronavirus_E_AH65_Strain_NameE_AH65_Segmentnull_HostCattle.fa | B708 |
| AF391542_OrganismBovine_coronavirus_Strain_NameBCoV_LUN_Segmentnull_HostUnknown.fa | B709 |
| FJ938065_OrganismBovine_respiratory_coronavirus_AH187_Strain_NameAH187_Segmentnull_HostCattle.fa | B710 |
| EF424620_OrganismBovine_coronavirus_R_AH187_Strain_NameR_AH187_Segmentnull_HostCattle.fa | B711 |
| FJ938064_OrganismBovine_coronavirus_E_AH187_TC_Strain_NameE_AH187_TC_Segmentnull_HostCattle.fa | B712 |
| NC_003045_OrganismBovine_coronavirus_Strain_NameBCoV_ENT_Segmentnull_HostUnknown.fa | B713 |
| AF391541_OrganismBovine_coronavirus_Strain_NameBCoV_ENT_Segmentnull_HostUnknown.fa | B714 |
| DQ915164_OrganismBovine_coronavirus_isolate_Alpaca_Strain_NameUNKNOWN_DQ915164_Segmentnull_SubtypeBovine_HostAlpaca.fa | B715 |
| KU558922_OrganismBetacoronavirus_1_Strain_NameBuffalo_coronavirus_B1_24F_Segmentnull_HostBuffalo.fa | B716 |
| KU558923_OrganismBetacoronavirus_1_Strain_NameBuffalo_coronavirus_B1_28F_Segmentnull_HostBuffalo.fa | B717 |
| KX982264_OrganismBovine_coronavirus_Strain_NameBCoV_2014_13_Segmentnull_HostCattle.fa | B718 |
| MG757138_OrganismBovine_coronavirus_Strain_NameICSA21_LBA_Segmentnull_HostCattle.fa | B719 |
| MG757142_OrganismBovine_coronavirus_Strain_NameICSA_pool_LBA_Segmentnull_HostCattle.fa | B720 |
| MG757139_OrganismBovine_coronavirus_Strain_NameICSA16_EN_Segmentnull_HostCattle.fa | B721 |
| MG757140_OrganismBovine_coronavirus_Strain_NameICSA16_LBA_Segmentnull_HostCattle.fa | B722 |
| MG757141_OrganismBovine_coronavirus_Strain_NameICSA_pool_EN_Segmentnull_HostCattle.fa | B723 |
| KF906251_OrganismDromedary_camel_coronavirus_HKU23_Strain_NameHKU23_368F_Segmentnull_HostCamel.fa | B724 |
| KF906250_OrganismDromedary_camel_coronavirus_HKU23_Strain_NameHKU23_362F_Segmentnull_HostCamel.fa | B725 |
| KF906249_OrganismDromedary_camel_coronavirus_HKU23_Strain_NameHKU23_265F_Segmentnull_HostCamel.fa | B726 |
| MN514966_OrganismDromedary_camel_coronavirus_HKU23_Strain_NameDcCoV_HKU23_camel_Nigeria_NV1097_2015_Segmentnull_HostCamel.fa | B727 |
| MN514967_OrganismDromedary_camel_coronavirus_HKU23_Strain_NameDcCoV_HKU23_camel_Nigeria_NV1385_2016_Segmentnull_HostCamel.fa | B728 |
| MN514964_OrganismDromedary_camel_coronavirus_HKU23_Strain_NameDcCoV_HKU23_camel_Nigeria_NV1010_2015_Segmentnull_HostCamel.fa | B729 |
| MN514965_OrganismDromedary_camel_coronavirus_HKU23_Strain_NameDcCoV_HKU23_camel_Nigeria_NV1092_2015_Segmentnull_HostCamel.fa | B730 |
| MN514962_OrganismDromedary_camel_coronavirus_HKU23_Strain_NameDcCoV_HKU23_camel_Ethiopia_CAC1019_2015_Segmentnull_HostCamel.fa | B731 |
| MN514963_OrganismDromedary_camel_coronavirus_HKU23_Strain_NameDcCoV_HKU23_camel_Morocco_CAC2586_2016_Segmentnull_HostCamel.fa | B732 |
| KF530092_OrganismHuman_coronavirus_OC43_Strain_NameOC43_human_USA_008_5_2000_Segmentnull_HostHuman.fa | B733 |
| KY014281_OrganismHuman_coronavirus_OC43_Strain_Name2002_04_Segmentnull_HostHuman.fa | B734 |
| KF530080_OrganismHuman_coronavirus_OC43_Strain_NameOC43_human_USA_9712_31_1997_Segmentnull_HostHuman.fa | B735 |
| KF530072_OrganismHuman_coronavirus_OC43_Strain_NameOC43_human_USA_9712_13_1997_Segmentnull_HostHuman.fa | B736 |
| KF530064_OrganismHuman_coronavirus_OC43_Strain_NameOC43_human_USA_9612_9_1996_Segmentnull_HostHuman.fa | B737 |
| KF530078_OrganismHuman_coronavirus_OC43_Strain_NameOC43_human_USA_9612_29_1996_Segmentnull_HostHuman.fa | B738 |
| KY014282_OrganismHuman_coronavirus_OC43_Strain_Name2007_09_Segmentnull_HostHuman.fa | B739 |
| KU131570_OrganismHuman_coronavirus_OC43_Strain_NameHCoV_OC43_UK_London_2011_Segmentnull_HostHuman.fa | B740 |
| KY967360_OrganismHuman_coronavirus_OC43_Strain_NameHCoV_OC43_Seattle_USA_SC2476_2015_Segmentnull_HostHuman.fa | B741 |
| MF314143_OrganismHuman_coronavirus_OC43_Strain_NameHCoV_OC43_USA_ACRI_0052_2016_Segmentnull_HostHuman.fa | B742 |
| KP198611_OrganismHuman_coronavirus_OC43_Strain_Name1783A_10_Segmentnull_HostHuman.fa | B743 |
| KP198610_OrganismHuman_coronavirus_OC43_Strain_Name2058A_10_Segmentnull_HostHuman.fa | B744 |
| KF923906_OrganismHuman_coronavirus_OC43_Strain_Name3194A_2012_Segmentnull_HostHuman.fa | B745 |
| KF923896_OrganismHuman_coronavirus_OC43_Strain_Name3074A_2012_Segmentnull_HostHuman.fa | B746 |
| KF530079_OrganismHuman_coronavirus_OC43_Strain_NameOC43_human_USA_913_29_1991_Segmentnull_HostHuman.fa | B747 |
| KF530094_OrganismHuman_coronavirus_OC43_Strain_NameOC43_human_USA_912_36_1991_Segmentnull_HostHuman.fa | B748 |
| KF530082_OrganismHuman_coronavirus_OC43_Strain_NameOC43_human_USA_912_11_1991_Segmentnull_HostHuman.fa | B749 |
| KF530089_OrganismHuman_coronavirus_OC43_Strain_NameOC43_human_USA_911_66_1991_Segmentnull_HostHuman.fa | B750 |
| KF530088_OrganismHuman_coronavirus_OC43_Strain_NameOC43_human_USA_901_54_1990_Segmentnull_HostHuman.fa | B751 |
| KF530091_OrganismHuman_coronavirus_OC43_Strain_NameOC43_human_USA_911_58_1991_Segmentnull_HostHuman.fa | B752 |
| KF530076_OrganismHuman_coronavirus_OC43_Strain_NameOC43_human_USA_911_11_1991_Segmentnull_HostHuman.fa | B753 |
| KF530071_OrganismHuman_coronavirus_OC43_Strain_NameOC43_human_USA_925_1_1992_Segmentnull_HostHuman.fa | B754 |
| KF530067_OrganismHuman_coronavirus_OC43_Strain_NameOC43_human_USA_912_10_1991_Segmentnull_HostHuman.fa | B755 |
| KF530096_OrganismHuman_coronavirus_OC43_Strain_NameOC43_human_USA_911_38_1991_Segmentnull_HostHuman.fa | B756 |
| KF530095_OrganismHuman_coronavirus_OC43_Strain_NameOC43_human_USA_912_6_1991_Segmentnull_HostHuman.fa | B757 |
| KF530084_OrganismHuman_coronavirus_OC43_Strain_NameOC43_human_USA_951_18_1995_Segmentnull_HostHuman.fa | B758 |
| KF530098_OrganismHuman_coronavirus_OC43_Strain_NameOC43_human_USA_965_6_1996_Segmentnull_HostHuman.fa | B759 |
| KF530069_OrganismHuman_coronavirus_OC43_Strain_NameOC43_human_USA_982_4_1998_Segmentnull_HostHuman.fa | B760 |
| KF530099_OrganismHuman_coronavirus_OC43_Strain_NameOC43_human_USA_971_5_1997_Segmentnull_HostHuman.fa | B761 |
| KF530063_OrganismHuman_coronavirus_OC43_Strain_NameOC43_human_USA_9612_48_1996_Segmentnull_HostHuman.fa | B762 |
| MG762674_OrganismRousettus_bat_coronavirus_HKU9_Strain_NameRousettus_spp_Jinghong_2009_Segmentnull_HostBat.fa | B763 |
| EF065515_OrganismBat_coronavirus_HKU9_3_Strain_NameBF_493I_Segmentnull_HostBat.fa | B764 |
| HM211098_OrganismBat_coronavirus_HKU9_5_1_Strain_NameUNKNOWN_HM211098_Segmentnull_HostBat.fa | B765 |
| HM211100_OrganismBat_coronavirus_HKU9_10_1_Strain_NameUNKNOWN_HM211100_Segmentnull_HostBat.fa | B766 |
| NC_009021_OrganismRousettus_bat_coronavirus_HKU9_Strain_NameHKU9_1_BF_005I_Segmentnull_HostBat.fa | B767 |
| EF065513_OrganismBat_coronavirus_HKU9_1_Strain_NameBF_005I_Segmentnull_HostBat.fa | B768 |
| EF065516_OrganismBat_coronavirus_HKU9_4_Strain_NameBF_141I_Segmentnull_HostBat.fa | B769 |
| HM211099_OrganismBat_coronavirus_HKU9_5_2_Strain_NameUNKNOWN_HM211099_Segmentnull_HostBat.fa | B770 |
| EF065514_OrganismBat_coronavirus_HKU9_2_Strain_NameBF_017I_Segmentnull_HostBat.fa | B771 |
| HM211101_OrganismBat_coronavirus_HKU9_10_2_Strain_NameUNKNOWN_HM211101_Segmentnull_HostBat.fa | B772 |
| MK211379_OrganismCoronavirus_BtRt_BetaCoV_GX2018_Strain_NameBtRt_BetaCoV_GX2018_Segmentnull_HostBat.fa | B773 |
| FV537210_OrganismSARS_coronavirus_Strain_NameModified_Microbial_Nucleic_Acid_Segmentnull_HostUnknown.fa | B774 |
| FV537211_OrganismSARS_coronavirus_Strain_NameModified_Microbial_Nucleic_Acid_Segmentnull_HostUnknown.fa | B775 |
| KF636752_OrganismBat_Hp_betacoronavirus_Zhejiang2013_Strain_NameZhejiang2013_Segmentnull_HostBat.fa | B776 |
| NC_025217_OrganismBat_Hp_betacoronavirus_Zhejiang2013_Strain_NameZhejiang2013_Segmentnull_HostBat.fa | B777 |
| KY352407_OrganismSevere_acute_respiratory_syndrome_related_coronavirus_Strain_NameBtKY72_Segmentnull_HostBat.fa | B778 |
| MG772933_OrganismBat_SARS_like_coronavirus_Strain_Namebat_SL_CoVZC45_Segmentnull_HostBat.fa | B779 |
| MG772934_OrganismBat_SARS_like_coronavirus_Strain_Namebat_SL_CoVZXC21_Segmentnull_HostBat.fa | B780 |
| MT084071_OrganismPangolin_coronavirus_Strain_NameMP789_Segmentnull_HostUnknown.fa | B781 |
| MT106054_OrganismSevere_acute_respiratory_syndrome_coronavirus_2_Strain_Name2019_nCoV_USA_TX1_2020_Segmentnull_HostHuman.fa | B782 |
| MT135042_OrganismSevere_acute_respiratory_syndrome_coronavirus_2_Strain_NameSARS_CoV_2_231_human_2020_CHN_Segmentnull_HostHuman.fa | B783 |
| MT135041_OrganismSevere_acute_respiratory_syndrome_coronavirus_2_Strain_NameSARS_CoV_2_105_human_2020_CHN_Segmentnull_HostHuman.fa | B784 |
| MT135044_OrganismSevere_acute_respiratory_syndrome_coronavirus_2_Strain_NameSARS_CoV_2_235_human_2020_CHN_Segmentnull_HostHuman.fa | B785 |
| MT135043_OrganismSevere_acute_respiratory_syndrome_coronavirus_2_Strain_NameSARS_CoV_2_233_human_2020_CHN_Segmentnull_HostHuman.fa | B786 |
| MT118835_OrganismSevere_acute_respiratory_syndrome_coronavirus_2_Strain_Name2019_nCoV_USA_CA9_2020_Segmentnull_HostHuman.fa | B787 |
| MT039887_OrganismSevere_acute_respiratory_syndrome_coronavirus_2_Strain_Name2019_nCoV_USA_WI1_2020_Segmentnull_HostHuman.fa | B788 |
| MT123291_OrganismSevere_acute_respiratory_syndrome_coronavirus_2_Strain_NameSARS_CoV_2_IQTC02_human_2020_CHN_Segmentnull_HostHuman.fa | B789 |
| MT123293_OrganismSevere_acute_respiratory_syndrome_related_coronavirus_Strain_NameSARS_CoV_2_IQTC03_human_2020_CHN_Segmentnull_HostHuman.fa | B790 |
| MT012098_OrganismSevere_acute_respiratory_syndrome_coronavirus_2_Strain_NameSARS_CoV_2_29_human_2020_IND_Segmentnull_HostHuman.fa | B791 |
| MT159722_OrganismSevere_acute_respiratory_syndrome_coronavirus_2_Strain_Name2019_nCoV_USA_CruiseA_6_2020_Segmentnull_HostHuman.fa | B792 |
| MT159705_OrganismSevere_acute_respiratory_syndrome_coronavirus_2_Strain_Name2019_nCoV_USA_CruiseA_7_2020_Segmentnull_HostHuman.fa | B793 |
| MT159707_OrganismSevere_acute_respiratory_syndrome_coronavirus_2_Strain_Name2019_nCoV_USA_CruiseA_10_2020_Segmentnull_HostHuman.fa | B794 |
| MT027064_OrganismSevere_acute_respiratory_syndrome_coronavirus_2_Strain_Name2019_nCoV_USA_CA5_2020_Segmentnull_HostHuman.fa | B795 |
| MT044258_OrganismSevere_acute_respiratory_syndrome_coronavirus_2_Strain_Name2019_nCoV_USA_CA6_2020_Segmentnull_HostHuman.fa | B796 |
| MT159711_OrganismSevere_acute_respiratory_syndrome_coronavirus_2_Strain_Name2019_nCoV_USA_CruiseA_13_2020_Segmentnull_HostHuman.fa | B797 |
| MT159721_OrganismSevere_acute_respiratory_syndrome_coronavirus_2_Strain_Name2019_nCoV_USA_CruiseA_5_2020_Segmentnull_HostHuman.fa | B798 |
| MT159714_OrganismSevere_acute_respiratory_syndrome_coronavirus_2_Strain_Name2019_nCoV_USA_CruiseA_16_2020_Segmentnull_HostHuman.fa | B799 |
| MN988668_OrganismSevere_acute_respiratory_syndrome_coronavirus_2_Strain_Name2019_nCoV_WHU01_Segmentnull_HostHuman.fa | B800 |
| MT159719_OrganismSevere_acute_respiratory_syndrome_coronavirus_2_Strain_Name2019_nCoV_USA_CruiseA_3_2020_Segmentnull_HostHuman.fa | B801 |
| MT159713_OrganismSevere_acute_respiratory_syndrome_coronavirus_2_Strain_Name2019_nCoV_USA_CruiseA_15_2020_Segmentnull_HostHuman.fa | B802 |
| MT159706_OrganismSevere_acute_respiratory_syndrome_coronavirus_2_Strain_Name2019_nCoV_USA_CruiseA_8_2020_Segmentnull_HostHuman.fa | B803 |
| MN988669_OrganismSevere_acute_respiratory_syndrome_coronavirus_2_Strain_Name2019_nCoV_WHU02_Segmentnull_HostHuman.fa | B804 |
| MT159710_OrganismSevere_acute_respiratory_syndrome_coronavirus_2_Strain_Name2019_nCoV_USA_CruiseA_9_2020_Segmentnull_HostHuman.fa | B805 |
| MT159716_OrganismSevere_acute_respiratory_syndrome_coronavirus_2_Strain_Name2019_nCoV_USA_CruiseA_18_2020_Segmentnull_HostHuman.fa | B806 |
| MT159709_OrganismSevere_acute_respiratory_syndrome_coronavirus_2_Strain_Name2019_nCoV_USA_CruiseA_12_2020_Segmentnull_HostHuman.fa | B807 |
| MT159720_OrganismSevere_acute_respiratory_syndrome_coronavirus_2_Strain_Name2019_nCoV_USA_CruiseA_4_2020_Segmentnull_HostHuman.fa | B808 |
| MT019530_OrganismSevere_acute_respiratory_syndrome_coronavirus_2_Strain_NameBetaCoV_Wuhan_IPBCAMS_WH_02_2019_Segmentnull_HostHuman.fa | B809 |
| MT159718_OrganismSevere_acute_respiratory_syndrome_coronavirus_2_Strain_Name2019_nCoV_USA_CruiseA_2_2020_Segmentnull_HostHuman.fa | B810 |
| MN996528_OrganismSevere_acute_respiratory_syndrome_coronavirus_2_Strain_NameWIV04_Segmentnull_HostHuman.fa | B811 |
| MT019532_OrganismSevere_acute_respiratory_syndrome_coronavirus_2_Strain_NameBetaCoV_Wuhan_IPBCAMS_WH_04_2019_Segmentnull_HostHuman.fa | B812 |
| MN908947_OrganismSevere_acute_respiratory_syndrome_coronavirus_2_Strain_NameWuhan_Hu_1_Segmentnull_HostHuman.fa | B813 |
| LR757998_OrganismSevere_acute_respiratory_syndrome_coronavirus_2_Strain_NameUNKNOWN_LR757998_Segmentnull_HostHuman.fa | B814 |
| LR757996_OrganismSevere_acute_respiratory_syndrome_coronavirus_2_Strain_NameUNKNOWN_LR757996_Segmentnull_HostHuman.fa | B815 |
| LC521925_OrganismSevere_acute_respiratory_syndrome_coronavirus_2_Strain_Name2019_nCoV_Japan_AI_I_004_2020_Segmentnull_HostHuman.fa | B816 |
| MT066176_OrganismSevere_acute_respiratory_syndrome_coronavirus_2_Strain_NameSARS_CoV_2_NTU02_2020_TWN_Segmentnull_HostHuman.fa | B817 |
| MN996529_OrganismSevere_acute_respiratory_syndrome_coronavirus_2_Strain_NameWIV05_Segmentnull_HostHuman.fa | B818 |
| MN996527_OrganismSevere_acute_respiratory_syndrome_coronavirus_2_Strain_NameWIV02_Segmentnull_HostHuman.fa | B819 |
| MN996531_OrganismSevere_acute_respiratory_syndrome_coronavirus_2_Strain_NameWIV07_Segmentnull_HostHuman.fa | B820 |
| MN996530_OrganismSevere_acute_respiratory_syndrome_coronavirus_2_Strain_NameWIV06_Segmentnull_HostHuman.fa | B821 |
| LC528232_OrganismSevere_acute_respiratory_syndrome_coronavirus_2_Strain_NameSARS_CoV_2_Hu_DP_Kng_19_020_Segmentnull_HostHuman.fa | B822 |
| MT093631_OrganismSevere_acute_respiratory_syndrome_coronavirus_2_Strain_NameSARS_CoV_2_WH_09_human_2020_CHN_Segmentnull_HostHuman.fa | B823 |
| LC528233_OrganismSevere_acute_respiratory_syndrome_coronavirus_2_Strain_NameSARS_CoV_2_Hu_DP_Kng_19_027_Segmentnull_HostHuman.fa | B824 |
| MT072688_OrganismSevere_acute_respiratory_syndrome_coronavirus_2_Strain_NameSARS0CoV_2_61_TW_human_2020__NPL_Segmentnull_HostHuman.fa | B825 |
| MT039873_OrganismSevere_acute_respiratory_syndrome_coronavirus_2_Strain_NameHZ_1_Segmentnull_HostHuman.fa | B826 |
| MT106053_OrganismSevere_acute_respiratory_syndrome_coronavirus_2_Strain_Name2019_nCoV_USA_CA8_2020_Segmentnull_HostHuman.fa | B827 |
| MT159712_OrganismSevere_acute_respiratory_syndrome_coronavirus_2_Strain_Name2019_nCoV_USA_CruiseA_14_2020_Segmentnull_HostHuman.fa | B828 |
| MT159708_OrganismSevere_acute_respiratory_syndrome_coronavirus_2_Strain_Name2019_nCoV_USA_CruiseA_11_2020_Segmentnull_HostHuman.fa | B829 |
| MT027063_OrganismSevere_acute_respiratory_syndrome_coronavirus_2_Strain_Name2019_nCoV_USA_CA4_2020_Segmentnull_HostHuman.fa | B830 |
| MT027062_OrganismSevere_acute_respiratory_syndrome_coronavirus_2_Strain_Name2019_nCoV_USA_CA3_2020_Segmentnull_HostHuman.fa | B831 |
| MT039888_OrganismSevere_acute_respiratory_syndrome_coronavirus_2_Strain_Name2019_nCoV_USA_MA1_2020_Segmentnull_HostHuman.fa | B832 |
| MT019533_OrganismSevere_acute_respiratory_syndrome_coronavirus_2_Strain_NameBetaCoV_Wuhan_IPBCAMS_WH_05_2020_Segmentnull_HostHuman.fa | B833 |
| MT159715_OrganismSevere_acute_respiratory_syndrome_coronavirus_2_Strain_Name2019_nCoV_USA_CruiseA_17_2020_Segmentnull_HostHuman.fa | B834 |
| MT019531_OrganismSevere_acute_respiratory_syndrome_coronavirus_2_Strain_NameBetaCoV_Wuhan_IPBCAMS_WH_03_2019_Segmentnull_HostHuman.fa | B835 |
| MT007544_OrganismSevere_acute_respiratory_syndrome_coronavirus_2_Strain_NameAustralia_VIC01_2020_Segmentnull_HostHuman.fa | B836 |
| MT039890_OrganismSevere_acute_respiratory_syndrome_coronavirus_2_Strain_NameSNU01_Segmentnull_HostHuman.fa | B837 |
| MN994468_OrganismSevere_acute_respiratory_syndrome_coronavirus_2_Strain_Name2019_nCoV_USA_CA2_2020_Segmentnull_HostHuman.fa | B838 |
| MT126808_OrganismSevere_acute_respiratory_syndrome_coronavirus_2_Strain_NameSARS_CoV_2_SP02_human_2020_BRA_Segmentnull_HostHuman.fa | B839 |
| MT066156_OrganismSevere_acute_respiratory_syndrome_coronavirus_2_Strain_NameSARS_CoV_2_INMI1_human_2020_ITA_Segmentnull_HostHuman.fa | B840 |
| MT093571_OrganismSevere_acute_respiratory_syndrome_coronavirus_2_Strain_NameSARS_CoV_2_01_human_2020_SWE_Segmentnull_HostHuman.fa | B841 |
| MT163716_OrganismSevere_acute_respiratory_syndrome_coronavirus_2_Strain_NameSARS_CoV_2_WA3_UW1_human_2020_USA_Segmentnull_HostHuman.fa | B842 |
| MT019529_OrganismSevere_acute_respiratory_syndrome_coronavirus_2_Strain_NameBetaCoV_Wuhan_IPBCAMS_WH_01_2019_Segmentnull_HostHuman.fa | B843 |
| MT159717_OrganismSevere_acute_respiratory_syndrome_coronavirus_2_Strain_Name2019_nCoV_USA_CruiseA_1_2020_Segmentnull_HostHuman.fa | B844 |
| MT121215_OrganismSevere_acute_respiratory_syndrome_coronavirus_2_Strain_NameSARS_CoV_2_SH01_human_2020_CHN_Segmentnull_HostHuman.fa | B845 |
| MT123290_OrganismSevere_acute_respiratory_syndrome_coronavirus_2_Strain_NameSARS_CoV_2_IQTC01_human_2020_CHN_Segmentnull_HostHuman.fa | B846 |
| LC529905_OrganismSevere_acute_respiratory_syndrome_coronavirus_2_Strain_NameTKYE6182_2020_Segmentnull_HostHuman.fa | B847 |
| LC522972_OrganismSevere_acute_respiratory_syndrome_coronavirus_2_Strain_Name2019_nCoV_Japan_KY_V_029_2020_Segmentnull_HostHuman.fa | B848 |
| MN975262_OrganismSevere_acute_respiratory_syndrome_coronavirus_2_Strain_Name2019_nCoV_HKU_SZ_005b_2020_Segmentnull_HostHuman.fa | B849 |
| LR757997_OrganismSevere_acute_respiratory_syndrome_coronavirus_2_Strain_NameUNKNOWN_LR757997_Segmentnull_HostHuman.fa | B850 |
| MN985325_OrganismSevere_acute_respiratory_syndrome_coronavirus_2_Strain_Name2019_nCoV_USA_WA1_2020_Segmentnull_HostHuman.fa | B851 |
| MT020881_OrganismSevere_acute_respiratory_syndrome_coronavirus_2_Strain_Name2019_nCoV_USA_WA1_F6_2020_Segmentnull_HostHuman.fa | B852 |
| MT020880_OrganismSevere_acute_respiratory_syndrome_coronavirus_2_Strain_Name2019_nCoV_USA_WA1_A12_2020_Segmentnull_HostHuman.fa | B853 |
| LC522973_OrganismSevere_acute_respiratory_syndrome_coronavirus_2_Strain_Name2019_nCoV_Japan_TY_WK_012_2020_Segmentnull_HostHuman.fa | B854 |
| LC522974_OrganismSevere_acute_respiratory_syndrome_coronavirus_2_Strain_Name2019_nCoV_Japan_TY_WK_501_2020_Segmentnull_HostHuman.fa | B855 |
| MT066175_OrganismSevere_acute_respiratory_syndrome_coronavirus_2_Strain_NameSARS_CoV_2_NTU01_2020_TWN_Segmentnull_HostHuman.fa | B856 |
| MT123292_OrganismSevere_acute_respiratory_syndrome_related_coronavirus_Strain_NameSARS_CoV_2_IQTC04_human_2020_CHN_Segmentnull_HostHuman.fa | B857 |
| MT050493_OrganismSevere_acute_respiratory_syndrome_coronavirus_2_Strain_NameSARS_CoV_2_166_human_2020_IND_Segmentnull_HostHuman.fa | B858 |
| MN988713_OrganismSevere_acute_respiratory_syndrome_coronavirus_2_Strain_Name2019_nCoV_USA_IL1_2020_Segmentnull_HostHuman.fa | B859 |
| MT044257_OrganismSevere_acute_respiratory_syndrome_coronavirus_2_Strain_Name2019_nCoV_USA_IL2_2020_Segmentnull_HostHuman.fa | B860 |
| MN994467_OrganismSevere_acute_respiratory_syndrome_coronavirus_2_Strain_Name2019_nCoV_USA_CA1_2020_Segmentnull_HostHuman.fa | B861 |
| LC522975_OrganismSevere_acute_respiratory_syndrome_coronavirus_2_Strain_Name2019_nCoV_Japan_TY_WK_521_2020_Segmentnull_HostHuman.fa | B862 |
| MT106052_OrganismSevere_acute_respiratory_syndrome_coronavirus_2_Strain_Name2019_nCoV_USA_CA7_2020_Segmentnull_HostHuman.fa | B863 |
| MN938384_OrganismSevere_acute_respiratory_syndrome_coronavirus_2_Strain_Name2019_nCoV_HKU_SZ_002a_2020_Segmentnull_HostHuman.fa | B864 |
| LR757995_OrganismSevere_acute_respiratory_syndrome_coronavirus_2_Strain_NameUNKNOWN_LR757995_Segmentnull_HostHuman.fa | B865 |
| MT163719_OrganismSevere_acute_respiratory_syndrome_coronavirus_2_Strain_NameSARS_CoV_2_WA7_UW4_human_2020_USA_Segmentnull_HostHuman.fa | B866 |
| MT163718_OrganismSevere_acute_respiratory_syndrome_coronavirus_2_Strain_NameSARS_CoV_2_WA6_UW3_human_2020_USA_Segmentnull_HostHuman.fa | B867 |
| MT163717_OrganismSevere_acute_respiratory_syndrome_coronavirus_2_Strain_NameSARS_CoV_2_WA4_UW2_human_2020_USA_Segmentnull_HostHuman.fa | B868 |
| MT152824_OrganismSevere_acute_respiratory_syndrome_coronavirus_2_Strain_NameSARS_CoV_2_WA2_human_2020_USA_Segmentnull_HostHuman.fa | B869 |
| MT049951_OrganismSevere_acute_respiratory_syndrome_coronavirus_2_Strain_NameSARS_CoV_2_Yunnan_01_human_2020_CHN_Segmentnull_HostHuman.fa | B870 |
| MN997409_OrganismSevere_acute_respiratory_syndrome_coronavirus_2_Strain_Name2019_nCoV_USA_AZ1_2020_Segmentnull_HostHuman.fa | B871 |
| KF294457_OrganismSARS_related_bat_coronavirus_Strain_NameLongquan_140_Segmentnull_HostBat.fa | B872 |
| GQ153542_OrganismBat_SARS_coronavirus_HKU3_7_Strain_NameHKU3_7_Segmentnull_HostUnknown.fa | B873 |
| GQ153543_OrganismBat_SARS_coronavirus_HKU3_8_Strain_NameHKU3_8_Segmentnull_HostUnknown.fa | B874 |
| GQ153547_OrganismBat_SARS_coronavirus_HKU3_12_Strain_NameHKU3_12_Segmentnull_HostUnknown.fa | B875 |
| DQ084200_OrganismBat_SARS_coronavirus_HKU3_3_Strain_NameHKU3_3_Segmentnull_HostUnknown.fa | B876 |
| DQ022305_OrganismBat_SARS_coronavirus_HKU3_1_Strain_NameHKU3_1_Segmentnull_HostBat.fa | B877 |
| DQ084199_OrganismBat_SARS_coronavirus_HKU3_2_Strain_NameHKU3_2_Segmentnull_HostUnknown.fa | B878 |
| GQ153539_OrganismBat_SARS_coronavirus_HKU3_4_Strain_NameHKU3_4_Segmentnull_HostUnknown.fa | B879 |
| GQ153541_OrganismBat_SARS_coronavirus_HKU3_6_Strain_NameHKU3_6_Segmentnull_HostUnknown.fa | B880 |
| GQ153548_OrganismBat_SARS_coronavirus_HKU3_13_Strain_NameHKU3_13_Segmentnull_HostUnknown.fa | B881 |
| GQ153545_OrganismBat_SARS_coronavirus_HKU3_10_Strain_NameHKU3_10_Segmentnull_HostUnknown.fa | B882 |
| GQ153544_OrganismBat_SARS_coronavirus_HKU3_9_Strain_NameHKU3_9_Segmentnull_HostUnknown.fa | B883 |
| GQ153546_OrganismBat_SARS_coronavirus_HKU3_11_Strain_NameHKU3_11_Segmentnull_HostUnknown.fa | B884 |
| GQ153540_OrganismBat_SARS_coronavirus_HKU3_5_Strain_NameHKU3_5_Segmentnull_HostUnknown.fa | B885 |
| KJ473814_OrganismBtRs_BetaCoV_HuB2013_Strain_NameBtRs_HuB2013_Segmentnull_HostBat.fa | B886 |
| DQ648857_OrganismBat_CoV_279_2005_Strain_NameBtCoV_279_2005_Segmentnull_HostUnknown.fa | B887 |
| DQ412043_OrganismBat_SARS_CoV_Rm1_2004_Strain_NameRm1_Segmentnull_HostBat.fa | B888 |
| JX993987_OrganismBat_coronavirus_Rp_Shaanxi2011_Strain_NameRp_Shaanxi2011_Segmentnull_HostBat.fa | B889 |
| KJ473811_OrganismBtRf_BetaCoV_JL2012_Strain_NameBtRf_JL2012_Segmentnull_HostBat.fa | B890 |
| KJ473812_OrganismBtRf_BetaCoV_HeB2013_Strain_NameBtRf_HeB2013_Segmentnull_HostBat.fa | B891 |
| KJ473813_OrganismBtRf_BetaCoV_SX2013_Strain_NameBtRf_SX2013_Segmentnull_HostBat.fa | B892 |
| DQ648856_OrganismBat_CoV_273_2005_Strain_NameBtCoV_273_2005_Segmentnull_HostUnknown.fa | B893 |
| DQ412042_OrganismBat_SARS_CoV_Rf1_2004_Strain_NameRf1_Segmentnull_HostBat.fa | B894 |
| KU973692_OrganismSARS_related_coronavirus_Strain_NameF46_Segmentnull_HostBat.fa | B895 |
| KF569996_OrganismRhinolophus_affinis_coronavirus_Strain_NameLYRa11_Segmentnull_HostBat.fa | B896 |
| JX993988_OrganismBat_coronavirus_Cp_Yunnan2011_Strain_NameCp_Yunnan2011_Segmentnull_HostBat.fa | B897 |
| DQ071615_OrganismBat_SARS_CoV_Rp3_2004_Strain_NameRp3_Segmentnull_HostBat.fa | B898 |
| KJ473815_OrganismBtRs_BetaCoV_GX2013_Strain_NameBtRs_GX2013_Segmentnull_HostBat.fa | B899 |
| AY427439_OrganismSARS_coronavirus_AS_Strain_NameAS_Segmentnull_HostHuman.fa | B900 |
| AY714217_OrganismSARS_Coronavirus_CDC#200301157_Strain_NameCDC#200301157_Segmentnull_HostHuman.fa | B901 |
| FJ882963_OrganismSARS_coronavirus_P2_Strain_NameP2_Segmentnull_HostHuman.fa | B902 |
| AY278491_OrganismSARS_coronavirus_HKU_39849_Strain_NameHKU_39849_Segmentnull_HostHuman.fa | B903 |
| CS079028_OrganismSARS_coronavirus_Strain_NameUNKNOWN_CS079028_Segmentnull_HostUnknown.fa | B904 |
| AY283796_OrganismSARS_coronavirus_Sin2679_Strain_NameSIN2679_Segmentnull_HostUnknown.fa | B905 |
| AY282752_OrganismSARS_coronavirus_CUHK_Su10_Strain_NameCUHK_Su10_Segmentnull_HostHuman.fa | B906 |
| AY357075_OrganismSARS_coronavirus_PUMC02_Strain_NamePUMC02_Segmentnull_HostUnknown.fa | B907 |
| AY485278_OrganismSARS_coronavirus_Sino3_11_Strain_NameSino3_11_Segmentnull_HostHuman.fa | B908 |
| AY502928_OrganismSARS_coronavirus_TW5_Strain_NameTW5_Segmentnull_HostHuman.fa | B909 |
| EU371559_OrganismSARS_coronavirus_ZJ02_Strain_NameZJ02_Segmentnull_HostUnknown.fa | B910 |
| EU371561_OrganismSARS_coronavirus_BJ182b_Strain_NameBJ182b_Segmentnull_HostUnknown.fa | B911 |
| EU371560_OrganismSARS_coronavirus_BJ182a_Strain_NameBJ182a_Segmentnull_HostUnknown.fa | B912 |
| EU371562_OrganismSARS_coronavirus_BJ182_4_Strain_NameBJ182_4_Segmentnull_HostUnknown.fa | B913 |
| EU371563_OrganismSARS_coronavirus_BJ182_8_Strain_NameBJ182_8_Segmentnull_HostUnknown.fa | B914 |
| EU371564_OrganismSARS_coronavirus_BJ182_12_Strain_NameBJ182_12_Segmentnull_HostUnknown.fa | B915 |
| AY297028_OrganismSARS_coronavirus_ZJ01_Strain_NameZJ01_Segmentnull_HostUnknown.fa | B916 |
| DQ182595_OrganismSARS_coronavirus_ZJ0301_Strain_NameZJ0301_Segmentnull_HostHuman.fa | B917 |
| AY394998_OrganismSARS_coronavirus_LC1_Strain_NameLC1_Segmentnull_HostUnknown.fa | B918 |
| AY357076_OrganismSARS_coronavirus_PUMC03_Strain_NamePUMC03_Segmentnull_HostUnknown.fa | B919 |
| AY485277_OrganismSARS_coronavirus_Sino1_11_Strain_NameSino1_11_Segmentnull_HostHuman.fa | B920 |
| AY350750_OrganismSARS_coronavirus_PUMC01_Strain_NamePUMC01_Segmentnull_HostUnknown.fa | B921 |
| AY348314_OrganismSARS_coronavirus_Taiwan_TC3_Strain_NameTC3_Segmentnull_HostUnknown.fa | B922 |
| AY338175_OrganismSARS_coronavirus_Taiwan_TC2_Strain_NameTC2_Segmentnull_HostUnknown.fa | B923 |
| AY313906_OrganismSARS_coronavirus_GD69_Strain_NameGD69_Segmentnull_HostHuman.fa | B924 |
| AY502924_OrganismSARS_coronavirus_TW11_Strain_NameTW11_Segmentnull_HostHuman.fa | B925 |
| AY345987_OrganismSARS_coronavirus_CUHK_AG02_Strain_NameCUHK_AG02_Segmentnull_HostHuman.fa | B926 |
| AY345986_OrganismSARS_coronavirus_CUHK_AG01_Strain_NameCUHK_AG01_Segmentnull_HostHuman.fa | B927 |
| AY338174_OrganismSARS_coronavirus_Taiwan_TC1_Strain_NameTC1_Segmentnull_HostUnknown.fa | B928 |
| AP006560_OrganismSARS_coronavirus_TWS_Strain_NameTWS_Segmentnull_HostHuman.fa | B929 |
| AY362699_OrganismSARS_coronavirus_TWC3_Strain_NameTWC3_Segmentnull_HostUnknown.fa | B930 |
| AY502932_OrganismSARS_coronavirus_TW9_Strain_NameTW9_Segmentnull_HostHuman.fa | B931 |
| AY502931_OrganismSARS_coronavirus_TW8_Strain_NameTW8_Segmentnull_HostHuman.fa | B932 |
| AY362698_OrganismSARS_coronavirus_TWC2_Strain_NameTWC2_Segmentnull_HostUnknown.fa | B933 |
| AP006557_OrganismSARS_coronavirus_TWH_Strain_NameTWH_Segmentnull_HostHuman.fa | B934 |
| AY502930_OrganismSARS_coronavirus_TW7_Strain_NameTW7_Segmentnull_HostHuman.fa | B935 |
| AP006561_OrganismSARS_coronavirus_TWY_Strain_NameTWY_Segmentnull_HostHuman.fa | B936 |
| AY502923_OrganismSARS_coronavirus_TW10_Strain_NameTW10_Segmentnull_HostHuman.fa | B937 |
| AP006559_OrganismSARS_coronavirus_TWK_Strain_NameTWK_Segmentnull_HostHuman.fa | B938 |
| AY345988_OrganismSARS_coronavirus_CUHK_AG03_Strain_NameCUHK_AG03_Segmentnull_HostHuman.fa | B939 |
| AP006558_OrganismSARS_coronavirus_TWJ_Strain_NameTWJ_Segmentnull_HostHuman.fa | B940 |
| AY502929_OrganismSARS_coronavirus_TW6_Strain_NameTW6_Segmentnull_HostHuman.fa | B941 |
| AY283794_OrganismSARS_coronavirus_Sin2500_Strain_NameSIN2500_Segmentnull_HostUnknown.fa | B942 |
| AY283797_OrganismSARS_coronavirus_Sin2748_Strain_NameSIN2748_Segmentnull_HostUnknown.fa | B943 |
| AY283798_OrganismSARS_coronavirus_Sin2774_Strain_NameSIN2774_Segmentnull_HostUnknown.fa | B944 |
| AY559092_OrganismSARS_coronavirus_SinP5_Strain_NameSinP5_Segmentnull_HostUnknown.fa | B945 |
| AY559088_OrganismSARS_coronavirus_SinP1_Strain_NameSinP1_Segmentnull_HostUnknown.fa | B946 |
| AY559089_OrganismSARS_coronavirus_SinP2_Strain_NameSinP2_Segmentnull_HostUnknown.fa | B947 |
| AY559091_OrganismSARS_coronavirus_SinP4_Strain_NameSinP4_Segmentnull_HostUnknown.fa | B948 |
| AY310120_OrganismSARS_coronavirus_FRA_Strain_NameFRA_Segmentnull_HostHuman.fa | B949 |
| AY291315_OrganismSARS_coronavirus_Frankfurt_1_Strain_NameFrankfurt_1_Segmentnull_HostHuman.fa | B950 |
| AB257344_OrganismSARS_coronavirus_Frankfurt_1_Strain_NameFrankfurt_1_Segmentnull_HostUnknown.fa | B951 |
| AY461660_OrganismSARS_coronavirus_SoD_Strain_NameSoD_Segmentnull_HostHuman.fa | B952 |
| AY559090_OrganismSARS_coronavirus_SinP3_Strain_NameSinP3_Segmentnull_HostUnknown.fa | B953 |
| AY283795_OrganismSARS_coronavirus_Sin2677_Strain_NameSIN2677_Segmentnull_HostUnknown.fa | B954 |
| MK062180_OrganismSARS_coronavirus_Urbani_Strain_NameUrbani_Segmentnull_HostHuman.fa | B955 |
| JF292904_OrganismSARS_coronavirus_MA15_ExoN1_Strain_NameMA15_ExoN1_mutant_Segmentnull_HostMouse.fa | B956 |
| HQ890539_OrganismSARS_coronavirus_MA15_ExoN1_Strain_NameMA15_ExoN1_mutant_Segmentnull_HostMouse.fa | B957 |
| HQ890538_OrganismSARS_coronavirus_MA15_ExoN1_Strain_NameMA15_ExoN1_mutant_Segmentnull_HostMouse.fa | B958 |
| HQ890540_OrganismSARS_coronavirus_MA15_ExoN1_Strain_NameMA15_ExoN1_mutant_Segmentnull_HostMouse.fa | B959 |
| HQ890531_OrganismSARS_coronavirus_MA15_ExoN1_Strain_NameMA15_ExoN1_mutant_Segmentnull_HostMouse.fa | B960 |
| HQ890529_OrganismSARS_coronavirus_MA15_ExoN1_Strain_NameMA15_ExoN1_mutant_Segmentnull_HostMouse.fa | B961 |
| HQ890534_OrganismSARS_coronavirus_MA15_ExoN1_Strain_NameMA15_ExoN1_mutant_Segmentnull_HostMouse.fa | B962 |
| HQ890536_OrganismSARS_coronavirus_MA15_ExoN1_Strain_NameMA15_ExoN1_mutant_Segmentnull_HostMouse.fa | B963 |
| HQ890527_OrganismSARS_coronavirus_MA15_ExoN1_Strain_NameMA15_ExoN1_mutant_Segmentnull_HostMouse.fa | B964 |
| HQ890526_OrganismSARS_coronavirus_MA15_ExoN1_Strain_NameMA15_ExoN1_mutant_Segmentnull_HostMouse.fa | B965 |
| HQ890528_OrganismSARS_coronavirus_MA15_ExoN1_Strain_NameMA15_ExoN1_mutant_Segmentnull_HostMouse.fa | B966 |
| JF292902_OrganismSARS_coronavirus_MA15_ExoN1_Strain_NameMA15_ExoN1_mutant_Segmentnull_HostMouse.fa | B967 |
| HQ890537_OrganismSARS_coronavirus_MA15_ExoN1_Strain_NameMA15_ExoN1_mutant_Segmentnull_HostMouse.fa | B968 |
| HQ890530_OrganismSARS_coronavirus_MA15_ExoN1_Strain_NameMA15_ExoN1_mutant_Segmentnull_HostMouse.fa | B969 |
| HQ890533_OrganismSARS_coronavirus_MA15_ExoN1_Strain_NameMA15_ExoN1_mutant_Segmentnull_HostMouse.fa | B970 |
| HQ890532_OrganismSARS_coronavirus_MA15_ExoN1_Strain_NameMA15_ExoN1_mutant_Segmentnull_HostMouse.fa | B971 |
| JF292905_OrganismSARS_coronavirus_MA15_ExoN1_Strain_NameMA15_ExoN1_mutant_Segmentnull_HostMouse.fa | B972 |
| JF292903_OrganismSARS_coronavirus_MA15_ExoN1_Strain_NameMA15_ExoN1_mutant_Segmentnull_HostMouse.fa | B973 |
| FJ882943_OrganismSARS_coronavirus_MA15_ExoN1_Strain_NameMA15_ExoN1_mutant_Segmentnull_HostUnknown.fa | B974 |
| JF292906_OrganismSARS_coronavirus_MA15_ExoN1_Strain_NameMA15_ExoN1_mutant_Segmentnull_HostMouse.fa | B975 |
| HQ890535_OrganismSARS_coronavirus_MA15_ExoN1_Strain_NameMA15_ExoN1_mutant_Segmentnull_HostMouse.fa | B976 |
| FJ882953_OrganismSARS_coronavirus_MA15_ExoN1_Strain_NameMA15_ExoN1_mutant_Segmentnull_HostUnknown.fa | B977 |
| FJ882962_OrganismSARS_coronavirus_MA15_ExoN1_Strain_NameMA15_ExoN1_mutant_Segmentnull_HostUnknown.fa | B978 |
| FJ882959_OrganismSARS_coronavirus_MA15_ExoN1_Strain_NameMA15_ExoN1_mutant_Segmentnull_HostUnknown.fa | B979 |
| FJ882951_OrganismSARS_coronavirus_MA15_ExoN1_Strain_NameMA15_ExoN1_mutant_Segmentnull_HostUnknown.fa | B980 |
| FJ882942_OrganismSARS_coronavirus_MA15_ExoN1_Strain_NameMA15_ExoN1_mutant_Segmentnull_HostUnknown.fa | B981 |
| FJ882957_OrganismSARS_coronavirus_MA15_Strain_NameMA15_Segmentnull_HostUnknown.fa | B982 |
| JF292922_OrganismSARS_coronavirus_ExoN1_Strain_NameExoN1_mutant_Segmentnull_HostUnknown.fa | B983 |
| JX162087_OrganismSARS_coronavirus_ExoN1_Strain_NameExoN1_mutant_Segmentnull_HostUnknown.fa | B984 |
| KF514407_OrganismSARS_coronavirus_ExoN1_Strain_NameSARS_VeroE6_lab_USA_ExoN1_c5.7P20_2010_Segmentnull_HostUnknown.fa | B985 |
| KF514410_OrganismSARS_coronavirus_ExoN1_Strain_NameSARS_VeroE6_lab_USA_ExoN1_c8P20_2009_Segmentnull_HostUnknown.fa | B986 |
| KF514406_OrganismSARS_coronavirus_ExoN1_Strain_NameSARS_VeroE6_lab_USA_ExoN1_c13P1_2009_Segmentnull_HostUnknown.fa | B987 |
| KF514417_OrganismSARS_coronavirus_ExoN1_Strain_NameSARS_VeroE6_lab_USA_ExoN1_c5.3P20_2010_Segmentnull_HostUnknown.fa | B988 |
| FJ882930_OrganismSARS_coronavirus_ExoN1_Strain_NameExoN1_mutant_Segmentnull_HostUnknown.fa | B989 |
| KF514411_OrganismSARS_coronavirus_ExoN1_Strain_NameSARS_VeroE6_lab_USA_ExoN1_c13P10_2009_Segmentnull_HostUnknown.fa | B990 |
| KF514389_OrganismSARS_coronavirus_ExoN1_Strain_NameSARS_VeroE6_lab_USA_ExoN1_c8P10_2009_Segmentnull_HostUnknown.fa | B991 |
| KF514401_OrganismSARS_coronavirus_ExoN1_Strain_NameSARS_VeroE6_lab_USA_ExoN1_c5.5P20_2010_Segmentnull_HostUnknown.fa | B992 |
| KF514390_OrganismSARS_coronavirus_ExoN1_Strain_NameSARS_VeroE6_lab_USA_ExoN1_c5.4P20_2010_Segmentnull_HostUnknown.fa | B993 |
| KF514416_OrganismSARS_coronavirus_ExoN1_Strain_NameSARS_VeroE6_lab_USA_ExoN1_c5.8P20_2010_Segmentnull_HostUnknown.fa | B994 |
| KF514414_OrganismSARS_coronavirus_ExoN1_Strain_NameSARS_VeroE6_lab_USA_ExoN1_c5P20_2009_Segmentnull_HostUnknown.fa | B995 |
| KF514402_OrganismSARS_coronavirus_ExoN1_Strain_NameSARS_VeroE6_lab_USA_ExoN1_c5.6P20_2010_Segmentnull_HostUnknown.fa | B996 |
| KF514405_OrganismSARS_coronavirus_ExoN1_Strain_NameSARS_VeroE6_lab_USA_ExoN1_c5.2P20_2010_Segmentnull_HostUnknown.fa | B997 |
| KF514412_OrganismSARS_coronavirus_ExoN1_Strain_NameSARS_VeroE6_lab_USA_ExoN1_c13P20_2009_Segmentnull_HostUnknown.fa | B998 |
| KF514391_OrganismSARS_coronavirus_ExoN1_Strain_NameSARS_VeroE6_lab_USA_ExoN1_c5.9P20_2010_Segmentnull_HostUnknown.fa | B999 |
| KF514393_OrganismSARS_coronavirus_ExoN1_Strain_NameSARS_VeroE6_lab_USA_ExoN1_c5.10P20_2010_Segmentnull_HostUnknown.fa | B1000 |
| KF514403_OrganismSARS_coronavirus_ExoN1_Strain_NameSARS_VeroE6_lab_USA_ExoN1_c5.1P20_2010_Segmentnull_HostUnknown.fa | B1001 |
| KF514395_OrganismSARS_coronavirus_ExoN1_Strain_NameSARS_VeroE6_lab_USA_ExoN1_c8P1_2009_Segmentnull_HostUnknown.fa | B1002 |
| KF514420_OrganismSARS_coronavirus_ExoN1_Strain_NameSARS_VeroE6_lab_USA_ExoN1_c5P10_2009_Segmentnull_HostUnknown.fa | B1003 |
| FJ882955_OrganismSARS_coronavirus_ExoN1_Strain_NameExoN1_mutant_Segmentnull_HostUnknown.fa | B1004 |
| FJ882960_OrganismSARS_coronavirus_ExoN1_Strain_NameExoN1_mutant_Segmentnull_HostUnknown.fa | B1005 |
| FJ882941_OrganismSARS_coronavirus_ExoN1_Strain_NameExoN1_mutant_Segmentnull_HostUnknown.fa | B1006 |
| FJ882940_OrganismSARS_coronavirus_ExoN1_Strain_NameExoN1_mutant_Segmentnull_HostUnknown.fa | B1007 |
| FJ882929_OrganismSARS_coronavirus_ExoN1_Strain_NameExoN1_mutant_Segmentnull_HostUnknown.fa | B1008 |
| FJ882956_OrganismSARS_coronavirus_ExoN1_Strain_NameExoN1_mutant_Segmentnull_HostUnknown.fa | B1009 |
| FJ882950_OrganismSARS_coronavirus_ExoN1_Strain_NameExoN1_mutant_Segmentnull_HostUnknown.fa | B1010 |
| FJ882954_OrganismSARS_coronavirus_ExoN1_Strain_NameExoN1_mutant_Segmentnull_HostUnknown.fa | B1011 |
| FJ882944_OrganismSARS_coronavirus_ExoN1_Strain_NameExoN1_mutant_Segmentnull_HostUnknown.fa | B1012 |
| FJ882931_OrganismSARS_coronavirus_ExoN1_Strain_NameExoN1_mutant_Segmentnull_HostUnknown.fa | B1013 |
| FJ882926_OrganismSARS_coronavirus_ExoN1_Strain_NameExoN1_mutant_Segmentnull_HostUnknown.fa | B1014 |
| FJ882928_OrganismSARS_coronavirus_ExoN1_Strain_NameExoN1_mutant_Segmentnull_HostUnknown.fa | B1015 |
| KF514392_OrganismSARS_coronavirus_wtic_MB_Strain_NameSARS_VeroE6_lab_USA_WTic_c1.4P20_2010_Segmentnull_HostUnknown.fa | B1016 |
| KF514404_OrganismSARS_coronavirus_wtic_MB_Strain_NameSARS_VeroE6_lab_USA_WTic_c1.9P20_2010_Segmentnull_HostUnknown.fa | B1017 |
| KF514398_OrganismSARS_coronavirus_wtic_MB_Strain_NameSARS_VeroE6_lab_USA_WTic_c1.10P20_2010_Segmentnull_HostUnknown.fa | B1018 |
| KF514419_OrganismSARS_coronavirus_wtic_MB_Strain_NameSARS_VeroE6_lab_USA_WTic_c1P10_2009_Segmentnull_HostUnknown.fa | B1019 |
| KF514409_OrganismSARS_coronavirus_wtic_MB_Strain_NameSARS_VeroE6_lab_USA_WTic_c2P20_2009_Segmentnull_HostUnknown.fa | B1020 |
| KF514388_OrganismSARS_coronavirus_wtic_MB_Strain_NameSARS_VeroE6_lab_USA_WTic_c1.5P20_2010_Segmentnull_HostUnknown.fa | B1021 |
| KF514394_OrganismSARS_coronavirus_wtic_MB_Strain_NameSARS_VeroE6_lab_USA_WTic_c1P20_2009_Segmentnull_HostUnknown.fa | B1022 |
| KF514422_OrganismSARS_coronavirus_wtic_MB_Strain_NameSARS_VeroE6_lab_USA_WTic_c1.3P20_2010_Segmentnull_HostUnknown.fa | B1023 |
| KF514413_OrganismSARS_coronavirus_wtic_MB_Strain_NameSARS_VeroE6_lab_USA_WTic_c1.6P20_2010_Segmentnull_HostUnknown.fa | B1024 |
| KF514396_OrganismSARS_coronavirus_wtic_MB_Strain_NameSARS_VeroE6_lab_USA_WTic_c3P10_2009_Segmentnull_HostUnknown.fa | B1025 |
| KF514400_OrganismSARS_coronavirus_wtic_MB_Strain_NameSARS_VeroE6_lab_USA_WTic_c1.8P20_2010_Segmentnull_HostUnknown.fa | B1026 |
| KF514397_OrganismSARS_coronavirus_wtic_MB_Strain_NameSARS_VeroE6_lab_USA_WTic_c2P10_2009_Segmentnull_HostUnknown.fa | B1027 |
| KF514421_OrganismSARS_coronavirus_wtic_MB_Strain_NameSARS_VeroE6_lab_USA_WTic_c1.2P20_2010_Segmentnull_HostUnknown.fa | B1028 |
| KF514415_OrganismSARS_coronavirus_wtic_MB_Strain_NameSARS_VeroE6_lab_USA_WTic_c1.7P20_2010_Segmentnull_HostUnknown.fa | B1029 |
| KF514399_OrganismSARS_coronavirus_wtic_MB_Strain_NameSARS_VeroE6_lab_USA_WTic_c1.1P20_2010_Segmentnull_HostUnknown.fa | B1030 |
| KF514418_OrganismSARS_coronavirus_wtic_MB_Strain_NameSARS_VeroE6_lab_USA_WTic_c3P1_2009_Segmentnull_HostUnknown.fa | B1031 |
| KF514408_OrganismSARS_coronavirus_wtic_MB_Strain_NameSARS_VeroE6_lab_USA_WTic_c2P1_2009_Segmentnull_HostUnknown.fa | B1032 |
| KF514423_OrganismSARS_coronavirus_wtic_MB_Strain_NameSARS_VeroE6_lab_USA_WTic_c3P20_2009_Segmentnull_HostUnknown.fa | B1033 |
| FJ882938_OrganismSARS_coronavirus_wtic_MB_Strain_Namewtic_MB_Segmentnull_HostUnknown.fa | B1034 |
| FJ882927_OrganismSARS_coronavirus_wtic_MB_Strain_Namewtic_MB_Segmentnull_HostUnknown.fa | B1035 |
| FJ882937_OrganismSARS_coronavirus_wtic_MB_Strain_Namewtic_MB_Segmentnull_HostUnknown.fa | B1036 |
| FJ882939_OrganismSARS_coronavirus_wtic_MB_Strain_Namewtic_MB_Segmentnull_HostUnknown.fa | B1037 |
| FJ882933_OrganismSARS_coronavirus_wtic_MB_Strain_Namewtic_MB_Segmentnull_HostUnknown.fa | B1038 |
| FJ882934_OrganismSARS_coronavirus_wtic_MB_Strain_Namewtic_MB_Segmentnull_HostUnknown.fa | B1039 |
| FJ882932_OrganismSARS_coronavirus_wtic_MB_Strain_Namewtic_MB_Segmentnull_HostUnknown.fa | B1040 |
| FJ882946_OrganismSARS_coronavirus_wtic_MB_Strain_Namewtic_MB_Segmentnull_HostUnknown.fa | B1041 |
| FJ882935_OrganismSARS_coronavirus_wtic_MB_Strain_Namewtic_MB_Segmentnull_HostUnknown.fa | B1042 |
| JF292921_OrganismSARS_coronavirus_wtic_MB_Strain_NameWTic_Segmentnull_HostUnknown.fa | B1043 |
| FJ882936_OrganismSARS_coronavirus_wtic_MB_Strain_Namewtic_MB_Segmentnull_HostUnknown.fa | B1044 |
| FJ882947_OrganismSARS_coronavirus_wtic_MB_Strain_Namewtic_MB_Segmentnull_HostUnknown.fa | B1045 |
| FJ882949_OrganismSARS_coronavirus_wtic_MB_Strain_Namewtic_MB_Segmentnull_HostUnknown.fa | B1046 |
| FJ882958_OrganismSARS_coronavirus_MA15_Strain_NameMA15_Segmentnull_HostUnknown.fa | B1047 |
| FJ882948_OrganismSARS_coronavirus_MA15_Strain_NameMA15_Segmentnull_HostUnknown.fa | B1048 |
| FJ882961_OrganismSARS_coronavirus_MA15_Strain_NameMA15_Segmentnull_HostUnknown.fa | B1049 |
| FJ882952_OrganismSARS_coronavirus_MA15_Strain_NameMA15_Segmentnull_HostUnknown.fa | B1050 |
| FJ882945_OrganismSARS_coronavirus_MA15_Strain_NameMA15_Segmentnull_HostUnknown.fa | B1051 |
| JF292909_OrganismSARS_coronavirus_MA15_Strain_NameMA15_Segmentnull_HostMouse.fa | B1052 |
| JF292914_OrganismSARS_coronavirus_MA15_Strain_NameMA15_Segmentnull_HostMouse.fa | B1053 |
| JF292913_OrganismSARS_coronavirus_MA15_Strain_NameMA15_Segmentnull_HostMouse.fa | B1054 |
| JF292910_OrganismSARS_coronavirus_MA15_Strain_NameMA15_Segmentnull_HostMouse.fa | B1055 |
| JF292920_OrganismSARS_coronavirus_MA15_Strain_NameMA15_Segmentnull_HostMouse.fa | B1056 |
| JF292908_OrganismSARS_coronavirus_MA15_Strain_NameMA15_Segmentnull_HostMouse.fa | B1057 |
| JF292907_OrganismSARS_coronavirus_MA15_Strain_NameMA15_Segmentnull_HostMouse.fa | B1058 |
| JF292917_OrganismSARS_coronavirus_MA15_Strain_NameMA15_Segmentnull_HostMouse.fa | B1059 |
| JF292916_OrganismSARS_coronavirus_MA15_Strain_NameMA15_Segmentnull_HostMouse.fa | B1060 |
| JF292912_OrganismSARS_coronavirus_MA15_Strain_NameMA15_Segmentnull_HostMouse.fa | B1061 |
| JF292911_OrganismSARS_coronavirus_MA15_Strain_NameMA15_Segmentnull_HostMouse.fa | B1062 |
| HQ890544_OrganismSARS_coronavirus_MA15_Strain_NameMA15_Segmentnull_HostMouse.fa | B1063 |
| JF292919_OrganismSARS_coronavirus_MA15_Strain_NameMA15_Segmentnull_HostMouse.fa | B1064 |
| JF292918_OrganismSARS_coronavirus_MA15_Strain_NameMA15_Segmentnull_HostMouse.fa | B1065 |
| HQ890545_OrganismSARS_coronavirus_MA15_Strain_NameMA15_Segmentnull_HostMouse.fa | B1066 |
| HQ890546_OrganismSARS_coronavirus_MA15_Strain_NameMA15_Segmentnull_HostMouse.fa | B1067 |
| HQ890542_OrganismSARS_coronavirus_MA15_Strain_NameMA15_Segmentnull_HostMouse.fa | B1068 |
| HQ890543_OrganismSARS_coronavirus_MA15_Strain_NameMA15_Segmentnull_HostMouse.fa | B1069 |
| HQ890541_OrganismSARS_coronavirus_MA15_Strain_NameMA15_Segmentnull_HostMouse.fa | B1070 |
| JF292915_OrganismSARS_coronavirus_MA15_Strain_NameMA15_Segmentnull_HostMouse.fa | B1071 |
| MK062184_OrganismSARS_coronavirus_Urbani_Strain_NameUrbani_Segmentnull_HostHuman.fa | B1072 |
| MK062183_OrganismSARS_coronavirus_Urbani_Strain_NameUrbani_Segmentnull_HostHuman.fa | B1073 |
| MK062181_OrganismSARS_coronavirus_Urbani_Strain_NameUrbani_Segmentnull_HostHuman.fa | B1074 |
| MK062182_OrganismSARS_coronavirus_Urbani_Strain_NameUrbani_Segmentnull_HostHuman.fa | B1075 |
| MK062179_OrganismSARS_coronavirus_Urbani_Strain_NameUrbani_Segmentnull_HostHuman.fa | B1076 |
| DQ497008_OrganismSARS_coronavirus_Strain_NameMA_15_Segmentnull_HostMouse.fa | B1077 |
| DJ066921_OrganismSARS_coronavirus_Urbani_Strain_NameUNKNOWN_DJ066921_Segmentnull_HostUnknown.fa | B1078 |
| AY278741_OrganismSARS_coronavirus_Urbani_Strain_NameUrbani_Segmentnull_HostUnknown.fa | B1079 |
| CS079027_OrganismSARS_coronavirus_Strain_NameUNKNOWN_CS079027_Segmentnull_HostUnknown.fa | B1080 |
| CS460762_OrganismSARS_coronavirus_Strain_NameSequence_11_from_Patent_EP1736539_Segmentnull_HostUnknown.fa | B1081 |
| CS480537_OrganismSARS_coronavirus_Strain_NameSequence_11_from_Patent_WO2006136448_Segmentnull_HostUnknown.fa | B1082 |
| JN854286_OrganismSARS_coronavirus_HKU_39849_Strain_NamerecSARS_CoV_HKU_39849_Segmentnull_HostUnknown.fa | B1083 |
| GU553363_OrganismSARS_coronavirus_HKU_39849_Strain_NameHKU_39849_Segmentnull_HostHuman.fa | B1084 |
| GU553364_OrganismSARS_coronavirus_HKU_39849_Strain_NameHKU_39849_Segmentnull_HostHuman.fa | B1085 |
| GU553365_OrganismSARS_coronavirus_HKU_39849_Strain_NameHKU_39849_Segmentnull_HostMonkey.fa | B1086 |
| AY321118_OrganismSARS_coronavirus_TWC_Strain_NameTWC_Segmentnull_HostUnknown.fa | B1087 |
| AY351680_OrganismSARS_coronavirus_ZMY_1_Strain_NameZMY_1_Segmentnull_HostUnknown.fa | B1088 |
| AY394850_OrganismSARS_coronavirus_WHU_Strain_NameWHU_Segmentnull_HostHuman.fa | B1089 |
| AY323977_OrganismSARS_coronavirus_HSR_1_Strain_NameHSR_1_Segmentnull_HostHuman.fa | B1090 |
| JQ316196_OrganismSARS_coronavirus_HKU_39849_Strain_NameHKU_39849_Segmentnull_HostUnknown.fa | B1091 |
| JX163928_OrganismSARS_coronavirus_Strain_NameTor2_FP1_10895_Segmentnull_HostUnknown.fa | B1092 |
| AY394991_OrganismSARS_coronavirus_HZS2_Fc_Strain_NameHZS2_Fc_Segmentnull_HostHuman.fa | B1093 |
| AY278487_OrganismSARS_coronavirus_BJ02_Strain_NameBJ02_Segmentnull_HostHuman.fa | B1094 |
| AY278488_OrganismSARS_coronavirus_BJ01_Strain_NameBJ01_Segmentnull_HostHuman.fa | B1095 |
| AY278490_OrganismSARS_coronavirus_BJ03_Strain_NameBJ03_Segmentnull_HostUnknown.fa | B1096 |
| AY595412_OrganismSARS_coronavirus_LLJ_2004_Strain_NameLLJ_2004_Segmentnull_HostUnknown.fa | B1097 |
| AY394992_OrganismSARS_coronavirus_HZS2_C_Strain_NameHZS2_C_Segmentnull_HostUnknown.fa | B1098 |
| CS079029_OrganismSARS_coronavirus_Strain_NameUNKNOWN_CS079029_Segmentnull_HostUnknown.fa | B1099 |
| AY278554_OrganismSARS_coronavirus_CUHK_W1_Strain_NameCUHK_W1_Segmentnull_HostHuman.fa | B1100 |
| AY394983_OrganismSARS_coronavirus_HSZ2_A_Strain_NameHSZ2_A_Segmentnull_HostUnknown.fa | B1101 |
| AY394990_OrganismSARS_coronavirus_HZS2_E_Strain_NameHZS2_E_Segmentnull_HostUnknown.fa | B1102 |
| AY394989_OrganismSARS_coronavirus_HZS2_D_Strain_NameHZS2_D_Segmentnull_HostUnknown.fa | B1103 |
| AY394993_OrganismSARS_coronavirus_HGZ8L2_Strain_NameHGZ8L2_Segmentnull_HostHuman.fa | B1104 |
| AY304488_OrganismCivet_SARS_CoV_SZ16_2003_Strain_NameSZ16_Segmentnull_HostCivet.fa | B1105 |
| AY304486_OrganismCivet_SARS_CoV_SZ3_2003_Strain_NameSZ3_Segmentnull_HostCivet.fa | B1106 |
| AY545917_OrganismSARS_coronavirus_Strain_NameHC_GZ_81_03_Segmentnull_HostCivet.fa | B1107 |
| AY613950_OrganismSARS_coronavirus_PC4_227_Strain_NamePC4_227_Segmentnull_HostCivet.fa | B1108 |
| AY545916_OrganismSARS_coronavirus_Strain_NameHC_SZ_266_03_Segmentnull_HostCivet.fa | B1109 |
| AY545915_OrganismSARS_coronavirus_Strain_NameHC_SZ_DM1_03_Segmentnull_HostCivet.fa | B1110 |
| AY613949_OrganismSARS_coronavirus_PC4_136_Strain_NamePC4_136_Segmentnull_HostCivet.fa | B1111 |
| AY613948_OrganismSARS_coronavirus_PC4_13_Strain_NamePC4_13_Segmentnull_HostCivet.fa | B1112 |
| AY613947_OrganismSARS_coronavirus_GZ0402_Strain_NameGZ0402_Segmentnull_HostHuman.fa | B1113 |
| AY568539_OrganismSARS_coronavirus_GZ0401_Strain_NameGZ0401_Segmentnull_HostHuman.fa | B1114 |
| AY686864_OrganismSARS_coronavirus_B039_Strain_NameB039_Segmentnull_HostCivet.fa | B1115 |
| AY515512_OrganismSARS_coronavirus_HC_SZ_61_03_Strain_NameHC_SZ_61_03_Segmentnull_HostCivet.fa | B1116 |
| AY545919_OrganismSARS_coronavirus_Strain_NameCFB_SZ_94_03_Segmentnull_HostBadger.fa | B1117 |
| FJ959407_OrganismSARS_coronavirus_Strain_NameA001_Segmentnull_HostCivet.fa | B1118 |
| AY545918_OrganismSARS_coronavirus_Strain_NameHC_GZ_32_03_Segmentnull_HostCivet.fa | B1119 |
| AY572038_OrganismSARS_coronavirus_civet020_Strain_Namecivet020_Segmentnull_HostCivet.fa | B1120 |
| AY572034_OrganismCivet_SARS_CoV_007_2004_Strain_Namecivet007_Segmentnull_HostCivet.fa | B1121 |
| AY686863_OrganismSARS_coronavirus_A022_Strain_NameA022_Segmentnull_HostCivet.fa | B1122 |
| AY545914_OrganismSARS_coronavirus_Strain_NameHC_SZ_79_03_Segmentnull_HostCivet.fa | B1123 |
| AY572035_OrganismSARS_coronavirus_civet010_Strain_Namecivet010_Segmentnull_HostCivet.fa | B1124 |
| AY278489_OrganismSARS_coronavirus_GD01_Strain_NameGD01_Segmentnull_HostHuman.fa | B1125 |
| AY390556_OrganismSARS_coronavirus_GZ02_Strain_NameGZ02_Segmentnull_HostHuman.fa | B1126 |
| AY394996_OrganismSARS_coronavirus_ZS_B_Strain_NameZS_B_Segmentnull_HostUnknown.fa | B1127 |
| AY394997_OrganismSARS_coronavirus_ZS_A_Strain_NameZS_A_Segmentnull_HostUnknown.fa | B1128 |
| AY395003_OrganismSARS_coronavirus_ZS_C_Strain_NameZS_C_Segmentnull_HostHuman.fa | B1129 |
| AY394995_OrganismSARS_coronavirus_HSZ_Cc_Strain_NameHSZ_Cc_Segmentnull_HostHuman.fa | B1130 |
| AY394986_OrganismSARS_coronavirus_HSZ_Cb_Strain_NameHSZ_Cb_Segmentnull_HostUnknown.fa | B1131 |
| AY394985_OrganismSARS_coronavirus_HSZ_Bb_Strain_NameHSZ_Bb_Segmentnull_HostUnknown.fa | B1132 |
| AY394994_OrganismSARS_coronavirus_HSZ_Bc_Strain_NameHSZ_Bc_Segmentnull_HostUnknown.fa | B1133 |
| AY304495_OrganismSARS_coronavirus_GZ50_Strain_NameGZ50_Segmentnull_HostHuman.fa | B1134 |
| DQ640652_OrganismSARS_coronavirus_GDH_BJH01_Strain_NameGDH_BJH01_Segmentnull_HostHuman.fa | B1135 |
| AY864806_OrganismSARS_coronavirus_BJ202_Strain_NameBJ202_Segmentnull_HostUnknown.fa | B1136 |
| AY864805_OrganismSARS_coronavirus_BJ162_Strain_NameBJ162_Segmentnull_HostUnknown.fa | B1137 |
| AY772062_OrganismSARS_coronavirus_WH20_Strain_NameWH20_Segmentnull_HostUnknown.fa | B1138 |
| AY508724_OrganismSARS_coronavirus_NS_1_Strain_NameNS_1_Segmentnull_HostUnknown.fa | B1139 |
| AY279354_OrganismSARS_coronavirus_BJ04_Strain_NameBJ04_Segmentnull_HostHuman.fa | B1140 |
| AY654624_OrganismSARS_coronavirus_TJF_Strain_NameTJF_Segmentnull_HostSwine.fa | B1141 |
| AY394987_OrganismSARS_coronavirus_HZS2_Fb_Strain_NameHZS2_Fb_Segmentnull_HostUnknown.fa | B1142 |
| JX163923_OrganismSARS_coronavirus_Strain_NameTor2_FP1_10912_Segmentnull_HostUnknown.fa | B1143 |
| JX163925_OrganismSARS_coronavirus_Strain_NameTor2_FP1_10895_Segmentnull_HostUnknown.fa | B1144 |
| AY463059_OrganismSARS_coronavirus_ShanghaiQXC1_Strain_NameShanghaiQXC1_Segmentnull_HostUnknown.fa | B1145 |
| AY463060_OrganismSARS_coronavirus_ShanghaiQXC2_Strain_NameShanghaiQXC2_Segmentnull_HostUnknown.fa | B1146 |
| AY274119_OrganismSevere_acute_respiratory_syndrome_related_coronavirus_Strain_NameTor2_Segmentnull_HostHuman.fa | B1147 |
| NC_004718_OrganismSARS_coronavirus_Strain_NameTor2_Segmentnull_HostUnknown.fa | B1148 |
| DJ045279_OrganismSARS_coronavirus_Strain_NameUNKNOWN_DJ045279_Segmentnull_HostUnknown.fa | B1149 |
| CS079026_OrganismSARS_coronavirus_Strain_NameSequence_14_from_Patent_WO2005035556_Segmentnull_HostUnknown.fa | B1150 |
| CQ918598_OrganismSARS_coronavirus_Strain_NameSequence_15_from_Patent_WO2004096842_Segmentnull_HostUnknown.fa | B1151 |
| CS254197_OrganismSARS_coronavirus_Strain_NameSequence_67_from_Patent_WO2004011647_Segmentnull_HostUnknown.fa | B1152 |
| DL476508_OrganismSARS_coronavirus_Strain_NameUNKNOWN_DL476508_Segmentnull_HostUnknown.fa | B1153 |
| CS569493_OrganismSARS_coronavirus_Strain_NameSequence_1_from_Patent_WO2006039656_Segmentnull_HostUnknown.fa | B1154 |
| HW375992_OrganismSARS_coronavirus_Strain_NameUNKNOWN_HW375992_Segmentnull_HostUnknown.fa | B1155 |
| HW269828_OrganismSARS_coronavirus_Strain_NameUNKNOWN_HW269828_Segmentnull_HostUnknown.fa | B1156 |
| CQ918584_OrganismSARS_coronavirus_Strain_NameSequence_1_from_Patent_WO2004096842_Segmentnull_HostUnknown.fa | B1157 |
| CQ918585_OrganismSARS_coronavirus_Strain_NameSequence_2_from_Patent_WO2004096842_Segmentnull_HostUnknown.fa | B1158 |
| JX163927_OrganismSARS_coronavirus_Strain_NameTor2_FP1_10851_Segmentnull_HostUnknown.fa | B1159 |
| JX163926_OrganismSARS_coronavirus_Strain_NameTor2_FP1_10912_Segmentnull_HostUnknown.fa | B1160 |
| JX163924_OrganismSARS_coronavirus_Strain_NameTor2_FP1_10851_Segmentnull_HostUnknown.fa | B1161 |
| AY394979_OrganismSARS_coronavirus_GZ_C_Strain_NameGZ_C_Segmentnull_HostUnknown.fa | B1162 |
| DQ898174_OrganismSARS_coronavirus_Strain_NameCV7_Segmentnull_HostUnknown.fa | B1163 |
| AY394978_OrganismSARS_coronavirus_GZ_B_Strain_NameGZ_B_Segmentnull_HostUnknown.fa | B1164 |
| DJ059765_OrganismSARS_coronavirus_Strain_NameUNKNOWN_DJ059765_Segmentnull_HostUnknown.fa | B1165 |
| DL008527_OrganismSARS_coronavirus_Strain_NameUNKNOWN_DL008527_Segmentnull_HostUnknown.fa | B1166 |
| AY502925_OrganismSARS_coronavirus_TW2_Strain_NameTW2_Segmentnull_HostHuman.fa | B1167 |
| AY291451_OrganismSARS_coronavirus_TW1_Strain_NameTW1_Segmentnull_HostHuman.fa | B1168 |
| AY502926_OrganismSARS_coronavirus_TW3_Strain_NameTW3_Segmentnull_HostHuman.fa | B1169 |
| AY502927_OrganismSARS_coronavirus_TW4_Strain_NameTW4_Segmentnull_HostHuman.fa | B1170 |
| AY559081_OrganismSARS_coronavirus_Sin842_Strain_NameSin842_Segmentnull_HostHuman.fa | B1171 |
| AY559097_OrganismSARS_coronavirus_Sin3408L_Strain_NameSin3408L_Segmentnull_HostUnknown.fa | B1172 |
| AY559083_OrganismSARS_coronavirus_Sin3408_Strain_NameSin3408_Segmentnull_HostUnknown.fa | B1173 |
| AY559087_OrganismSARS_coronavirus_Sin3725V_Strain_NameSin3725V_Segmentnull_HostUnknown.fa | B1174 |
| AY559082_OrganismSARS_coronavirus_Sin852_Strain_NameSin852_Segmentnull_HostHuman.fa | B1175 |
| AY559084_OrganismSARS_coronavirus_Sin3765V_Strain_NameSin3765V_Segmentnull_HostUnknown.fa | B1176 |
| AY559095_OrganismSARS_coronavirus_Sin847_Strain_NameSin847_Segmentnull_HostUnknown.fa | B1177 |
| AY559085_OrganismSARS_coronavirus_Sin848_Strain_NameSin848_Segmentnull_HostHuman.fa | B1178 |
| AY559093_OrganismSARS_coronavirus_Sin845_Strain_NameSin845_Segmentnull_HostUnknown.fa | B1179 |
| AY559086_OrganismSARS_coronavirus_Sin849_Strain_NameSin849_Segmentnull_HostHuman.fa | B1180 |
| AY559096_OrganismSARS_coronavirus_Sin850_Strain_NameSin850_Segmentnull_HostHuman.fa | B1181 |
| AY559094_OrganismSARS_coronavirus_Sin846_Strain_NameSin846_Segmentnull_HostHuman.fa | B1182 |
| AY394999_OrganismSARS_coronavirus_LC2_Strain_NameLC2_Segmentnull_HostUnknown.fa | B1183 |
| AY395000_OrganismSARS_coronavirus_LC3_Strain_NameLC3_Segmentnull_HostUnknown.fa | B1184 |
| AY395001_OrganismSARS_coronavirus_LC4_Strain_NameLC4_Segmentnull_HostUnknown.fa | B1185 |
| AY395002_OrganismSARS_coronavirus_LC5_Strain_NameLC5_Segmentnull_HostHuman.fa | B1186 |
| KY417146_OrganismBat_SARS_like_coronavirus_Strain_NameRs4231_Segmentnull_HostBat.fa | B1187 |
| KT444582_OrganismSARS_like_coronavirus_WIV16_Strain_NameWIV16_Segmentnull_HostBat.fa | B1188 |
| KY417150_OrganismBat_SARS_like_coronavirus_Strain_NameRs4874_Segmentnull_HostBat.fa | B1189 |
| MK211376_OrganismCoronavirus_BtRs_BetaCoV_YN2018B_Strain_NameBtRs_BetaCoV_YN2018B_Segmentnull_HostBat.fa | B1190 |
| KY417151_OrganismBat_SARS_like_coronavirus_Strain_NameRs7327_Segmentnull_HostBat.fa | B1191 |
| KY417152_OrganismBat_SARS_like_coronavirus_Strain_NameRs9401_Segmentnull_HostBat.fa | B1192 |
| KF367457_OrganismBat_SARS_like_coronavirus_WIV1_Strain_NameWIV1_Segmentnull_HostBat.fa | B1193 |
| KC881006_OrganismBat_SARS_like_coronavirus_Rs3367_Strain_NameRs3367_Segmentnull_HostBat.fa | B1194 |
| KC881005_OrganismBat_SARS_like_coronavirus_RsSHC014_Strain_NameRsSHC014_Segmentnull_HostBat.fa | B1195 |
| KY417144_OrganismBat_SARS_like_coronavirus_Strain_NameRs4084_Segmentnull_HostBat.fa | B1196 |
| KY417147_OrganismBat_SARS_like_coronavirus_Strain_NameRs4237_Segmentnull_HostBat.fa | B1197 |
| KY417148_OrganismBat_SARS_like_coronavirus_Strain_NameRs4247_Segmentnull_HostBat.fa | B1198 |
| FJ588686_OrganismSARS_coronavirus_Rs_672_2006_Strain_NameRs672_Segmentnull_HostBat.fa | B1199 |
| MK211378_OrganismCoronavirus_BtRs_BetaCoV_YN2018D_Strain_NameBtRs_BetaCoV_YN2018D_Segmentnull_HostBat.fa | B1200 |
| KY417149_OrganismBat_SARS_like_coronavirus_Strain_NameRs4255_Segmentnull_HostBat.fa | B1201 |
| KY417143_OrganismBat_SARS_like_coronavirus_Strain_NameRs4081_Segmentnull_HostBat.fa | B1202 |
| MK211375_OrganismCoronavirus_BtRs_BetaCoV_YN2018A_Strain_NameBtRs_BetaCoV_YN2018A_Segmentnull_HostBat.fa | B1203 |
| MK211377_OrganismCoronavirus_BtRs_BetaCoV_YN2018C_Strain_NameBtRs_BetaCoV_YN2018C_Segmentnull_HostBat.fa | B1204 |
| KY417142_OrganismBat_SARS_like_coronavirus_Strain_NameAs6526_Segmentnull_HostBat.fa | B1205 |
| KY417145_OrganismBat_SARS_like_coronavirus_Strain_NameRf4092_Segmentnull_HostBat.fa | B1206 |
| KJ473816_OrganismBtRs_BetaCoV_YN2013_Strain_NameBtRs_YN2013_Segmentnull_HostBat.fa | B1207 |
| KP886808_OrganismBat_SARS_like_coronavirus_YNLF_31C_Strain_NameYNLF_31C_Segmentnull_HostBat.fa | B1208 |
| KP886809_OrganismBat_SARS_like_coronavirus_YNLF_34C_Strain_NameYNLF_34C_Segmentnull_HostBat.fa | B1209 |
| MK211374_OrganismCoronavirus_BtRl_BetaCoV_SC2018_Strain_NameBtRl_BetaCoV_SC2018_Segmentnull_HostBat.fa | B1210 |
| HI553383_OrganismSARS_coronavirus_Strain_NameSequence_41_from_Patent_EP2139515_Segmentnull_HostUnknown.fa | B1211 |
| MG596802_OrganismMiddle_East_respiratory_syndrome_related_coronavirus_Strain_NameBat_CoV_H.savii_Italy_206645_40_2011_Segmentnull_HostBat.fa | B1212 |
| MG596803_OrganismMiddle_East_respiratory_syndrome_related_coronavirus_Strain_NameBat_CoV_P.khulii_Italy_206645_63_2011_Segmentnull_HostBat.fa | B1213 |
| MG021452_OrganismMiddle_East_respiratory_syndrome_related_coronavirus_Strain_NameNL140422_Segmentnull_HostUnknown.fa | B1214 |
| MG021451_OrganismMiddle_East_respiratory_syndrome_related_coronavirus_Strain_NameNL13845_Segmentnull_HostUnknown.fa | B1215 |
| KJ473821_OrganismBtVs_BetaCoV_SC2013_Strain_NameUNKNOWN_KJ473821_Segmentnull_HostBat.fa | B1216 |
| MG987420_OrganismMiddle_East_respiratory_syndrome_related_coronavirus_Strain_NameNL13892_Segmentnull_HostUnknown.fa | B1217 |
| MG987421_OrganismMiddle_East_respiratory_syndrome_related_coronavirus_Strain_NameNL140455_Segmentnull_HostUnknown.fa | B1218 |
| KC869678_OrganismCoronavirus_Neoromicia_PML_PHE1_RSA_2011_Strain_NameNeoromicia_PML_PHE1_RSA_2011_Segmentnull_HostBat.fa | B1219 |
| MF593268_OrganismMiddle_East_respiratory_syndrome_related_coronavirus_Strain_NameNeoromicia_5038_Segmentnull_HostBat.fa | B1220 |
| KX108943_OrganismMiddle_East_respiratory_syndrome_coronavirus_Strain_NameD998_15_Segmentnull_HostCamel.fa | B1221 |
| KJ477103_OrganismMiddle_East_respiratory_syndrome_related_coronavirus_Strain_NameNRCE_HKU270_Segmentnull_HostCamel.fa | B1222 |
| MK967708_OrganismMiddle_East_respiratory_syndrome_related_coronavirus_Strain_NameMerscov_Egypt_Camel_AHRI_FAO_1_2018_Segmentnull_HostCamel.fa | B1223 |
| MG923473_OrganismMiddle_East_respiratory_syndrome_related_coronavirus_Strain_NameMERS_CoV_camel_Burkina_Faso_CIRAD_HKU697_2015_Segmentnull_HostCamel.fa | B1224 |
| MG923472_OrganismMiddle_East_respiratory_syndrome_related_coronavirus_Strain_NameMERS_CoV_camel_Nigeria_NS004_2015_Segmentnull_HostCamel.fa | B1225 |
| MG923475_OrganismMiddle_East_respiratory_syndrome_related_coronavirus_Strain_NameMERS_CoV_camel_Nigeria_NV1657_2016_Segmentnull_HostCamel.fa | B1226 |
| MG923476_OrganismMiddle_East_respiratory_syndrome_related_coronavirus_Strain_NameMERS_CoV_camel_Nigeria_NV1989_2016_Segmentnull_HostCamel.fa | B1227 |
| MG923481_OrganismMiddle_East_respiratory_syndrome_related_coronavirus_Strain_NameMERS_CoV_camel_Nigeria_NV2020_2016_Segmentnull_HostCamel.fa | B1228 |
| MG923474_OrganismMiddle_East_respiratory_syndrome_related_coronavirus_Strain_NameMERS_CoV_camel_Nigeria_NV1405_2016_Segmentnull_HostCamel.fa | B1229 |
| MG923480_OrganismMiddle_East_respiratory_syndrome_related_coronavirus_Strain_NameMERS_CoV_camel_Nigeria_NV1787_2016_Segmentnull_HostCamel.fa | B1230 |
| MG923478_OrganismMiddle_East_respiratory_syndrome_related_coronavirus_Strain_NameMERS_CoV_camel_Nigeria_NV1673_2016_Segmentnull_HostCamel.fa | B1231 |
| MG923479_OrganismMiddle_East_respiratory_syndrome_related_coronavirus_Strain_NameMERS_CoV_camel_Nigeria_NV1712_2016_Segmentnull_HostCamel.fa | B1232 |
| MG923477_OrganismMiddle_East_respiratory_syndrome_related_coronavirus_Strain_NameMERS_CoV_camel_Nigeria_NV2040_2016_Segmentnull_HostCamel.fa | B1233 |
| MG923469_OrganismMiddle_East_respiratory_syndrome_related_coronavirus_Strain_NameMERS_CoV_camel_Morocco_CIRAD_HKU213_2015_Segmentnull_HostCamel.fa | B1234 |
| MG923471_OrganismMiddle_East_respiratory_syndrome_related_coronavirus_Strain_NameMERS_CoV_camel_Burkina_Faso_CIRAD_HKU785_2015_Segmentnull_HostCamel.fa | B1235 |
| MG923470_OrganismMiddle_East_respiratory_syndrome_related_coronavirus_Strain_NameMERS_CoV_camel_Burkina_Faso_CIRAD_HKU434_2015_Segmentnull_HostCamel.fa | B1236 |
| KJ477102_OrganismMiddle_East_respiratory_syndrome_coronavirus_Strain_NameNRCE_HKU205_Segmentnull_HostCamel.fa | B1237 |
| MK564474_OrganismMiddle_East_respiratory_syndrome_related_coronavirus_Strain_Namecamel_MERS_Amibara_118_2017_Segmentnull_HostCamel.fa | B1238 |
| MK564475_OrganismMiddle_East_respiratory_syndrome_related_coronavirus_Strain_Namecamel_MERS_Amibara_126_2017_Segmentnull_HostCamel.fa | B1239 |
| MK357908_OrganismMiddle_East_respiratory_syndrome_related_coronavirus_Strain_Name011_DAB_C8_F<1_Segmentnull_HostCamel.fa | B1240 |
| MH734115_OrganismMiddle_East_respiratory_syndrome_related_coronavirus_Strain_NameMERS_CoV_camel_Kenya_C1272_2018_Segmentnull_HostCamel.fa | B1241 |
| MH734114_OrganismMiddle_East_respiratory_syndrome_related_coronavirus_Strain_NameMERS_CoV_camel_Kenya_C1215_2018_Segmentnull_HostCamel.fa | B1242 |
| MK357909_OrganismMiddle_East_respiratory_syndrome_related_coronavirus_Strain_Name011_LOM_C20_F<1_Segmentnull_HostCamel.fa | B1243 |
| MG923466_OrganismMiddle_East_respiratory_syndrome_related_coronavirus_Strain_NameMERS_CoV_camel_Ethiopia_AAU_EPHI_HKU4412_2017_Segmentnull_HostCamel.fa | B1244 |
| MG923468_OrganismMiddle_East_respiratory_syndrome_related_coronavirus_Strain_NameMERS_CoV_camel_Ethiopia_AAU_EPHI_HKU4458_2017_Segmentnull_HostCamel.fa | B1245 |
| MG923467_OrganismMiddle_East_respiratory_syndrome_related_coronavirus_Strain_NameMERS_CoV_camel_Ethiopia_AAU_EPHI_HKU4448_2017_Segmentnull_HostCamel.fa | B1246 |
| JX869059_OrganismHuman_betacoronavirus_2c_EMC_2012_Strain_NameHCoV_EMC_Segmentnull_HostHuman.fa | B1247 |
| NC_019843_OrganismMiddle_East_respiratory_syndrome_related_coronavirus_Strain_NameHCoV_EMC_Segmentnull_HostHuman.fa | B1248 |
| LP963762_OrganismMiddle_East_respiratory_syndrome_related_coronavirus_Strain_NameUNKNOWN_LP963762_Segmentnull_HostUnknown.fa | B1249 |
| KJ614529_OrganismHuman_betacoronavirus_2c_Jordan_N3_2012_Strain_NameJordan_N3_2012_Segmentnull_HostUnknown.fa | B1250 |
| KC776174_OrganismHuman_betacoronavirus_2c_Jordan_N3_2012_Strain_NameJordan_N3_2012_Segmentnull_HostHuman.fa | B1251 |
| KF600620_OrganismMiddle_East_respiratory_syndrome_coronavirus_Strain_NameBisha_1_2012_Segmentnull_HostHuman.fa | B1252 |
| KF600612_OrganismMiddle_East_respiratory_syndrome_coronavirus_Strain_NameRiyadh_1_2012_Segmentnull_HostHuman.fa | B1253 |
| KJ156952_OrganismMiddle_East_respiratory_syndrome_coronavirus_Strain_NameRiyadh_4_2013_Segmentnull_HostHuman.fa | B1254 |
| NC_038294_OrganismBetacoronavirus_England_1_Strain_NameEngland_1_Segmentnull_HostHuman.fa | B1255 |
| KC164505_OrganismBetacoronavirus_England_1_Strain_NameEngland_1_Segmentnull_HostHuman.fa | B1256 |
| KC667074_OrganismHuman_betacoronavirus_2c_England_Qatar_2012_Strain_NameEngland_Qatar_2012_Segmentnull_HostHuman.fa | B1257 |
| KF600613_OrganismMiddle_East_respiratory_syndrome_coronavirus_Strain_NameRiyadh_3_2013_Segmentnull_HostHuman.fa | B1258 |
| KJ156944_OrganismMiddle_East_respiratory_syndrome_coronavirus_Strain_NameRiyadh_5_2013_Segmentnull_HostHuman.fa | B1259 |
| KJ156949_OrganismMiddle_East_respiratory_syndrome_coronavirus_Strain_NameTaif_1_2013_Segmentnull_HostHuman.fa | B1260 |
| KJ156881_OrganismMiddle_East_respiratory_syndrome_coronavirus_Strain_NameWadi_Ad_Dawasir_1_2013_Segmentnull_HostHuman.fa | B1261 |
| KF917527_OrganismMiddle_East_respiratory_syndrome_coronavirus_Strain_NameMERS_CoV_Jeddah_Camel_1_Segmentnull_HostCamel.fa | B1262 |
| KF958702_OrganismMiddle_East_respiratory_syndrome_coronavirus_Strain_NameMERS_CoV_Jeddah_human_1_Segmentnull_HostHuman.fa | B1263 |
| KJ556336_OrganismMiddle_East_respiratory_syndrome_coronavirus_Strain_NameJeddah_1_2013_Segmentnull_HostHuman.fa | B1264 |
| KY688119_OrganismMiddle_East_respiratory_syndrome_related_coronavirus_Strain_NameHu_Aseer_KSA_Rs924_2015_Segmentnull_HostHuman.fa | B1265 |
| KX108944_OrganismMiddle_East_respiratory_syndrome_coronavirus_Strain_NameD1157_15_Segmentnull_HostCamel.fa | B1266 |
| KR011265_OrganismMiddle_East_respiratory_syndrome_coronavirus_Strain_NameHu_Riyadh_KSA_2466_2015_Segmentnull_HostHuman.fa | B1267 |
| KR011263_OrganismMiddle_East_respiratory_syndrome_coronavirus_Strain_NameHu_Riyadh_KSA_2345_2015_Segmentnull_HostHuman.fa | B1268 |
| KR011266_OrganismMiddle_East_respiratory_syndrome_coronavirus_Strain_NameHu_Riyadh_KSA_2049_2015_Segmentnull_HostHuman.fa | B1269 |
| KR011264_OrganismMiddle_East_respiratory_syndrome_coronavirus_Strain_NameHu_Riyadh_KSA_2343_2015_Segmentnull_HostHuman.fa | B1270 |
| KT368875_OrganismMiddle_East_respiratory_syndrome_coronavirus_Strain_Namecamel_Riyadh_Ry179_2015_Segmentnull_HostCamel.fa | B1271 |
| MH259485_OrganismMiddle_East_respiratory_syndrome_related_coronavirus_Strain_NameKSA_1722_Segmentnull_HostCamel.fa | B1272 |
| KT368830_OrganismMiddle_East_respiratory_syndrome_coronavirus_Strain_Namecamel_Jeddah_D34_2014_Segmentnull_HostCamel.fa | B1273 |
| KT368829_OrganismMiddle_East_respiratory_syndrome_coronavirus_Strain_Namecamel_Jeddah_D33 | B1274 |
| KT368832_OrganismMiddle_East_respiratory_syndrome_coronavirus_Strain_Namecamel_Jeddah_D36_2014_Segmentnull_HostCamel.fa | B1275 |
| KT368831_OrganismMiddle_East_respiratory_syndrome_coronavirus_Strain_Namecamel_Jeddah_D35_2014_Segmentnull_HostCamel.fa | B1276 |
| KJ713295_OrganismMiddle_East_respiratory_syndrome_coronavirus_Strain_NameKSA_CAMEL_505_Segmentnull_HostCamel.fa | B1277 |
| KM027261_OrganismMiddle_East_respiratory_syndrome_coronavirus_Strain_NameMakkah_C9355_KSA_Makkah_2014_04_15_Segmentnull_HostHuman.fa | B1278 |
| KM027255_OrganismMiddle_East_respiratory_syndrome_coronavirus_Strain_NameJeddah_C7149_KSA_2014_04_05_Segmentnull_HostHuman.fa | B1279 |
| KM027258_OrganismMiddle_East_respiratory_syndrome_coronavirus_Strain_NameJeddah_C8826_KSA_2014_04_12_Segmentnull_HostHuman.fa | B1280 |
| MK039553_OrganismMiddle_East_respiratory_syndrome_related_coronavirus_Strain_NameHu_Jordan_201440011858_2014_Segmentnull_HostHuman.fa | B1281 |
| MK052676_OrganismMiddle_East_respiratory_syndrome_related_coronavirus_Strain_NameHu_Jordan_201440011858_2014_Segmentnull_HostHuman.fa | B1282 |
| KM027257_OrganismMiddle_East_respiratory_syndrome_coronavirus_Strain_NameJeddah_C7770_KSA_2014_04_07_Segmentnull_HostHuman.fa | B1283 |
| KT861628_OrganismMiddle_East_respiratory_syndrome_coronavirus_Strain_NameHu_Jordan_201440011123_2014_Segmentnull_HostHuman.fa | B1284 |
| MK039552_OrganismMiddle_East_respiratory_syndrome_related_coronavirus_Strain_NameHu_Jordan_201440011123_2014_Segmentnull_HostHuman.fa | B1285 |
| KY581693_OrganismMiddle_East_respiratory_syndrome_related_coronavirus_Strain_NameHu_UAE_032_2014_Segmentnull_HostHuman.fa | B1286 |
| KJ829365_OrganismMiddle_East_respiratory_syndrome_coronavirus_Strain_NameFlorida_USA_2_Saudi_Arabia_2014_Segmentnull_HostHuman.fa | B1287 |
| KP223131_OrganismMiddle_East_respiratory_syndrome_coronavirus_Strain_NameFlorida_USA_2_Saudi_Arabia_2014_Segmentnull_HostHuman.fa | B1288 |
| KM027256_OrganismMiddle_East_respiratory_syndrome_coronavirus_Strain_NameJeddah_C7569_KSA_2014_04_03_Segmentnull_HostHuman.fa | B1289 |
| KM027259_OrganismMiddle_East_respiratory_syndrome_coronavirus_Strain_NameJeddah_C9055_KSA_2014_04_14_Segmentnull_HostHuman.fa | B1290 |
| KM027260_OrganismMiddle_East_respiratory_syndrome_coronavirus_Strain_NameJeddah_C10306_KSA_2014_04_20_Segmentnull_HostHuman.fa | B1291 |
| MH259486_OrganismMiddle_East_respiratory_syndrome_related_coronavirus_Strain_NameKSA_1723_Segmentnull_HostCamel.fa | B1292 |
| KT877351_OrganismMiddle_East_respiratory_syndrome_coronavirus_Strain_NameKSA_1724_Segmentnull_HostCamel.fa | B1293 |
| KT877350_OrganismMiddle_East_respiratory_syndrome_coronavirus_Strain_NameKSA_1725_Segmentnull_HostCamel.fa | B1294 |
| KJ650098_OrganismMiddle_East_respiratory_syndrome_coronavirus_Strain_NameCamel_Qatar_2_2014_Segmentnull_HostCamel.fa | B1295 |
| KJ713296_OrganismMiddle_East_respiratory_syndrome_coronavirus_Strain_NameKSA_CAMEL_378_Segmentnull_HostCamel.fa | B1296 |
| KJ713297_OrganismMiddle_East_respiratory_syndrome_coronavirus_Strain_NameKSA_CAMEL_503_Segmentnull_HostCamel.fa | B1297 |
| MF598663_OrganismMiddle_East_respiratory_syndrome_related_coronavirus_Strain_Namecamel_UAE_B73_2015_Segmentnull_HostCamel.fa | B1298 |
| MF598621_OrganismMiddle_East_respiratory_syndrome_related_coronavirus_Strain_Namecamel_UAE_B29_2015_Segmentnull_HostCamel.fa | B1299 |
| KJ813439_OrganismMiddle_East_respiratory_syndrome_coronavirus_Strain_NameIndiana_USA_1_Saudi_Arabia_2014_Segmentnull_HostHuman.fa | B1300 |
| KM027262_OrganismMiddle_East_respiratory_syndrome_coronavirus_Strain_NameRiyadh_2014KSA_683_KSA_2014_Segmentnull_HostHuman.fa | B1301 |
| KT121574_OrganismMiddle_East_respiratory_syndrome_coronavirus_Strain_NameKFMC_9_Segmentnull_HostHuman.fa | B1302 |
| KT121580_OrganismMiddle_East_respiratory_syndrome_coronavirus_Strain_NameKFMC_1_Segmentnull_HostHuman.fa | B1303 |
| KT121581_OrganismMiddle_East_respiratory_syndrome_coronavirus_Strain_NameKFMC_7_Segmentnull_HostHuman.fa | B1304 |
| KT121578_OrganismMiddle_East_respiratory_syndrome_coronavirus_Strain_NameKFMC_10_Segmentnull_HostHuman.fa | B1305 |
| KT121577_OrganismMiddle_East_respiratory_syndrome_coronavirus_Strain_NameKFMC_2_Segmentnull_HostHuman.fa | B1306 |
| KT121573_OrganismMiddle_East_respiratory_syndrome_coronavirus_Strain_NameKFMC_3_Segmentnull_HostHuman.fa | B1307 |
| KT121575_OrganismMiddle_East_respiratory_syndrome_coronavirus_Strain_NameKFMC_4_Segmentnull_HostHuman.fa | B1308 |
| KT121576_OrganismMiddle_East_respiratory_syndrome_coronavirus_Strain_NameKFMC_6_Segmentnull_HostHuman.fa | B1309 |
| KT121572_OrganismMiddle_East_respiratory_syndrome_coronavirus_Strain_NameKFMC_5_Segmentnull_HostHuman.fa | B1310 |
| KT121579_OrganismMiddle_East_respiratory_syndrome_coronavirus_Strain_NameKFMC_8_Segmentnull_HostHuman.fa | B1311 |
| KT861627_OrganismMiddle_East_respiratory_syndrome_coronavirus_Strain_NameHu_Jordan_20140010168_2014_Segmentnull_HostHuman.fa | B1312 |
| KJ156874_OrganismMiddle_East_respiratory_syndrome_coronavirus_Strain_NameHafr_Al_Batin_6_2013_Segmentnull_HostHuman.fa | B1313 |
| KJ156910_OrganismMiddle_East_respiratory_syndrome_coronavirus_Strain_NameHafr_Al_Batin_2_2013_Segmentnull_HostHuman.fa | B1314 |
| KT368824_OrganismMiddle_East_respiratory_syndrome_coronavirus_Strain_Namecamel_Jeddah_F13A_2014_Segmentnull_HostCamel.fa | B1315 |
| KY689142_OrganismMiddle_East_respiratory_syndrome_related_coronavirus_Strain_NameMERS_Cov_Riyadh_KSA_NGHA__KAMC_2016_Segmentnull_HostHuman.fa | B1316 |
| MH013216_OrganismMiddle_East_respiratory_syndrome_related_coronavirus_Strain_NameHCoV_EMC_Segmentnull_HostHuman.fa | B1317 |
| KF600630_OrganismMiddle_East_respiratory_syndrome_coronavirus_Strain_NameBuraidah_1_2013_Segmentnull_HostHuman.fa | B1318 |
| KJ361502_OrganismMiddle_East_respiratory_syndrome_coronavirus_Strain_NameHu_France___FRA2_130569_2013_InSpu_Sanger_Segmentnull_HostHuman.fa | B1319 |
| KF745068_OrganismMiddle_East_respiratory_syndrome_coronavirus_Strain_NameFRA_UAE_Segmentnull_HostHuman.fa | B1320 |
| KJ361501_OrganismMiddle_East_respiratory_syndrome_coronavirus_Strain_NameHu_France___FRA2_130569_2013_IS_HTS_Segmentnull_HostHuman.fa | B1321 |
| KJ361503_OrganismMiddle_East_respiratory_syndrome_coronavirus_Strain_NameHu_France___FRA2_130569_2013_Isolate_Sanger_Segmentnull_HostHuman.fa | B1322 |
| KJ361500_OrganismMiddle_East_respiratory_syndrome_coronavirus_Strain_NameHu_France_ | B1323 |
| KT156561_OrganismMiddle_East_respiratory_syndrome_coronavirus_Strain_NameHu_Oman_2874_2013_Segmentnull_HostHuman.fa | B1324 |
| KJ650297_OrganismMiddle_East_respiratory_syndrome_coronavirus_Strain_NameKFU_HKU_1_Segmentnull_HostCamel.fa | B1325 |
| KJ650295_OrganismMiddle_East_respiratory_syndrome_coronavirus_Strain_NameKFU_HKU_13_Segmentnull_HostCamel.fa | B1326 |
| KJ650296_OrganismMiddle_East_respiratory_syndrome_coronavirus_Strain_NameKFU_HKU_19Dam_Segmentnull_HostCamel.fa | B1327 |
| KT156560_OrganismMiddle_East_respiratory_syndrome_coronavirus_Strain_NameHu_Oman_2285_2013_Segmentnull_HostHuman.fa | B1328 |
| KY581687_OrganismMiddle_East_respiratory_syndrome_related_coronavirus_Strain_NameHu_UAE_011_2013_Segmentnull_HostHuman.fa | B1329 |
| KP719930_OrganismMiddle_East_respiratory_syndrome_coronavirus_Strain_NameCamel_UAE_D1164.14_2014_Segmentnull_HostCamel.fa | B1330 |
| KP719929_OrganismMiddle_East_respiratory_syndrome_coronavirus_Strain_NameCamel_UAE_D1164.11_2014_Segmentnull_HostCamel.fa | B1331 |
| KX108937_OrganismMiddle_East_respiratory_syndrome_coronavirus_Strain_NameD1164.1_14_Segmentnull_HostCamel.fa | B1332 |
| KP719928_OrganismMiddle_East_respiratory_syndrome_coronavirus_Strain_NameCamel_UAE_D1164.10_2014_Segmentnull_HostCamel.fa | B1333 |
| KP719927_OrganismMiddle_East_respiratory_syndrome_coronavirus_Strain_NameCamel_UAE_D1164.9_2014_Segmentnull_HostCamel.fa | B1334 |
| KP209312_OrganismMiddle_East_respiratory_syndrome_coronavirus_Strain_NameAbu_Dhabi_UAE_9_2013_Segmentnull_HostHuman.fa | B1335 |
| KJ713299_OrganismMiddle_East_respiratory_syndrome_coronavirus_Strain_NameKSA_CAMEL_376_Segmentnull_HostCamel.fa | B1336 |
| KY581684_OrganismMiddle_East_respiratory_syndrome_related_coronavirus_Strain_NameHu_UAE_002_2013_Segmentnull_HostHuman.fa | B1337 |
| KY581685_OrganismMiddle_East_respiratory_syndrome_related_coronavirus_Strain_NameHu_UAE_004_2013_Segmentnull_HostHuman.fa | B1338 |
| KP719933_OrganismMiddle_East_respiratory_syndrome_coronavirus_Strain_NameCamel_UAE_D1209_2014_Segmentnull_HostCamel.fa | B1339 |
| KX108942_OrganismMiddle_East_respiratory_syndrome_coronavirus_Strain_NameD389_15_Segmentnull_HostCamel.fa | B1340 |
| KX108940_OrganismMiddle_East_respiratory_syndrome_coronavirus_Strain_NameD374_15_Segmentnull_HostCamel.fa | B1341 |
| KX108941_OrganismMiddle_East_respiratory_syndrome_coronavirus_Strain_NameD383_15_Segmentnull_HostCamel.fa | B1342 |
| KX108939_OrganismMiddle_East_respiratory_syndrome_coronavirus_Strain_NameD252_15_Segmentnull_HostCamel.fa | B1343 |
| KX108938_OrganismMiddle_East_respiratory_syndrome_coronavirus_Strain_NameD2597.2_14_Segmentnull_HostCamel.fa | B1344 |
| KY581696_OrganismMiddle_East_respiratory_syndrome_related_coronavirus_Strain_NameCamel_UAE_1H_B_2014_Segmentnull_HostCamel.fa | B1345 |
| KP209310_OrganismMiddle_East_respiratory_syndrome_coronavirus_Strain_NameAbu_Dhabi_Gayathi_UAE_2_2014_Segmentnull_HostHuman.fa | B1346 |
| KP209306_OrganismMiddle_East_respiratory_syndrome_coronavirus_Strain_NameAbu_Dhabi_UAE_8_2014_Segmentnull_HostHuman.fa | B1347 |
| KY581692_OrganismMiddle_East_respiratory_syndrome_related_coronavirus_Strain_NameHu_UAE_025_2014_Segmentnull_HostHuman.fa | B1348 |
| KY581691_OrganismMiddle_East_respiratory_syndrome_related_coronavirus_Strain_NameHu_UAE_023_2014_Segmentnull_HostHuman.fa | B1349 |
| KP209311_OrganismMiddle_East_respiratory_syndrome_coronavirus_Strain_NameAbu_Dhabi_UAE_33_2014_Segmentnull_HostHuman.fa | B1350 |
| KP209307_OrganismMiddle_East_respiratory_syndrome_coronavirus_Strain_NameAbu_Dhabi_UAE_18_2014_Segmentnull_HostHuman.fa | B1351 |
| KP209309_OrganismMiddle_East_respiratory_syndrome_coronavirus_Strain_NameAbu_Dhabi_UAE_30_2014_Segmentnull_HostHuman.fa | B1352 |
| KP209313_OrganismMiddle_East_respiratory_syndrome_coronavirus_Strain_NameAbu_Dhabi_UAE_26_2014_Segmentnull_HostHuman.fa | B1353 |
| KY581689_OrganismMiddle_East_respiratory_syndrome_related_coronavirus_Strain_NameHu_UAE_015_2014_Segmentnull_HostHuman.fa | B1354 |
| KP209308_OrganismMiddle_East_respiratory_syndrome_coronavirus_Strain_NameAbu_Dhabi_UAE_16_2014_Segmentnull_HostHuman.fa | B1355 |
| KY581688_OrganismMiddle_East_respiratory_syndrome_related_coronavirus_Strain_NameHu_UAE_011_2014_Segmentnull_HostHuman.fa | B1356 |
| KY581686_OrganismMiddle_East_respiratory_syndrome_related_coronavirus_Strain_NameHu_UAE_009_2014_Segmentnull_HostHuman.fa | B1357 |
| KY581690_OrganismMiddle_East_respiratory_syndrome_related_coronavirus_Strain_NameHu_UAE_017_2014_Segmentnull_HostHuman.fa | B1358 |
| KY673149_OrganismMiddle_East_respiratory_syndrome_related_coronavirus_Strain_NameCamel_Oman_1_2015_Segmentnull_HostCamel.fa | B1359 |
| KY581700_OrganismMiddle_East_respiratory_syndrome_related_coronavirus_Strain_NameCamel_UAE_3B_C_2014_Segmentnull_HostCamel.fa | B1360 |
| KY581697_OrganismMiddle_East_respiratory_syndrome_related_coronavirus_Strain_NameCamel_UAE_1H_D_2014_Segmentnull_HostCamel.fa | B1361 |
| KU242424_OrganismMiddle_East_respiratory_syndrome_coronavirus_Strain_NameCamel_UAE_D469_14_Segmentnull_HostCamel.fa | B1362 |
| KP719932_OrganismMiddle_East_respiratory_syndrome_coronavirus_Strain_NameCamel_UAE_D1243.12_2014_Segmentnull_HostCamel.fa | B1363 |
| KP719931_OrganismMiddle_East_respiratory_syndrome_coronavirus_Strain_NameCamel_UAE_D1339.2_2014_Segmentnull_HostCamel.fa | B1364 |
| KU242423_OrganismMiddle_East_respiratory_syndrome_coronavirus_Strain_NameCamel_UAE_D511_14_Segmentnull_HostCamel.fa | B1365 |
| KY581695_OrganismMiddle_East_respiratory_syndrome_related_coronavirus_Strain_NameCamel_UAE_1B_A_2014_Segmentnull_HostCamel.fa | B1366 |
| KY581698_OrganismMiddle_East_respiratory_syndrome_related_coronavirus_Strain_NameCamel_UAE_1H_F_2014_Segmentnull_HostCamel.fa | B1367 |
| KY581699_OrganismMiddle_East_respiratory_syndrome_related_coronavirus_Strain_NameCamel_UAE_2B_E_2014_Segmentnull_HostCamel.fa | B1368 |
| KY581694_OrganismMiddle_East_respiratory_syndrome_related_coronavirus_Strain_NameHu_UAE_X_2014_Segmentnull_HostHuman.fa | B1369 |
| KF600652_OrganismMiddle_East_respiratory_syndrome_coronavirus_Strain_NameRiyadh_2_2012_Segmentnull_HostHuman.fa | B1370 |
| KM015348_OrganismMiddle_East_respiratory_syndrome_coronavirus_Strain_NameEngland_2_2013_Segmentnull_HostHuman.fa | B1371 |
| KM210277_OrganismMiddle_East_respiratory_syndrome_coronavirus_Strain_NameEngland_4_2013_Segmentnull_HostHuman.fa | B1372 |
| KM210278_OrganismMiddle_East_respiratory_syndrome_coronavirus_Strain_NameEngland_3_2013_Segmentnull_HostHuman.fa | B1373 |
| KJ156869_OrganismMiddle_East_respiratory_syndrome_coronavirus_Strain_NameRiyadh_9_2013_Segmentnull_HostHuman.fa | B1374 |
| KF600634_OrganismMiddle_East_respiratory_syndrome_coronavirus_Strain_NameAl_Hasa_21_2013_Segmentnull_HostHuman.fa | B1375 |
| KF186564_OrganismMiddle_East_respiratory_syndrome_coronavirus_Strain_NameAl_Hasa_4_2013_Segmentnull_HostHuman.fa | B1376 |
| KJ156866_OrganismMiddle_East_respiratory_syndrome_coronavirus_Strain_NameAl_Hasa_25_2013_Segmentnull_HostHuman.fa | B1377 |
| KF600632_OrganismMiddle_East_respiratory_syndrome_coronavirus_Strain_NameAl_Hasa_19_2013_Segmentnull_HostHuman.fa | B1378 |
| KF600647_OrganismMiddle_East_respiratory_syndrome_coronavirus_Strain_NameAl_Hasa_17_2013_Segmentnull_HostHuman.fa | B1379 |
| KF600627_OrganismMiddle_East_respiratory_syndrome_coronavirus_Strain_NameAl_Hasa_12_2013_Segmentnull_HostHuman.fa | B1380 |
| KF186565_OrganismMiddle_East_respiratory_syndrome_coronavirus_Strain_NameAl_Hasa_3_2013_Segmentnull_HostHuman.fa | B1381 |
| KF600651_OrganismMiddle_East_respiratory_syndrome_coronavirus_Strain_NameAl_Hasa_18_2013_Segmentnull_HostHuman.fa | B1382 |
| KF186567_OrganismMiddle_East_respiratory_syndrome_coronavirus_Strain_NameAl_Hasa_1_2013_Segmentnull_HostHuman.fa | B1383 |
| KF600644_OrganismMiddle_East_respiratory_syndrome_coronavirus_Strain_NameAl_Hasa_16_2013_Segmentnull_HostHuman.fa | B1384 |
| KF186566_OrganismMiddle_East_respiratory_syndrome_coronavirus_Strain_NameAl_Hasa_2_2013_Segmentnull_HostHuman.fa | B1385 |
| KF600645_OrganismMiddle_East_respiratory_syndrome_coronavirus_Strain_NameAl_Hasa_15_2013_Segmentnull_HostHuman.fa | B1386 |
| MF598658_OrganismMiddle_East_respiratory_syndrome_related_coronavirus_Strain_Namecamel_UAE_B68_2015_Segmentnull_HostCamel.fa | B1387 |
| MF598678_OrganismMiddle_East_respiratory_syndrome_related_coronavirus_Strain_Namecamel_UAE_B88_2015_Segmentnull_HostCamel.fa | B1388 |
| MF598665_OrganismMiddle_East_respiratory_syndrome_related_coronavirus_Strain_Namecamel_UAE_B75_2015_Segmentnull_HostCamel.fa | B1389 |
| MF598654_OrganismMiddle_East_respiratory_syndrome_related_coronavirus_Strain_Namecamel_UAE_B64_2015_Segmentnull_HostCamel.fa | B1390 |
| MF598687_OrganismMiddle_East_respiratory_syndrome_related_coronavirus_Strain_Namecamel_UAE_B100_2015_Segmentnull_HostCamel.fa | B1391 |
| MF598672_OrganismMiddle_East_respiratory_syndrome_related_coronavirus_Strain_Namecamel_UAE_B82_2015_Segmentnull_HostCamel.fa | B1392 |
| MF598679_OrganismMiddle_East_respiratory_syndrome_related_coronavirus_Strain_Namecamel_UAE_B90_2015_Segmentnull_HostCamel.fa | B1393 |
| MF598667_OrganismMiddle_East_respiratory_syndrome_related_coronavirus_Strain_Namecamel_UAE_B77_2015_Segmentnull_HostCamel.fa | B1394 |
| MF598635_OrganismMiddle_East_respiratory_syndrome_related_coronavirus_Strain_Namecamel_UAE_B44_2015_Segmentnull_HostCamel.fa | B1395 |
| MF598639_OrganismMiddle_East_respiratory_syndrome_related_coronavirus_Strain_Namecamel_UAE_B48_2015_Segmentnull_HostCamel.fa | B1396 |
| MF598636_OrganismMiddle_East_respiratory_syndrome_related_coronavirus_Strain_Namecamel_UAE_B45_2015_Segmentnull_HostCamel.fa | B1397 |
| KY673148_OrganismMiddle_East_respiratory_syndrome_related_coronavirus_Strain_NameHu_Oman_50_2015_Segmentnull_HostHuman.fa | B1398 |
| MF598647_OrganismMiddle_East_respiratory_syndrome_related_coronavirus_Strain_Namecamel_UAE_B56_2015_Segmentnull_HostCamel.fa | B1399 |
| MF598681_OrganismMiddle_East_respiratory_syndrome_related_coronavirus_Strain_Namecamel_UAE_B92_2015_Segmentnull_HostCamel.fa | B1400 |
| MF598637_OrganismMiddle_East_respiratory_syndrome_related_coronavirus_Strain_Namecamel_UAE_B46_2015_Segmentnull_HostCamel.fa | B1401 |
| MF598619_OrganismMiddle_East_respiratory_syndrome_related_coronavirus_Strain_Namecamel_UAE_B27_2015_Segmentnull_HostCamel.fa | B1402 |
| MF598669_OrganismMiddle_East_respiratory_syndrome_related_coronavirus_Strain_Namecamel_UAE_B79_2015_Segmentnull_HostCamel.fa | B1403 |
| MF598648_OrganismMiddle_East_respiratory_syndrome_related_coronavirus_Strain_Namecamel_UAE_B58_2015_Segmentnull_HostCamel.fa | B1404 |
| MF598644_OrganismMiddle_East_respiratory_syndrome_related_coronavirus_Strain_Namecamel_UAE_B53_2015_Segmentnull_HostCamel.fa | B1405 |
| MF598676_OrganismMiddle_East_respiratory_syndrome_related_coronavirus_Strain_Namecamel_UAE_B86_2015_Segmentnull_HostCamel.fa | B1406 |
| MF598695_OrganismMiddle_East_respiratory_syndrome_related_coronavirus_Strain_Namecamel_UAE_B108_2015_Segmentnull_HostCamel.fa | B1407 |
| MF598698_OrganismMiddle_East_respiratory_syndrome_related_coronavirus_Strain_Namecamel_UAE_B111_2015_Segmentnull_HostCamel.fa | B1408 |
| MF598675_OrganismMiddle_East_respiratory_syndrome_related_coronavirus_Strain_Namecamel_UAE_B85_2015_Segmentnull_HostCamel.fa | B1409 |
| MF598624_OrganismMiddle_East_respiratory_syndrome_related_coronavirus_Strain_Namecamel_UAE_B32_2015_Segmentnull_HostCamel.fa | B1410 |
| MF598697_OrganismMiddle_East_respiratory_syndrome_related_coronavirus_Strain_Namecamel_UAE_B110_2015_Segmentnull_HostCamel.fa | B1411 |
| MF598694_OrganismMiddle_East_respiratory_syndrome_related_coronavirus_Strain_Namecamel_UAE_B107_2015_Segmentnull_HostCamel.fa | B1412 |
| MF598594_OrganismMiddle_East_respiratory_syndrome_related_coronavirus_Strain_Namecamel_UAE_B1_2015_Segmentnull_HostCamel.fa | B1413 |
| MF598603_OrganismMiddle_East_respiratory_syndrome_related_coronavirus_Strain_Namecamel_UAE_B11_2015_Segmentnull_HostCamel.fa | B1414 |
| MF598661_OrganismMiddle_East_respiratory_syndrome_related_coronavirus_Strain_Namecamel_UAE_B71_2015_Segmentnull_HostCamel.fa | B1415 |
| KX108945_OrganismMiddle_East_respiratory_syndrome_coronavirus_Strain_NameD1271_15_Segmentnull_HostCamel.fa | B1416 |
| MF598693_OrganismMiddle_East_respiratory_syndrome_related_coronavirus_Strain_Namecamel_UAE_B106_2015_Segmentnull_HostCamel.fa | B1417 |
| MF598633_OrganismMiddle_East_respiratory_syndrome_related_coronavirus_Strain_Namecamel_UAE_B41_2015_Segmentnull_HostCamel.fa | B1418 |
| MF598692_OrganismMiddle_East_respiratory_syndrome_related_coronavirus_Strain_Namecamel_UAE_B105_2015_Segmentnull_HostCamel.fa | B1419 |
| MF598662_OrganismMiddle_East_respiratory_syndrome_related_coronavirus_Strain_Namecamel_UAE_B72_2015_Segmentnull_HostCamel.fa | B1420 |
| MK280984_OrganismMiddle_East_respiratory_syndrome_related_coronavirus_Strain_NameQatar15_Segmentnull_HostHuman.fa | B1421 |
| MN507638_OrganismMiddle_East_respiratory_syndrome_related_coronavirus_Strain_Namellama_passaged_Qatar15_Segmentnull_HostCamel.fa | B1422 |
