# Supplementary material for: Recombination events are concentrated in the spike protein region of Betacoronaviruses
Source: PLoS Genet. 2020 Dec 17;16(12):e1009272. doi: 10.1371/journal.pgen.1009272 (PMC7775116; doi:10.1371/journal.pgen.1009272)
Supplement: S2 Table — (DOCX) [file pgen.1009272.s007.docx]

**S2 Table. List of analyzed genomes and taxonomic information.**

| B636 | LC494185 | Embevovirus | LC494185_OrganismBovine_coronavirus_Strain_NameTCG_26_Segmentnull_HostUnknown.fa |
| --- | --- | --- | --- |
| B661 | LC494177 | Embevovirus | LC494177_OrganismBovine_coronavirus_Strain_NameTCG_9_Segmentnull_HostUnknown.fa |
| B672 | LC494159 | Embevovirus | LC494159_OrganismBovine_coronavirus_Strain_NameSHG_6_Segmentnull_HostUnknown.fa |
| B647 | LC494180 | Embevovirus | LC494180_OrganismBovine_coronavirus_Strain_NameTCG_21_Segmentnull_HostUnknown.fa |
| B659 | LC494158 | Embevovirus | LC494158_OrganismBovine_coronavirus_Strain_NameSHG_5_Segmentnull_HostUnknown.fa |
| B664 | LC494142 | Embevovirus | LC494142_OrganismBovine_coronavirus_Strain_NameIWT_18_Segmentnull_HostUnknown.fa |
| B662 | LC494127 | Embevovirus | LC494127_OrganismBovine_coronavirus_Strain_NameIWT_9_Segmentnull_HostUnknown.fa |
| B658 | LC494126 | Embevovirus | LC494126_OrganismBovine_coronavirus_Strain_NameGIF_1_Segmentnull_HostUnknown.fa |
| B632 | LC494154 | Embevovirus | LC494154_OrganismBovine_coronavirus_Strain_NameSHG_1_Segmentnull_HostUnknown.fa |
| B631 | LC494134 | Embevovirus | LC494134_OrganismBovine_coronavirus_Strain_NameIWT_6_Segmentnull_HostUnknown.fa |
| B674 | LC494129 | Embevovirus | LC494129_OrganismBovine_coronavirus_Strain_NameIWT_1_Segmentnull_HostUnknown.fa |
| B679 | LC494132 | Embevovirus | LC494132_OrganismBovine_coronavirus_Strain_NameIWT_4_Segmentnull_HostUnknown.fa |
| B691 | LC494156 | Embevovirus | LC494156_OrganismBovine_coronavirus_Strain_NameSHG_3_Segmentnull_HostUnknown.fa |
| B686 | LC494133 | Embevovirus | LC494133_OrganismBovine_coronavirus_Strain_NameIWT_5_Segmentnull_HostUnknown.fa |
| B629 | LC494161 | Embevovirus | LC494161_OrganismBovine_coronavirus_Strain_NameTCG_5_Segmentnull_HostUnknown.fa |
| B694 | LC494140 | Embevovirus | LC494140_OrganismBovine_coronavirus_Strain_NameIWT_14_Segmentnull_HostUnknown.fa |
| B701 | MH043952 | Embevovirus | MH043952_OrganismBovine_coronavirus_Strain_Name4_17_03_Segmentnull_HostCattle.fa |
| B702 | MH043955 | Embevovirus | MH043955_OrganismBovine_coronavirus_Strain_Name7_16_23_Segmentnull_HostCattle.fa |
| B700 | MH043953 | Embevovirus | MH043953_OrganismBovine_coronavirus_Strain_Name4_17_25_Segmentnull_HostCattle.fa |
| B715 | DQ915164 | Embevovirus | DQ915164_OrganismBovine_coronavirus_isolate_Alpaca_Strain_NameUNKNOWN_DQ915164_Segmentnull_SubtypeBovine_HostAlpaca.fa |
| B627 | FJ425187 | Embevovirus | FJ425187_OrganismWhite_tailed_deer_coronavirus_US_OH_WD470_1994_Strain_NameUNKNOWN_FJ425187_Segmentnull_HostDeer.fa |
| B699 | KU886219 | Embevovirus | KU886219_OrganismBovine_coronavirus_Strain_NameBCV_AKS_01_Segmentnull_HostCattle.fa |
| B716 | KU558922 | Embevovirus | KU558922_OrganismBetacoronavirus_1_Strain_NameBuffalo_coronavirus_B1_24F_Segmentnull_HostBuffalo.fa |
| B717 | KU558923 | Embevovirus | KU558923_OrganismBetacoronavirus_1_Strain_NameBuffalo_coronavirus_B1_28F_Segmentnull_HostBuffalo.fa |
| B618 | DQ811784 | Embevovirus | DQ811784_OrganismBovine_coronavirus_DB2_Strain_NameDB2_Segmentnull_HostCattle.fa |
| B617 | MG518518 | Embevovirus | MG518518_OrganismWater_deer_coronavirus_Strain_NameW17_18_Segmentnull_HostUnknown.fa |
| B732 | MN514963 | Embevovirus | MN514963_OrganismDromedary_camel_coronavirus_HKU23_Strain_NameDcCoV_HKU23_camel_Morocco_CAC2586_2016_Segmentnull_HostCamel.fa |
| B727 | MN514966 | Embevovirus | MN514966_OrganismDromedary_camel_coronavirus_HKU23_Strain_NameDcCoV_HKU23_camel_Nigeria_NV1097_2015_Segmentnull_HostCamel.fa |
| B728 | MN514967 | Embevovirus | MN514967_OrganismDromedary_camel_coronavirus_HKU23_Strain_NameDcCoV_HKU23_camel_Nigeria_NV1385_2016_Segmentnull_HostCamel.fa |
| B731 | MN514962 | Embevovirus | MN514962_OrganismDromedary_camel_coronavirus_HKU23_Strain_NameDcCoV_HKU23_camel_Ethiopia_CAC1019_2015_Segmentnull_HostCamel.fa |
| B724 | KF906251 | Embevovirus | KF906251_OrganismDromedary_camel_coronavirus_HKU23_Strain_NameHKU23_368F_Segmentnull_HostCamel.fa |
| B718 | KX982264 | Embevovirus | KX982264_OrganismBovine_coronavirus_Strain_NameBCoV_2014_13_Segmentnull_HostCattle.fa |
| B616 | FJ415324 | Embevovirus | FJ415324_OrganismHuman_enteric_coronavirus_4408_Strain_Name4408_Segmentnull_HostHuman.fa |
| B615 | MH810163 | Embevovirus | MH810163_OrganismYak_coronavirus_Strain_NameYAK_HY24_CH_2017_Segmentnull_HostYak.fa |
| B611 | AB354579 | Embevovirus | AB354579_OrganismBovine_coronavirus_Strain_NameKakegawa_Segmentnull_HostUnknown.fa |
| B610 | KX432213 | Embevovirus | KX432213_OrganismCanine_respiratory_coronavirus_Strain_NameBJ232_Segmentnull_HostDog.fa |
| B609 | JX860640 | Embevovirus | JX860640_OrganismCanine_respiratory_coronavirus_Strain_NameK37_Segmentnull_HostDog.fa |
| B365 | AY903460 | Embevovirus | AY903460_OrganismHuman_coronavirus_OC43_Strain_Name19572_Belgium_2004_Segmentnull_HostUnknown.fa |
| B349 | KF923898 | Embevovirus | KF923898_OrganismHuman_coronavirus_OC43_Strain_Name3184A_2012_Segmentnull_HostHuman.fa |
| B747 | KF530079 | Embevovirus | KF530079_OrganismHuman_coronavirus_OC43_Strain_NameOC43_human_USA_913_29_1991_Segmentnull_HostHuman.fa |
| B758 | KF530084 | Embevovirus | KF530084_OrganismHuman_coronavirus_OC43_Strain_NameOC43_human_USA_951_18_1995_Segmentnull_HostHuman.fa |
| B471 | AY391777 | Embevovirus | AY391777_OrganismHuman_coronavirus_OC43_Strain_NameATCC_VR_759_Segmentnull_HostUnknown.fa |
| B733 | KF530092 | Embevovirus | KF530092_OrganismHuman_coronavirus_OC43_Strain_NameOC43_human_USA_008_5_2000_Segmentnull_HostHuman.fa |
| B739 | KY014282 | Embevovirus | KY014282_OrganismHuman_coronavirus_OC43_Strain_Name2007_09_Segmentnull_HostHuman.fa |
| B475 | KF530085 | Embevovirus | KF530085_OrganismHuman_coronavirus_OC43_Strain_NameOC43_human_USA_871_25_1987_Segmentnull_HostHuman.fa |
| B497 | KY419103 | Embevovirus | KY419103_OrganismPorcine_hemagglutinating_encephalomyelitis_virus_Strain_NamePHEV_CoV_USA_15TOSU25049_Segmentnull_HostSwine.fa |
| B498 | KY419104 | Embevovirus | KY419104_OrganismPorcine_hemagglutinating_encephalomyelitis_virus_Strain_NamePHEV_CoV_USA_15TOSU0331_Segmentnull_HostSwine.fa |
| B492 | KY419106 | Embevovirus | KY419106_OrganismPorcine_hemagglutinating_encephalomyelitis_virus_Strain_NamePHEV_CoV_USA_15TOSU1785_Segmentnull_HostSwine.fa |
| B490 | KY994645 | Embevovirus | KY994645_OrganismPorcine_hemagglutinating_encephalomyelitis_virus_Strain_NameJL_2008_Segmentnull_HostSwine.fa |
| B489 | DQ011855 | Embevovirus | DQ011855_OrganismPorcine_hemagglutinating_encephalomyelitis_virus_Strain_NameVW572_Segmentnull_HostUnknown.fa |
| B506 | LC061272 | Embevovirus | LC061272_OrganismEquine_coronavirus_Strain_NameTokachi09_Segmentnull_HostHorse.fa |
| B503 | LC061274 | Embevovirus | LC061274_OrganismEquine_coronavirus_Strain_NameObihiro12_2_Segmentnull_HostHorse.fa |
| B505 | EF446615 | Embevovirus | EF446615_OrganismEquine_coronavirus_Strain_NameNC99_Segmentnull_HostUnknown.fa |
| B507 | JN874561 | Embevovirus | JN874561_OrganismRabbit_coronavirus_HKU14_Strain_NameHKU14_8_Segmentnull_HostRabbit.fa |
| B511 | JN874562 | Embevovirus | JN874562_OrganismRabbit_coronavirus_HKU14_Strain_NameHKU14_10_Segmentnull_HostRabbit.fa |
| B604 | MH687971 | Embevovirus | MH687971_OrganismBetacoronavirus_sp._Strain_NameVZ_BetaCoV_20724_34_c13_Segmentnull_HostRat.fa |
| B603 | MH687969 | Embevovirus | MH687969_OrganismBetacoronavirus_sp._Strain_NameVZ_BetaCoV_20724_33_Segmentnull_HostRat.fa |
| B601 | MH687974 | Embevovirus | MH687974_OrganismBetacoronavirus_sp._Strain_NameVZ_BetaCoV_20724_43_Segmentnull_HostRat.fa |
| B597 | KM349743 | Embevovirus | KM349743_OrganismBetacoronavirus_HKU24_Strain_NameHKU24_R05009I_Segmentnull_HostRat.fa |
| B527 | FJ647221 | Embevovirus | FJ647221_OrganismMurine_coronavirus_repA59_RJHM_Strain_NamerepA59_RJHM_Segmentnull_HostMouse.fa |
| B526 | FJ647220 | Embevovirus | FJ647220_OrganismMurine_coronavirus_RA59_SJHM_Strain_NameRA59_SJHM_Segmentnull_HostMouse.fa |
| B522 | NC_001846 | Embevovirus | NC_001846_OrganismMurine_hepatitis_virus_Strain_NameMHV_A59_Segmentnull_HostUnknown.fa |
| B531 | MF618253 | Embevovirus | MF618253_OrganismMurine_hepatitis_virus_Strain_NameA59_Segmentnull_HostMouse.fa |
| B516 | FJ647222 | Embevovirus | FJ647222_OrganismMurine_coronavirus_SA59_RJHM_Strain_NameSA59_RJHM_Segmentnull_HostMouse.fa |
| B517 | FJ647219 | Embevovirus | FJ647219_OrganismMurine_coronavirus_RJHM_A_Strain_NameRJHM_A_Segmentnull_HostMouse.fa |
| B515 | FJ647227 | Embevovirus | FJ647227_OrganismMurine_coronavirus_repJHM_RA59_Strain_NamerepJHM_RA59_Segmentnull_HostMouse.fa |
| B542 | JF792617 | Embevovirus | JF792617_OrganismRat_coronavirus_Strain_Name8190_Segmentnull_HostRat.fa |
| B541 | JF792616 | Embevovirus | JF792616_OrganismRat_coronavirus_Strain_Name681_Segmentnull_HostRat.fa |
| B539 | GU593319 | Embevovirus | GU593319_OrganismMurine_hepatitis_virus_Strain_NameS_Segmentnull_HostMouse.fa |
| B514 | FJ647224 | Embevovirus | FJ647224_OrganismMurine_coronavirus_MHV_3_Strain_NameMHV_3_Segmentnull_HostMouse.fa |
| B513 | AB551247 | Embevovirus | AB551247_OrganismMurine_hepatitis_virus_Strain_NameMHV_MI_Segmentnull_HostMouse.fa |
| B512 | FJ647223 | Embevovirus | FJ647223_OrganismMurine_coronavirus_MHV_1_Strain_NameMHV_1_Segmentnull_HostMouse.fa |
| B546 | AF207902 | Embevovirus | AF207902_OrganismMurine_hepatitis_virus_strain_ML_11_Strain_NameML_11_Segmentnull_HostUnknown.fa |
| B545 | AF208066 | Embevovirus | AF208066_OrganismMurine_hepatitis_virus_Strain_NamePenn_97_1_Segmentnull_HostUnknown.fa |
| B595 | MH687968 | Embevovirus | MH687968_OrganismBetacoronavirus_sp._Strain_NameVZ_BetaCoV_16715_52_Segmentnull_HostRat.fa |
| B596 | MH687970 | Embevovirus | MH687970_OrganismBetacoronavirus_sp._Strain_NameVZ_BetaCoV_20724_34_c12_Segmentnull_HostRat.fa |
| B561 | KF686343 | Embevovirus | KF686343_OrganismHuman_coronavirus_HKU1_Strain_NameHKU1_human_USA_HKU1_13_2010_Segmentnull_HostHuman.fa |
| B580 | KF686344 | Embevovirus | KF686344_OrganismHuman_coronavirus_HKU1_Strain_NameHKU1_human_USA_HKU1_15_2009_Segmentnull_HostHuman.fa |
| B555 | DQ415898 | Embevovirus | DQ415898_OrganismHuman_coronavirus_HKU1_Strain_NameN21_Segmentnull_HostUnknown.fa |
| B548 | DQ415902 | Embevovirus | DQ415902_OrganismHuman_coronavirus_HKU1_Strain_NameN25_Segmentnull_HostUnknown.fa |
| B550 | MK167038 | Embevovirus | MK167038_OrganismHuman_coronavirus_HKU1_Strain_NameSC2521_Segmentnull_HostHuman.fa |
| B329 | MN611520 | HKU5 | MN611520_OrganismPipistrellus_abramus_bat_coronavirus_HKU5_related_Strain_NameBY140568_Segmentnull_HostBat.fa |
| B330 | MH002340 | HKU5 | MH002340_OrganismPipistrellus_bat_coronavirus_HKU5_Strain_NameBY140535_Segmentnull_HostUnknown.fa |
| B326 | EF065510 | HKU5 | EF065510_OrganismBat_coronavirus_HKU5_2_Strain_NameTT03f_Segmentnull_HostBat.fa |
| B321 | NC_009020 | HKU5 | NC_009020_OrganismPipistrellus_bat_coronavirus_HKU5_Strain_NameHKU5_1_LMH03f_Segmentnull_HostBat.fa |
| B324 | KJ473820 | HKU5 | KJ473820_OrganismBtPa_BetaCoV_GD2013_Strain_NameBtPa_GD2013_Segmentnull_HostBat.fa |
| B325 | MH002341 | HKU5 | MH002341_OrganismPipistrellus_bat_coronavirus_HKU5_Strain_NameBY140562_Segmentnull_HostUnknown.fa |
| B323 | MH002342 | HKU5 | MH002342_OrganismPipistrellus_bat_coronavirus_HKU5_Strain_NameYD13403_Segmentnull_HostUnknown.fa |
| B334 | EF065508 | HKU4 | EF065508_OrganismBat_coronavirus_HKU4_4_Strain_NameLMH1f_Segmentnull_HostBat.fa |
| B333 | MH002339 | HKU4 | MH002339_OrganismTylonycteris_bat_coronavirus_HKU4_Strain_NameSZ140324_Segmentnull_HostUnknown.fa |
| B335 | EF065506 | HKU4 | EF065506_OrganismBat_coronavirus_HKU4_2_Strain_NameB05f_Segmentnull_HostBat.fa |
| B332 | MN611519 | HKU4 | MN611519_OrganismTylonycteris_pachypus_bat_coronavirus_HKU4_related_Strain_NameGZ131656_Segmentnull_HostBat.fa |
| B339 | MH002337 | HKU4 | MH002337_OrganismTylonycteris_bat_coronavirus_HKU4_Strain_NameCZ01_Segmentnull_HostUnknown.fa |
| B340 | MH002338 | HKU4 | MH002338_OrganismTylonycteris_bat_coronavirus_HKU4_Strain_NameCZ07_Segmentnull_HostUnknown.fa |
| B341 | KJ473822 | HKU4 | KJ473822_OrganismBtTp_BetaCoV_GX2012_Strain_NameBtTp_GX2012_Segmentnull_HostBat.fa |
| B345 | KC545386 | Merbecovirus | KC545386_OrganismBetacoronavirus_Erinaceus_VMC_DEU_2012_Strain_NameErinaceusCoV_2012_216_GER_2012_Segmentnull_HostHedgehog.fa |
| B347 | NC_039207 | Merbecovirus | NC_039207_OrganismBetacoronavirus_Erinaceus_VMC_DEU_2012_Strain_NameErinaceusCoV_2012_174_GER_2012_Segmentnull_HostHedgehog.fa |
| B344 | MK679660 | Merbecovirus | MK679660_OrganismHedgehog_coronavirus_1_Strain_NameUNKNOWN_MK679660_Segmentnull_HostHedgehog.fa |
| B342 | MK907287 | Merbecovirus | MK907287_OrganismErinaceus_hedgehog_coronavirus_HKU31_Strain_NameRs13_Segmentnull_HostUnknown.fa |
| B1218 | MG987421 | Merbecovirus | MG987421_OrganismMiddle_East_respiratory_syndrome_related_coronavirus_Strain_NameNL140455_Segmentnull_HostUnknown.fa |
| B1217 | MG987420 | Merbecovirus | MG987420_OrganismMiddle_East_respiratory_syndrome_related_coronavirus_Strain_NameNL13892_Segmentnull_HostUnknown.fa |
| B1216 | KJ473821 | Merbecovirus | KJ473821_OrganismBtVs_BetaCoV_SC2013_Strain_NameUNKNOWN_KJ473821_Segmentnull_HostBat.fa |
| B1215 | MG021451 | Merbecovirus | MG021451_OrganismMiddle_East_respiratory_syndrome_related_coronavirus_Strain_NameNL13845_Segmentnull_HostUnknown.fa |
| B1214 | MG021452 | Merbecovirus | MG021452_OrganismMiddle_East_respiratory_syndrome_related_coronavirus_Strain_NameNL140422_Segmentnull_HostUnknown.fa |
| B1212 | MG596802 | Merbecovirus | MG596802_OrganismMiddle_East_respiratory_syndrome_related_coronavirus_Strain_NameBat_CoV_H.savii_Italy_206645_40_2011_Segmentnull_HostBat.fa |
| B1213 | MG596803 | Merbecovirus | MG596803_OrganismMiddle_East_respiratory_syndrome_related_coronavirus_Strain_NameBat_CoV_P.khulii_Italy_206645_63_2011_Segmentnull_HostBat.fa |
| B1222 | KJ477103 | Merbecovirus | KJ477103_OrganismMiddle_East_respiratory_syndrome_related_coronavirus_Strain_NameNRCE_HKU270_Segmentnull_HostCamel.fa |
| B1240 | MK357908 | Merbecovirus | MK357908_OrganismMiddle_East_respiratory_syndrome_related_coronavirus_Strain_Name011_DAB_C8_F<1_Segmentnull_HostCamel.fa |
| B1 | MF598691 | Merbecovirus | MF598691_OrganismMiddle_East_respiratory_syndrome_related_coronavirus_Strain_Namecamel_UAE_B104_2015_Segmentnull_HostCamel.fa |
| B1221 | KX108943 | Merbecovirus | KX108943_OrganismMiddle_East_respiratory_syndrome_coronavirus_Strain_NameD998_15_Segmentnull_HostCamel.fa |
| B1219 | KC869678 | Merbecovirus | KC869678_OrganismCoronavirus_Neoromicia_PML_PHE1_RSA_2011_Strain_NameNeoromicia_PML_PHE1_RSA_2011_Segmentnull_HostBat.fa |
| B1220 | MF593268 | Merbecovirus | MF593268_OrganismMiddle_East_respiratory_syndrome_related_coronavirus_Strain_NameNeoromicia_5038_Segmentnull_HostBat.fa |
| B765 | HM211098 | Nobecovirus | HM211098_OrganismBat_coronavirus_HKU9_5_1_Strain_NameUNKNOWN_HM211098_Segmentnull_HostBat.fa |
| B764 | EF065515 | Nobecovirus | EF065515_OrganismBat_coronavirus_HKU9_3_Strain_NameBF_493I_Segmentnull_HostBat.fa |
| B766 | HM211100 | Nobecovirus | HM211100_OrganismBat_coronavirus_HKU9_10_1_Strain_NameUNKNOWN_HM211100_Segmentnull_HostBat.fa |
| B767 | NC_009021 | Nobecovirus | NC_009021_OrganismRousettus_bat_coronavirus_HKU9_Strain_NameHKU9_1_BF_005I_Segmentnull_HostBat.fa |
| B769 | EF065516 | Nobecovirus | EF065516_OrganismBat_coronavirus_HKU9_4_Strain_NameBF_141I_Segmentnull_HostBat.fa |
| B763 | MG762674 | Nobecovirus | MG762674_OrganismRousettus_bat_coronavirus_HKU9_Strain_NameRousettus_spp_Jinghong_2009_Segmentnull_HostBat.fa |
| B770 | HM211099 | Nobecovirus | HM211099_OrganismBat_coronavirus_HKU9_5_2_Strain_NameUNKNOWN_HM211099_Segmentnull_HostBat.fa |
| B772 | HM211101 | Nobecovirus | HM211101_OrganismBat_coronavirus_HKU9_10_2_Strain_NameUNKNOWN_HM211101_Segmentnull_HostBat.fa |
| B771 | EF065514 | Nobecovirus | EF065514_OrganismBat_coronavirus_HKU9_2_Strain_NameBF_017I_Segmentnull_HostBat.fa |
| B773 | MK211379 | Nobecovirus | MK211379_OrganismCoronavirus_BtRt_BetaCoV_GX2018_Strain_NameBtRt_BetaCoV_GX2018_Segmentnull_HostBat.fa |
| B776 | KF636752 | Hibecovirus | KF636752_OrganismBat_Hp_betacoronavirus_Zhejiang2013_Strain_NameZhejiang2013_Segmentnull_HostBat.fa |
| B781 | MT084071 | Sarbecovirus | MT084071_OrganismPangolin_coronavirus_Strain_NameMP789_Segmentnull_HostUnknown.fa |
| B782 | MT106054 | Sarbecovirus | MT106054_OrganismSevere_acute_respiratory_syndrome_coronavirus_2_Strain_Name2019_nCoV_USA_TX1_2020_Segmentnull_HostHuman.fa |
| B779 | MG772933 | Sarbecovirus | MG772933_OrganismBat_SARS_like_coronavirus_Strain_Namebat_SL_CoVZC45_Segmentnull_HostBat.fa |
| B780 | MG772934 | Sarbecovirus | MG772934_OrganismBat_SARS_like_coronavirus_Strain_Namebat_SL_CoVZXC21_Segmentnull_HostBat.fa |
| B778 | KY352407 | Sarbecovirus | KY352407_OrganismSevere_acute_respiratory_syndrome_related_coronavirus_Strain_NameBtKY72_Segmentnull_HostBat.fa |
| B876 | DQ084200 | Sarbecovirus | DQ084200_OrganismBat_SARS_coronavirus_HKU3_3_Strain_NameHKU3_3_Segmentnull_HostUnknown.fa |
| B875 | GQ153547 | Sarbecovirus | GQ153547_OrganismBat_SARS_coronavirus_HKU3_12_Strain_NameHKU3_12_Segmentnull_HostUnknown.fa |
| B873 | GQ153542 | Sarbecovirus | GQ153542_OrganismBat_SARS_coronavirus_HKU3_7_Strain_NameHKU3_7_Segmentnull_HostUnknown.fa |
| B872 | KF294457 | Sarbecovirus | KF294457_OrganismSARS_related_bat_coronavirus_Strain_NameLongquan_140_Segmentnull_HostBat.fa |
| B894 | DQ412042 | Sarbecovirus | DQ412042_OrganismBat_SARS_CoV_Rf1_2004_Strain_NameRf1_Segmentnull_HostBat.fa |
| B893 | DQ648856 | Sarbecovirus | DQ648856_OrganismBat_CoV_273_2005_Strain_NameBtCoV_273_2005_Segmentnull_HostUnknown.fa |
| B891 | KJ473812 | Sarbecovirus | KJ473812_OrganismBtRf_BetaCoV_HeB2013_Strain_NameBtRf_HeB2013_Segmentnull_HostBat.fa |
| B890 | KJ473811 | Sarbecovirus | KJ473811_OrganismBtRf_BetaCoV_JL2012_Strain_NameBtRf_JL2012_Segmentnull_HostBat.fa |
| B889 | JX993987 | Sarbecovirus | JX993987_OrganismBat_coronavirus_Rp_Shaanxi2011_Strain_NameRp_Shaanxi2011_Segmentnull_HostBat.fa |
| B886 | KJ473814 | Sarbecovirus | KJ473814_OrganismBtRs_BetaCoV_HuB2013_Strain_NameBtRs_HuB2013_Segmentnull_HostBat.fa |
| B887 | DQ648857 | Sarbecovirus | DQ648857_OrganismBat_CoV_279_2005_Strain_NameBtCoV_279_2005_Segmentnull_HostUnknown.fa |
| B1210 | MK211374 | Sarbecovirus | MK211374_OrganismCoronavirus_BtRl_BetaCoV_SC2018_Strain_NameBtRl_BetaCoV_SC2018_Segmentnull_HostBat.fa |
| B896 | KF569996 | Sarbecovirus | KF569996_OrganismRhinolophus_affinis_coronavirus_Strain_NameLYRa11_Segmentnull_HostBat.fa |
| B897 | JX993988 | Sarbecovirus | JX993988_OrganismBat_coronavirus_Cp_Yunnan2011_Strain_NameCp_Yunnan2011_Segmentnull_HostBat.fa |
| B895 | KU973692 | Sarbecovirus | KU973692_OrganismSARS_related_coronavirus_Strain_NameF46_Segmentnull_HostBat.fa |
| B898 | DQ071615 | Sarbecovirus | DQ071615_OrganismBat_SARS_CoV_Rp3_2004_Strain_NameRp3_Segmentnull_HostBat.fa |
| B899 | KJ473815 | Sarbecovirus | KJ473815_OrganismBtRs_BetaCoV_GX2013_Strain_NameBtRs_GX2013_Segmentnull_HostBat.fa |
| B1207 | KJ473816 | Sarbecovirus | KJ473816_OrganismBtRs_BetaCoV_YN2013_Strain_NameBtRs_YN2013_Segmentnull_HostBat.fa |
| B1206 | KY417145 | Sarbecovirus | KY417145_OrganismBat_SARS_like_coronavirus_Strain_NameRf4092_Segmentnull_HostBat.fa |
| B1208 | KP886808 | Sarbecovirus | KP886808_OrganismBat_SARS_like_coronavirus_YNLF_31C_Strain_NameYNLF_31C_Segmentnull_HostBat.fa |
| B1203 | MK211375 | Sarbecovirus | MK211375_OrganismCoronavirus_BtRs_BetaCoV_YN2018A_Strain_NameBtRs_BetaCoV_YN2018A_Segmentnull_HostBat.fa |
| B1205 | KY417142 | Sarbecovirus | KY417142_OrganismBat_SARS_like_coronavirus_Strain_NameAs6526_Segmentnull_HostBat.fa |
| B1204 | MK211377 | Sarbecovirus | MK211377_OrganismCoronavirus_BtRs_BetaCoV_YN2018C_Strain_NameBtRs_BetaCoV_YN2018C_Segmentnull_HostBat.fa |
| B1197 | KY417147 | Sarbecovirus | KY417147_OrganismBat_SARS_like_coronavirus_Strain_NameRs4237_Segmentnull_HostBat.fa |
| B1198 | KY417148 | Sarbecovirus | KY417148_OrganismBat_SARS_like_coronavirus_Strain_NameRs4247_Segmentnull_HostBat.fa |
| B1202 | KY417143 | Sarbecovirus | KY417143_OrganismBat_SARS_like_coronavirus_Strain_NameRs4081_Segmentnull_HostBat.fa |
| B1201 | KY417149 | Sarbecovirus | KY417149_OrganismBat_SARS_like_coronavirus_Strain_NameRs4255_Segmentnull_HostBat.fa |
| B1199 | FJ588686 | Sarbecovirus | FJ588686_OrganismSARS_coronavirus_Rs_672_2006_Strain_NameRs672_Segmentnull_HostBat.fa |
| B1200 | MK211378 | Sarbecovirus | MK211378_OrganismCoronavirus_BtRs_BetaCoV_YN2018D_Strain_NameBtRs_BetaCoV_YN2018D_Segmentnull_HostBat.fa |
| B1192 | KY417152 | Sarbecovirus | KY417152_OrganismBat_SARS_like_coronavirus_Strain_NameRs9401_Segmentnull_HostBat.fa |
| B1191 | KY417151 | Sarbecovirus | KY417151_OrganismBat_SARS_like_coronavirus_Strain_NameRs7327_Segmentnull_HostBat.fa |
| B1190 | MK211376 | Sarbecovirus | MK211376_OrganismCoronavirus_BtRs_BetaCoV_YN2018B_Strain_NameBtRs_BetaCoV_YN2018B_Segmentnull_HostBat.fa |
| B1196 | KY417144 | Sarbecovirus | KY417144_OrganismBat_SARS_like_coronavirus_Strain_NameRs4084_Segmentnull_HostBat.fa |
| B1195 | KC881005 | Sarbecovirus | KC881005_OrganismBat_SARS_like_coronavirus_RsSHC014_Strain_NameRsSHC014_Segmentnull_HostBat.fa |
| B1193 | KF367457 | Sarbecovirus | KF367457_OrganismBat_SARS_like_coronavirus_WIV1_Strain_NameWIV1_Segmentnull_HostBat.fa |
| B1187 | KY417146 | Sarbecovirus | KY417146_OrganismBat_SARS_like_coronavirus_Strain_NameRs4231_Segmentnull_HostBat.fa |
| B1188 | KT444582 | Sarbecovirus | KT444582_OrganismSARS_like_coronavirus_WIV16_Strain_NameWIV16_Segmentnull_HostBat.fa |
| B900 | AY427439 | Sarbecovirus | AY427439_OrganismSARS_coronavirus_AS_Strain_NameAS_Segmentnull_HostHuman.fa |
| B774 | FV537210 | Sarbecovirus | FV537210_OrganismSARS_coronavirus_Strain_NameModified_Microbial_Nucleic_Acid_Segmentnull_HostUnknown.fa |
| B1211 | HI553383 | Sarbecovirus | HI553383_OrganismSARS_coronavirus_Strain_NameSequence_41_from_Patent_EP2139515_Segmentnull_HostUnknown.fa |
