# Supplementary material for: Recombination events are concentrated in the spike protein region of Betacoronaviruses
Source: PLoS Genet. 2020 Dec 17;16(12):e1009272. doi: 10.1371/journal.pgen.1009272 (PMC7775116; doi:10.1371/journal.pgen.1009272)
Supplement: S3 Table — (DOCX) [file pgen.1009272.s008.docx]

**S3 Table. List of SARS-CoV-2 genomes analyzed.**

| LC522972 |
| --- |
| LC522973 |
| LC522974 |
| LC528232 |
| LC528233 |
| LC529905 |
| LC534418 |
| LC534419 |
| LR757995 |
| LR757996 |
| LR757998 |
| MN938384 |
| MN975262 |
| MN985325 |
| MN988713 |
| MN994467 |
| MN994468 |
| MN996527 |
| MN996529 |
| MN996531 |
| MN997409 |
| MT007544 |
| MT012098 |
| MT019529 |
| MT019531 |
| MT019533 |
| MT027062 |
| MT027064 |
| MT039887 |
| MT039888 |
| MT039890 |
| MT044257 |
| MT044258 |
| MT049951 |
| MT050493 |
| MT066156 |
| MT066176 |
| MT072688 |
| MT093571 |
| MT106052 |
| MT106053 |
| MT106054 |
| MT118835 |
| MT121215 |
| MT123290 |
| MT123291 |
| MT123292 |
| MT123293 |
| MT126808 |
| MT135041 |
| MT135043 |
| MT152824 |
| MT159705 |
| MT159706 |
| MT159707 |
| MT159708 |
| MT159709 |
| MT159712 |
| MT159715 |
| MT159716 |
| MT159717 |
| MT159718 |
| MT159720 |
| MT159722 |
| MT163716 |
| MT163717 |
| MT163719 |
| MT184908 |
| MT184910 |
| MT184911 |
| MT184913 |
| MT188339 |
| MT188340 |
| MT188341 |
| MT192765 |
| MT192772 |
| MT198652 |
| MT226610 |
| MT233519 |
| MT233522 |
| MT233523 |
| MT240479 |
| MT246449 |
| MT246451 |
| MT246452 |
| MT246453 |
| MT246454 |
| MT246455 |
| MT246456 |
| MT246457 |
| MT246458 |
| MT246459 |
| MT246461 |
| MT246462 |
| MT246464 |
| MT246467 |
| MT246468 |
| MT246469 |
| MT246470 |
| MT246471 |
| MT246472 |
| MT246473 |
| MT246474 |
| MT246475 |
| MT246476 |
| MT246477 |
| MT246478 |
| MT246480 |
| MT246482 |
| MT246484 |
| MT246485 |
| MT246486 |
| MT251972 |
| MT251973 |
| MT251975 |
| MT251976 |
| MT251977 |
| MT251978 |
| MT251980 |
| MT253706 |
| MT258377 |
| MT258379 |
| MT258380 |
| MT258381 |
| MT258382 |
| MT258383 |
| MT259226 |
| MT259227 |
| MT259228 |
| MT259230 |
| MT259235 |
| MT259236 |
| MT259237 |
| MT259247 |
| MT259248 |
| MT259249 |
| MT259250 |
| MT259252 |
| MT259253 |
| MT259256 |
| MT259257 |
| MT259261 |
| MT259267 |
| MT259269 |
| MT259271 |
| MT259273 |
| MT259274 |
| MT259277 |
| MT259280 |
| MT259281 |
| MT259282 |
| MT259284 |
| MT259285 |
| MT259287 |
| MT262896 |
| MT262899 |
| MT262915 |
| MT262993 |
| MT263074 |
| MT263384 |
| MT263386 |
| MT263387 |
| MT263388 |
| MT263395 |
| MT263398 |
| MT263399 |
| MT263400 |
| MT263403 |
| MT263404 |
| MT263405 |
| MT263406 |
| MT263408 |
| MT263410 |
| MT263411 |
| MT263412 |
| MT263413 |
| MT263414 |
| MT263415 |
| MT263416 |
| MT263418 |
| MT263419 |
| MT263420 |
| MT263421 |
| MT263422 |
| MT263424 |
| MT263426 |
| MT263428 |
| MT263429 |
| MT263430 |
| MT263431 |
| MT263433 |
| MT263434 |
| MT263435 |
| MT263436 |
| MT263438 |
| MT263439 |
| MT263440 |
| MT263441 |
| MT263443 |
| MT263445 |
| MT263447 |
| MT263448 |
| MT263450 |
| MT263451 |
| MT263452 |
| MT263453 |
| MT263454 |
| MT263455 |
| MT263457 |
| MT263459 |
| MT263462 |
| MT263463 |
| MT263468 |
| MT276323 |
| MT276324 |
| MT276327 |
| MT276328 |
| MT276331 |
